# From Early Micro-Temporal Interaction Patterns to Child Cortisol Levels: Toward the Role of Interactive Reparation and Infant Attachment in a Longitudinal Study

**DOI:** 10.3389/fpsyg.2021.807157

**Published:** 2022-01-20

**Authors:** Mitho Müller, Anna-Lena Zietlow, Nathania Klauser, Christian Woll, Nora Nonnenmacher, Edward Tronick, Corinna Reck

**Affiliations:** ^1^EEKIP-Lab, Clinical Psychology in Childhood and Adolescence, Department Psychology, Ludwig Maximilian University of Munich, Munich, Germany; ^2^Department of Psychology, School of Social Sciences, University of Mannheim, Mannheim, Germany; ^3^Institute of Medical Psychology, Center for Psychosocial Medicine, Heidelberg University Hospital, Heidelberg, Germany; ^4^Child Development Unit, Developmental Brain Sciences Program, Department of Psychology, University of Massachusetts Boston – Harvard Medical School, Boston, MA, United States

**Keywords:** maternal anxiety disorder, still-face, interactive reparation, infant attachment, child cortisol reactivity

## Abstract

Parental mental disorders increase the risk for insecure attachment in children. However, the quality of caregiver–infant interaction plays a key role in the development of infant attachment. Dyadic interaction is frequently investigated via global scales which are too rough to uncover micro-temporal mechanisms. Prior research found that the latency to reparation of uncoordinated dyadic states is associated with infant behavioral and neuroendocrine regulation. We investigated the hypothesis that this interactive mechanism is critical in predicting secure vs. insecure attachment quality in infancy. We also assessed the predictive quality of infant attachment regarding neuroendocrine reactivity later in childhood. A subsample of *N* = 58 dyads (*n* = 22 mothers with anxiety disorders, *n* = 36 controls) from a larger study were analyzed. At 3–8 months postpartum, maternal anxiety disorders were diagnosed via a structured clinical interview as well as dyadic interaction during the *Face-to-Face-Still-Face* (FFSF) was observed and coded on a micro-temporal scale. Infant attachment quality was assessed with the strange situation paradigm at 12–24 months of age. In an overlapping subsample of *N* = 39 (*n* = 13 mothers with anxiety disorder; *n* = 26 controls), we assessed child cortisol reactivity at 5 to 6 years of age. Generalized linear modeling revealed that longer latencies to interactive reparation during the reunion episode of the FFSF as well as maternal diagnosis at 3–8 months of age predict insecure attachment in children aged 12–24 months. Cox regressions demonstrated that dyads with infants who developed insecure attachment at 12–24 months of age were 48% less likely to achieve an interactive reparation at 3–8 months of age. Mixed models revealed that compared to securely attached children, children who had developed an insecure attachment at 12–24 months of age had an increased cortisol reactivity at 5 to 6 years of age during free play. The results confirm the hypothesis that the development of attachment is affected by experienced micro-temporal interactive patterns besides diagnostic categories. They also showed that infants of mothers with postpartum anxiety disorders have a more than fivefold increased risk of developing an insecure attachment than the infants of the control group. Moreover, results imply that these patterns may influence neurohormonal regulation even in preschool aged children.

## Introduction

Attachment theory describes the inherent human need to establish close relationships to other humans from the perspective of the emotional needs of infants. Attachment is discussed as the evolutionary established ensuring of child survival, since the human offspring is specifically in need of long-term care and help (for an overview, see Bowlby, 1969/1982, 1973, 1980). Ainsworth developed the strange situation – an observational experiment for classifying secure, insecure-avoidant and insecure-ambivalent attachment styles (Ainsworth et al., 1978), as well as the later defined disorganized attachment (Main and Solomon, 1986). However, it is not only the relationship quality between children and their parents that is determined by the attachment style. For decades now, the scientific literature has also demonstrated the multi-facetted long-term effects of secure vs. insecure attachment for child development.

To mention a few recent results, securely attached infants manifest higher capacities in processing social information than insecurely attached infants (Biro et al., 2015). The latter exhibit a higher increase in cortisol levels than their securely attached counterparts following the strange situation (Luijk et al., 2010). Securely attached children demonstrate vagal adaption to external demands, such as social stressors, while insecurely attached children do not (Paret et al., 2015). Additionally, Bernard and Dozier (2010) detected a cortisol response following the strange situation for disorganized and not for children with a secure attachment quality. Later in life, securely attached adolescents show a higher empathetic responsiveness (Diamond et al., 2012), whereas insecurely attached children, adolescents and adults exhibit difficulties in regulating stress, more specifically, they show signs for a dysregulation of the hypothalamus–pituitary–adrenal (HPA) axis (Oskis et al., 2011; Pierrehumbert et al., 2012; Kidd et al., 2013). In their review, Beatson and Taryan (2003) concluded that secure attachment serves as a buffer in the relationship between HPA dysregulation and the development of depressive symptoms later in life. Thus, one can conclude “early attachment quality may be a lasting source of vulnerability or protection in children’s development” (Carlone and Milan, 2021, p. 603).

Waters et al. (2010) explored the ties between attachment and emotion regulation. They emphasized the importance of emotion understanding in the development of these constructs. Furthermore, Kerns and Brumariu (2014) discussed insecure and disorganized attachment as risk factors for the development of affective disorders and that this association might trace back, in part, to less competent emotion regulation capacities in insecurely attached children. A hypotheses that was recently supported by Verhees et al. (2021), who found a mediation pathway between attachment insecurity, the regulation of positive, as well as negative affect and the development of depressive symptoms in a large longitudinal sample of adolescents.

Emotion regulation capacities are hypothesized to be formed by social interactions children experience in their everyday life (Beeghly and Tronick, 2011). Also, Ainsworth emphasized the importance of caregiving behavior, specifically the caregiver’s sensitivity, for the development of a secure attachment. Ainsworth defined sensitivity as the caregiver’s ability to perceive, correctly interpret, as well as to promptly and adequately respond to the infant’s communicative signals (Ainsworth et al., 1978). Braungart-Rieker et al. (2001) found that both infant affect regulation and maternal sensitivity discriminate between secure and insecure infants and that the association between sensitivity and attachment was partially mediated by infant regulation. Besides these associations to infant attachment (e.g., Fuertes et al., 2009), parental sensitivity has been shown to be of relevance for a wide range of further developmental outcomes, such as the processing of social information (Biro et al., 2015), fear reactivity (Braungart-Rieker et al., 2010), physiological (Moore et al., 2009; Conradt and Ablow, 2010), neuroendocrine (Spangler et al., 1994; Jansen et al., 2010b) and affective regulation (Haley and Stansbury, 2003; Jonas et al., 2015; Rodrigues et al., 2021), social behavior (Kivijärvi et al., 2001; Bernier et al., 2021; Licata-Dandel et al., 2021), as well as cognitive and language development (Malmberg et al., 2016; Rodrigues et al., 2021).

However, as much as the concept of parental sensitivity was and is needed to understand infant attachment, it is both multidimensional and a somewhat rough macro characteristic. Thus, it is limited in uncovering the details of the moment-to-moment interactive mechanisms that may be important in forming a secure attachment throughout the interactive history of a child (compare to Mesman, 2010). One such mechanism may be derived from Tronick’s reparation model (Tronick, 2007). In this model, Tronick describes the micro-temporal regulation of affect and distress in caregiver–infant dyads. The interactive partners are described as open and dynamic systems (Ham and Tronick, 2009; DiCorcia and Tronick, 2011), whose interactive states are interdependent, especially in young infants. As their self-regulatory capacities are limited, they rely on the regulatory input of their caregivers. It is a complimentary expanded view shared by other theoretical frameworks (see Cole et al., 2004) that suggest a developmental sequence of increasing self-regulatory capacities. In this sequence, young infants have basic regulatory skills of limited effectiveness (compare to Diener and Mangelsdorf, 1999), then interactively engage with their caregivers who represent external resources of regulation (Spangler et al., 1994), and finally develop more competent self-regulatory strategies throughout their development. The interactive process is asymmetric (e.g., Beebe et al., 2016) as it is largely led by the caregivers (Cohn and Tronick, 1988) which is somewhat due to the limited capacities of infants. However, in this process, the role of infant interactive behavior is essential as they communicate their biobehavioral status by means of eye-contact, facial expressions, body postures, vocalizations, etc. and consequently invite the caregivers to regulatory scaffolding. The caregivers, in turn, may perceive, correctly interpret, as well as promptly and adequately respond to the infant’s signals (compare “sensitivity,” Ainsworth et al., 1978), and thus externally regulate the biobehavioral status of their infant. According to Tronick (1989) this process is mutually regulated.

Paradoxically, this regulatory process in itself is stressful. Due to misinterpretations of the caregiver or the limited interactive capacities of the infant, the speed of exchange, etc., uncoordinated dyadic states (so-called mismatches) repeatedly occur in small time intervals. These mismatches produce inconsistencies between the regulatory need of the infant and the regulatory input of the caregiver, which Tronick describes as micro-stressors. These stressors are overcome as soon as the caregiver is able to provide a regulatory input that corresponds to the infant’s regulatory need, or the infant adjusts to the caregiver’s actions – a process called interactive reparation. Thus, the reparation model describes the dyadic regulation as mutual adaptive process in which the dyad oscillates between coordinated states (matches) and mismatches on a micro-temporal scale. It is this dynamic process, which is thought to shape not only infant regulatory strategies but also a wide range of developmental domains, such as attachment (DiCorcia and Tronick, 2011). Indeed, interactive reparation was demonstrated to be associated to infant neuroendocrine (Müller et al., 2015) and psychological regulation (Provenzi et al., 2015). Furthermore, Beebe et al. (2010) revealed, that a moderate level of interactive contingency, which may be described as the occurrence of mismatches that are quickly repaired to matches, predicted infant secure attachment, whereas both low (failure to reparation) and high (few mismatches) levels of contingency predicted insecure attachment. This result fits well with the idea, that a perfectly matched interactive pattern between caregivers and children is neither possible nor desirable as it would prohibit the opportunities to internalize dyadically scaffolded regulation strategies by transforming micro-temporal stressors into non-stressful states (DiCorcia and Tronick, 2011). Nonetheless, to the best of the author’s knowledge, the role of interactive reparation regarding the development of secure vs. insecure attachment has not been investigated. As Provenzi et al. (2018) state, “more research on the interconnections between macro-analytical concepts in caregiver–infant research, such as sensitivity and attachment, and micro-analytical processes is desirable” as “[…] future investigations on the relations between macro- and micro-analytical concepts would not only connect different methodological approaches but also enhance our understanding of the dynamics in developmental trajectories” (page 18).

Besides the associations between parental sensitivity and infant and child attachment, it is well known, that parental mental disorders may lead to unfavorable effects on child behavioral (Kingston and Tough, 2014), cognitive (Murray et al., 2003) and psychopathological (Goodman et al., 2011) development and that parental psychopathology may interfere with the development of a secure attachment style (Wan and Green, 2009). Although most studies have concentrated on maternal depression (e.g., Goodman et al., 2011) there is also some empirical and growing evidence about the associations between parental anxiety and child development (Glasheen et al., 2010; Goodman et al., 2016; Reck et al., 2018b; Polte et al., 2019). Specifically, for this current study and to the best of the authors’ knowledge, there is only one study demonstrating that maternal anxiety may predict insecure attachment in children (Stevenson-Hinde et al., 2011). As it is assumed that the attachment quality and regulatory capacities are associated to and organized by interactive history, it is only natural to conclude, that the association between parental anxiety and child attachment would be mediated by interactive characteristics. Indeed, dyads in which the mother suffers from anxiety show specific problematic interactive patterns (for an overview see Kaitz and Maytal, 2005; Goodman et al., 2016). Besides reduced maternal sensitivity (Warren et al., 2003; Kertz et al., 2008; Feldman et al., 2009; Stevenson-Hinde et al., 2013), dyads with anxious mothers also show micro-temporal differences compared to non-anxious mothers, as for example less contingent maternal interactive patterns (Beebe et al., 2011), as well as changed infant patterns of positive and negative affective displays (Reck et al., 2018a).

However, compared to the effects of parental depression, the evidence regarding the effects of parental anxiety on interactive patterns is less consistent. Several studies identify specific rather than general interactive impairments in dyads with anxious mothers: Grant et al. (2009), for example, did not find an association between maternal anxiety and maternal sensitivity. A finding that is shared by the results of Murray et al. (2007) regarding mothers with social phobia. However, they specifically observed that the mothers were more anxious and were less engaging when interacting with a stranger. Moreover, they encouraged their infants less to interact with the stranger. Additionally, the infants of mothers with social phobia were less responsive to the stranger. The results of Murray et al. (2012) did not reflect general differences regarding interactive patterns between dyads with social phobic mothers and controls in a non-threat interaction task, too. Contrary, in disorder-specific challenges, some parenting difficulties were observable for the clinical group. These difficulties, however, did not seem disorder specific. Accordingly, Kertz et al. (2008) report, that anxious mothers only demonstrate less sensitivity in social tasks. Hence, it may be erroneous to assume general interactive deficits in these dyads. Results suggest the associations between maternal anxiety and child behavioral regulation along with mental development are moderated by caregiving behaviors (as shown for sensitivity in Grant et al., 2010a,b). Notably, these studies refer to prepartum anxiety and their results are discussed considering the fetal programming hypothesis (see van den Bergh et al., 2017). However, we suggest the applicability of this idea to the postpartum period as shown by Kertz et al. (2008) as well as Richter and Reck (2013) for infant regulatory problems and aim to control for prepartum effects in our models in particular. It also seems highly unlikely that the associations between child attachment and long-term consequences are mono-causal. They are rather more likely determined by mutually moderating risk-constellations and factors. For example, the effect of insecure attachment on cortisol response seems more pronounced in infants of depressed mothers (Luijk et al., 2010). Furthermore, insecurely and disorganized attached children seem more prone to develop behavioral and cognitive deficits when exposed to parenting distress or maternal depression than securely attached children (Tharner et al., 2012; Carlone and Milan, 2021).

The aim of this study is to identify the most important predictors for (1) the development of insecure vs. secure attachment considering the effects of micro-temporal reparation processes along with parental anxiety in the postpartum period, prepartum distress and their interaction effects, and (2) child cortisol-reactivity considering the long-term effects of infant secure vs. insecure attachment, maternal anxiety in the postpartum period and the interaction between these factors. Though these analyses were exploratory in nature, according to current literature, we expected infant attachment quality to be mainly predicted by interactive measures (e.g., Stevenson-Hinde et al., 2013).

## Study 1

### Materials and Methods

#### Procedures

The current secondary analyses consist of two subsamples derived from a larger longitudinal sample previously described elsewhere (Reck et al., 2013, 2018a,b; Richter and Reck, 2013; Tietz et al., 2014; Müller et al., 2015, 2016; Zietlow et al., 2019). The independent ethics committee of the medical faculty, Ruprecht-Karls-University, Heidelberg, Germany approved the study protocol prior to the first assessment. After the study procedures had been fully explained to the caregivers, we obtained written informed consent to participate in the study.

The data for the first part of the study were collected from 2006 to 2010. At 3–8 months postpartum, the caregiver–infant interaction was videotaped in laboratory during a standardized interaction experiment, namely, the Face-to-Face-Still-Face paradigm (FFSF). The FFSF was designed by Tronick et al. (1978) and in its most prevalent form (Mesman et al., 2009) consists of three episodes, i.e., the play, the still-face and the reunion episode, in which each episode lasts 120 s. Throughout the procedure, the infant is secured in a booster seat. The initial play episode is a face-to-face-interaction between the caregiver and the infant. The caregivers are instructed to play with their infants as they would at home, however, without the use of toys and/or pacifiers. At the end of the play episode, the caregivers are instructed to react to an acoustic signal by turning their head aside and silently count to ten (transition). Next, they turn their head back around but look slightly above their infant’s head, however, without engaging in any gestures, facial expressions, or vocalizations for the next 120 s (still-face). Finally, during the reunion episode, the caregivers are required to resume face-to-face-play with their infant for the last 120 s. After the FFSF, we carried out the German version of the Structured Clinical Interview for DSM-IV Disorders (Wittchen et al., 1997). Furthermore, questionnaires regarding sociodemographic and psychological variables were handed out to fill out at home. Around 1 year postpartum, the dyads were invited to revisit the lab for the strange situation (Ainsworth et al., 1978) for 12- to 24-month olds. The strange situation is designed to elicit exploration and attachment behavior in the child, and thus enable the observation and evaluation of attachment security. Like the FFSF, this procedure was videotaped. The strange situation is, like the FFSF, a standardized behavioral experiment that involves a sequence of eight episodes each lasting approximately 3 min, in which a caregiver and her child are repeatedly separated, reunited and a strange person is introduced. Attachment is classified based on the infant’s behavior. The reunion episodes (episodes 5 and 8) are coded concerning proximity seeking, contact maintaining, proximity avoidance and resistance to contact.

#### Measures

##### Maternal Mental Disorders

Mental pathology during the postpartum period was assessed via the German version of the Structured Clinical Interview for DSM-IV Axis I Disorders (SCID-I, Wittchen et al., 1997). The SCID-I was a widely used semi-structured interview for the diagnosis of selected disorders. It was the diagnostic gold standard at the time. According to the DSM-IV, anxiety disorders included generalized anxiety disorder, panic disorder with and without agoraphobia, agoraphobia without history of panic disorder, specific phobias, social phobia, obsessive-compulsive disorder, posttraumatic stress disorder, and anxiety disorder not otherwise specified.

##### Recollected Prepartum Distress

Prepartum distress was assessed retrospectively via a self-report instrument, namely the Prenatal Emotional Stress Index (PESI; Möhler et al., 2006). The PESI assesses emotional distress during pregnancy separately with 11 items per pregnancy trimester. The items assess anxiety, sadness, joy, distress, and tension via a visual analog scale ranging from 0% to 100%. The item values (2 items with reversed polarity) are averaged for each trimester, resulting in three PESI scores ranging from 0% to 100%. Measures for internal consistency were taken from the larger study sample (*N* = 111): We bootstrapped (*N* = 1.000 samples) 95% confidence intervals for McDonald’s ω (Hayes and Coutts, 2020) which revealed a good to excellent reliability (ω = [0.88;0.94] for the first, ω = [0.88;0.95] for the second and ω = [0.91;0.95] for the third trimester). We selected the PESI score for the third trimester as independent measure. Thus, we used the measure with the least memory bias.

##### Dyadic Interaction

Two trained and reliable coders coded the interactive behaviors of the infants and caregivers during the FFSF using the Noldus Observer Video-Pro coding system with 1-s time intervals. They were blinded to the hypotheses of the study and maternal diagnostic status. They used the German translation and revision of the microanalytical Infant and Caregiver Engagement Phases (ICEP-R; Reck et al., 2009). The engagement phases combine information from the face, direction of gaze and vocalizations of the infants and caregivers. For the infant, the following engagement phases can be coded: negative engagement (further divided into withdrawn and protest), object/environment engagement, social monitor, and social positive engagement. For the caregiver, the engagement phases are negative engagement (further divided into withdrawn, hostile and intrusive), non-infant focused engagement, social monitor/no vocalizations or neutral vocalizations, social monitor/positive vocalizations, and social positive engagement. 10% of the video tapes from the larger longitudinal sample (*n* = 9 of *N* = 91) were randomly selected and coded by both of the two independent study coders to assess the inter-rater reliability. The coders were unaware of which videos were used for reliability. The inter-rater reliability was determined using Cohen’s κ (Cohen, 1960). The achieved values of Cohen’s κ (κ = 0.82 for the infant codes; κ = 0.73 for the maternal codes) were similar to those reported in previous studies (Tronick et al., 2005; Reck et al., 2011). Positive social matching states were defined as the caregiver and infant simultaneously exhibiting the same affective-behavioral state as follows: the caregiver is in positive engagement or social monitor/positive vocalizations and the infant is in positive engagement or social monitor. We calculated the independent measures – the latency to interactive reparation – as the time interval from interaction onset to positive social match onset, that is, the initial mismatch duration of the respective FFSF episode in seconds. As the reunion episode is particularly informative regarding the regulatory quality of the interaction (Weinberg and Tronick, 1996), we selected the latency to interactive reparation during the reunion episode as independent measure.

##### Attachment Quality

Two trained and reliable coders annotated the videos of the strange situation paradigm. They were blinded to the hypotheses of the study and the maternal diagnostic status. Infants were classified as secure, insecure-avoidant, insecure-ambivalent or disorganized according to their behavior throughout the strange situation paradigm, and especially in the reunion episodes (see above). The disorganized category was assigned, if the attachment behavior was no longer organized or directed toward the caregiver (Simonelli and Parolin, 2017). The 25% of the video recordings from the larger longitudinal sample (*n* = 19 of *N* = 77) were randomly selected and coded by both of the two independent study coders to assess inter-rater reliability. The coders were not able to distinguish if they were coding videos for the reliability assessment or for the general study purpose. The inter-rater reliability was determined using Cohen’s κ (Cohen, 1960). The achieved values of Cohen’s κ (κ = 0.82) were similar to or higher than those reported in previous studies (Behrens et al., 2016; Smith et al., 2016). As we were interested in predicting secure vs. insecure attachment quality, we binary coded all secure patterns as “0 = secure” and all insecure and disorganized patterns as “1 = insecure/disorganized.”

#### Sample

In this project, we focused on the primary caregiver, which in most cases is the mother (e.g., Harmon and Perry, 2011). Mothers were included in the clinical group, if they were diagnosed with at least one of the following anxiety disorders according to the DSM-IV (American Psychiatric Association, 2000) in the postpartum period: panic disorder with agoraphobia, agoraphobia without history of panic disorder, generalized anxiety disorder, social phobia, obsessive compulsive disorder, posttraumatic stress disorder and anxiety disorder not otherwise specified. A specific phobia was not considered as a sufficient condition due to lowered clinical relevance. However, if the specific phobia did not occur as single diagnosis but occurred as a comorbidity to other clinically significant anxiety disorders, we did not exclude the respective cases. Mothers were excluded from the clinical group if an acute or former psychosis, a current or former bipolar disorder, current substance abuse or acute suicidal tendency was diagnosed. Despite initial screening efforts to exclude mothers with any other comorbid psychological disorder, the occurrence of comorbid disorders after screening did not exclude a mother, if it was ascertained that the comorbid disorder constituted a secondary diagnosis. Healthy controls were included if they didn’t have any current or antecedent axis I diagnosis according to the DSM-IV.

Initially, 122 mothers with their infants were recruited for the larger study. All mothers were of Caucasian ethnicity. *n* = 14 mothers were excluded due to meeting diagnostic exclusion criteria. For the first subsample, we excluded *n* = 50 cases as one of the main variables was missing: *n* = 18 interactive measures at 3–8 months and partly overlapping *n* = 37 attachment measures at 12–24 months. Consequently, for the first subsample the clinical group comprised *n* = 22 mothers with an anxiety disorder while *n* = 36 mothers were included in the control group.

#### Data Analysis

We used R (R Core Team, 2021, v. 4.1.1) in combination with RStudio^®^ (RStudio Team, 2021, v. 1.4.1717) for Microsoft Windows 10^®^ for all analyses. We used the following packages: “haven” (Wickham and Miller, 2021, v. 2.4.3), “tidyverse” (Wickham et al., 2019), “naniar” (Tierney et al., 2021, v. 0.6.1), “psych” (Revelle, 2021, v. 2.1.6), “MBESS” (Kelley, 2020, v. 4.8.0), “survival” (Therneau and Grambsch, 2000; Therneau, 2021;, v. 3.2.13).

To ascertain that list-wise case exclusions were valid for our analyses, we evaluated if missing values occurred at random. Thus, we tested the missing-completely-at-random (MCAR)-condition by carrying out Little’s MCAR test (Little, 1988) once for each subsample. Moreover, for each subsample, we evaluated the comparability between the clinical and the control group regarding sociodemographic and birth-related variables. Depending on the measurement level, we used *OR*, *U*, and *t*-tests for this analytic step. In case of significant differences and dependent on the measurement level, we Spearman- or Pearson-correlated the potential confounder with the other study variables to ascertain if it needed to be controlled for in the main analyses.

The analyses regarding the first part of the study refer to the predictive quality of maternal diagnostic status and dyadic interaction regarding infant secure vs. insecure attachment by controlling for effects of prepartum distress. Thus, we used a series of hierarchical generalized binomial regression models with logit-link-function and likelihood-ratio coefficient tests.

For solely descriptive reasons, we added a series of hierarchical Cox regressions on the dummy-coded and time-dependent event “first match”. The time variable was the latency to interactive reparation in seconds. The initial predictors were maternal diagnostic status, dummy-coded attachment quality of their infants and prepartum distress. The coefficients were tested via *z*-statistic. These retrospective analyses used attachment quality as strata and the prior assessed interactive quality as outcome. Though these analyses do not inform about the predictive quality of attachment, they inform about the interactive quality of infants later classified as securely vs. insecurely attached. Despite the Cox regressions, the hierarchical model tests started with full-factorial models including all two-way and three-way interaction terms. The hierarchical set of Cox regressions started exclusively with main effects. Terms were excluded from the models if they failed to significantly contribute to explaining the dependent variables. The procedures ended with the model that only contained significant predictors.

Regarding the binomial and the Cox regressions, the relative risks and hazard ratios, respectively, serve as estimators of effect sizes. Empirical *p*-values are reported two-tailed. The critical α-errors of the two confirmative analyses sets (i.e., Study 1: binomial regressions; Study 2: mixed models) were Holm–Bonferroni adjusted (Holm, 1979) regarding multiple testing. This sequential procedure controls the family-wise error-rate by adjusting the critical α-level for each of the individual hypotheses. Thus, the critical α is set to 0.025 for the first and 0.05 for the second model series. The α-errors were not adjusted for the descriptive Cox regression. For the full statistical procedure see the knitted R-markdown in the Supplementary Data Sheet 1. Due to the sensitive nature of the current data, it is available on request only.

### Results

#### Preliminary Analysis

For the MCAR-test we considered the following variable categories: Sociodemographic variables (e.g., maternal age), birth-related date (e.g., gestation age), questionnaire data (PESI and questionnaires not described in the current study), interaction data (ICEP-R, Reck et al., 2009) and developmental data not described in the current study (Bayles Scales of Infant and Toddler Development – III; Bayley, 2006). The MCAR-test turned out non-significant (χ^2^ = 1.352, *df* = 1.330, *p* = 0.328). Thus, we concluded that the list-wise case exclusions were valid for our analyses.

#### Sample Description

In the clinical sample (*n* = 22), *n* = 14 mothers had multiple anxiety disorders (median = 2). *n* = 8 women were diagnosed with two, *n* = 4 mothers with three and *n* = 2 women with four anxiety disorders. Overall, there were *n* = 10 mothers with a panic disorder or agoraphobia. *n* = 6 women fulfilled the criteria for a social phobia. Obsessive-compulsive disorders were diagnosed in *n* = 8 mothers, while *n* = 1 woman had a posttraumatic stress disorder. There were *n* = 12 mothers with a generalized anxiety disorder and *n* = 1 woman with an anxiety disorder not otherwise specified. *n* = 6 of the mothers were diagnosed with an additional specific phobia as a disorder comorbid to other clinically significant anxiety disorders. *n* = 16 women reported that at least one anxiety disorder had an onset already prior to pregnancy. Another *n* = 3 mothers had an onset during pregnancy and an additional *n* = 3 mothers after birth. As reported above, there were some women with comorbid disorders in our sample: *n* = 1 mother had a comorbid major depressive episode, *n* = 1 woman had a dysthymia, *n* = 1 case had a somatoform disorder and *n* = 1 mother was diagnosed with a comorbid binge eating disorder. The full sample description and tests on comparability between subgroups is reported in Tables 1A,B. There were no differences between the two subgroups (*p* > 0.14) on sociodemographic variables except for the number of children (*p* = 0.02): Mothers in the control group had more children (median = 2) than mothers in the clinical group (median = 1). However, as the Spearman-correlations (ρ < 0.17) with the study variables were non-significant (*p* > 0.21), we refrained from controlling the number of children in the models.

**TABLE 1A:** Maternal and infant parametric demographics and tests on comparability of subgroups 3–8 months postpartum (Study 1).

	Overall			Control		Anxiety		Test statistics	
	Range	*M*	*SD*	*M*	*SD*	*M*	*SD*	*| t|*	*p*
Maternal age (years)	22.0–42.0	33.1	5.2	33.4	5.4	32.6	4.9	0.57	0.57
Gestation age (weeks)	36.3–41.9	39.4	1.3	39.5	1.3	39.3	1.3	0.52	0.60
APGAR (10 min)	8.0–10.0	9.9	0.5	9.9	0.4	9.8	0.5	0.82	0.41
Infant age at FFSF (months)	2.8–7.2	3.7	1.1	3.6	1.1	3.9	1.1	1.04	0.30
Infant age at SST (months)	13.2–22.7	19.2	1.4	18.9	1.6	19.6	1.0	1.49	0.14

*t, t-value; p, empirical α-error; M, mean; SD, standard deviation; FFSF, Face-to-Face-Still-Face paradigm; SST, Strange Situation Test.*

**TABLE 1B:** Maternal and infant non-parametric demographics and tests on comparability of subgroups 3–8 months postpartum (Study 1).

	Overall		Control		Anxiety		Test statistics	
Maternal education	*n*	%	*n*	%	*n*	%	*W*	*p*
High or low secondary qualification	14	24.1	8	22.2	6	27.3	413.0	0.77
University entrance qualification	11	19.0	7	19.4	4	18.2		
University degree	33	56.9	21	58.3	12	54.5		

**Number of children**	^a^		^b^		^c^			

One infant	33	56.9	16	44.4	17	77.3	529.0	0.02
Two infants	19	32.8	15	41.7	4	18.2		
Three or more infants	6	10.3	5	13.9	1	4.5		

**Marital status**							***OR* 95% *CI***	***p***

Married	40	72.7	26	76.5	14	66.7	0.62 [0.16;2.47]	0.54
Not married	15	27.3	8	23.5	7	33.3		

**Infant sex**								

Female infants	34	58.6	19	52.8	15	68.2	0.53 [0.14;1.79]	0.28
Male infants	24	41.4	17	47.2	7	31.8		

*^a^Median = 1.*

*^b^Median = 2.*

*^c^Median = 1.*

*Valid %, percentage of valid values; W, statistical value of Wilcoxon test for independent samples (U test); p, empirical α-error; OR, odds ratio; 95% CI, 95% confidence interval of test statistic.*

#### Descriptive Statistics of Study Variables

In our sample, *n* = 37 infants were classified as securely attached while *n* = 13 infants were insecure-avoidant, *n* = 1 infant insecure-ambivalent and *n* = 7 infants were disorganized. Thus, the insecurely attached group comprised *n* = 21 infants. In mean, it took dyads 8.9 s to achieve a match during the reunion episode (*SD* = 20.0 s) ranging between 0 and 109.2 s. *n* = 9 dyads did not achieve a match at all, thus decreasing the list-wise *n* in models with the raw latency to reparation as a predictor.

Regarding distress during pregnancy, the correlations between the first and second trimester (*r* = 0.89, *p* < 0.01, 95% CI = [0.82; 0.94]), between the second and third trimester (*r* = 0.85, *p* < 0.01, 95% CI = [0.75; 0.91]) and between the first and third trimester (*r* = 0.69, *p* < 0.01, 95% CI = [0.52; 0.81]) revealed a medium-to-high inter-scale consistency, thus supporting the choice to concentrate on only one of these measures. Our sample reached an overall PESI-mean during the third trimester of pregnancy of *M* = 30.2% (*SD* = 24.3%), ranging between 0% and 85.5%. *n* = 3 women did not fill out the questionnaires. However, as the distress during pregnancy was not our primary predictor, we did not generally exclude these dyads. Notably, the list-wise *n* varies depending on the inclusion of the PESI-score in the models.

#### Main Analysis

The series of hierarchical generalized logistic regressions on secure vs. insecure attachment revealed a final model (AIC = 55.947) consisting only of two predictors, i.e., maternal diagnostic status and latency to interactive reparation. All other predictors (i.e., prepartum distress and all interaction terms) were stepwise eliminated as they did not significantly contribute to the model (*p* > 0.025, for details see Table 2 comparing the first and the final model of the series as well as the Supplementary Table 1 demonstrating the excluded models 2–5). Maternal anxiety disorders were revealed as strong predictors of insecure attachment: With an odds ratio of OR = 5.446 (*p* = 0.010), they increased the risk for insecure attachment by more than fivefold. However, latency to reparation seems to add to the effect of diagnostic category: With an OR = 1.042, this predictor increases the risk for insecure attachment by 4.2% for each passing second (*p* = 0.022).

**TABLE 2:** First and final generalized binomial regression models on infant attachment out of hierarchical backward procedure.

	Model 1				Final model			
Predictors	*OR*	95% *CI OR* lower bound	95% *CI OR* upper bound	*p*	*OR*	95% *CI OR* lower bound	95% *CI OR* upper bound	*p*
Intercept	0.075	0.003	1.050	*/*	0.161	0.050	0.411	*/*
Anxiety disorder	0.378	0.000	65.418	0.021	5.446	1.437	23.166	0.010
Interactive reparation	0.817	0.425	1.150	0.029	1.042	1.005	1.110	0.022
Prepartum distress	1.034	0.888	1.186	0.039	/	/	/	/
Anxiety disorder * interactive reparation	1.637	0.895	5.031	0.204	/	/	/	/
Anxiety disorder * prepartum distress	1.019	0.873	1.216	0.442	/	/	/	/
Interactive reparation * prepartum distress	1.015	0.993	1.056	0.853	/	/	/	/
Anxiety disorder * interactive reparation * prepartum distress	0.982	0.943	1.007	0.164	/	/	/	/

*Coding of attachment outcome: 0 = secure, 1 = insecure.*

*OR, odds ratio; CI, confidence interval; p, empirical α-error.*

*Model 1: AIC = 53.962, fitted probabilities numerically 0 or 1 occurred.*

*Final model: AIC = 55.947.*

**FIGURE 1:**
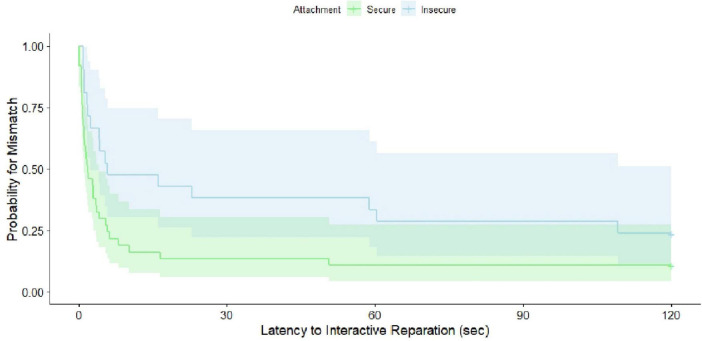
Survival plot on time-dependent match stratified by later secure vs. insecure attachment quality.

For the series of descriptive hierarchical Cox regressions on the time-dependent event “match,” we created a dummy-coded variable “match” whereas “1” was coded for “match achieved during FFSF-interaction” and “0” was coded for “no match achieved during FFSF-interaction”. Moreover, we recoded the raw values of latency to interactive reparation in two ways: (1) We coded the measure to 1 s, if dyads already started with a match in the interaction, not to lose these specific dyads in the analyses. (2) We coded the measure to 120 s (the maximum observation period) for all dyads not achieving any match during the early interaction to integrate them as censored data in the analysis. The final model (*LR* = 4.9, *df* = 1, *p* = 0.03) only consisted of one factor: i.e., infant attachment quality (see Figure 1). The other two factors, i.e., maternal diagnostic status and prepartum distress were stepwise eliminated as they did not significantly contribute to the model (*p* > 0.22, for details see the Supplementary Table 2). With a hazard ratio of *HR* = 0.52 (95% CI = [0.28; 0.94]; *p* = 0.03), attachment quality was revealed as a strong factor, meaning that at 12–24 months insecurely attached infants were 48% less likely to having achieved a match in the interaction 3–8 months postpartum.

## Study 2

### Materials and Methods

#### Procedures

The data for the second part of the study 5–6 years postpartum was collected from 2010 to 2014. During a lab visit, child cortisol-reactivity was assessed via salivary samples taken immediately before, 20 and 40 min after a socioemotional stressor: engagement with unfamiliar peers and a clown. Two caregiver–child pairs from the same study, who did not know each other, were asked to enter an empty room with a carpet and two chairs located in each corner opposite the other. Pairs were chosen to have children of the same gender. The caregivers were asked to sit on the chairs and fill in questionnaires while the children were invited to sit in the middle of the room and play on a carpet with some gender appropriate toys located there. After a few minutes an attractive toy was placed in the middle of the carpet, and it was coded who grabbed it first. Then, a clown entered the room, told a story, and invited the children to play with him. The whole procedure lasted 20 min. The cortisol baseline was assessed on two consecutive days at home.

#### Measures

##### Salivary Cortisol

For the assessment of salivary cortisol, children sucked on a cotton ball until it was saturated. The saliva was then expressed and stored at −20°C until analysis. To account for possible effects of circadian rhythm on cortisol reactivity, we attempted to have the visits to the laboratory, as well as the baseline assessments at home always at around the same time of the day. Moreover, since cortisol reactivity is strongly associated with daytime napping or feeding, the caregivers were instructed to keep their children well rested and well fed on their usual routine in order not to confound the cortisol assessment. The baseline measures were averaged over both assessments. Sampling, storage, transport and analysis of cortisol samples took place according to standard protocols (Schwartz et al., 1998). The limit of detection of the used assay was 0.15 ng/ml. Intra-assay variances were 5.95% volume for 2.6 μg/100 ml, 1.59% for 17 μg/100 ml and 4.62% for 26.6 μg/100 ml.

#### Sample

Besides the dyads excluded due to diagnostic criteria or missing attachment measures (*n* = 51), in the second subsample, we lost further *n* = 23 dyads missing the follow-up at 5–6 years postpartum. Additionally, *n* = 9 children had missing cortisol values during the stress paradigm (*n* = 7) or at baseline (*n* = 9). Thus, for the second subsample, *n* = 26 mothers were included in the control group, while the clinical group comprised *n* = 13 mothers.

#### Data Analysis

We used the following packages: “ggplot2” (Wickham, 2016), “survminer” (Kassambara et al., 2021, v. 0.4.9), “lme4” (Bates et al., 2015), “lmerTest” (Kuznetsova et al., 2017) and “writexl (Ooms, 2021, v. 1.4.0).”

The analyses regarding the second part of the study refer to the predictive quality of the dummy-coded attachment quality and the maternal diagnostic status during the postpartum period on child cortisol-reactivity by controlling for cortisol baseline. Thus, we used a series of hierarchical mixed models on the three nested cortisol measures during the socioemotional stressor. The main effects were tested via *F*-statistic. The hierarchical model tests started with full-factorial models including all two-way and three-way interaction terms. Terms were excluded from the models if they failed to significantly contribute to explaining the dependent variables. The procedures ended with the model that only contained significant predictors.

Regarding the mixed models effect sizes are reported as partial ω^2^, which is a population-based estimator of explained variance. Empirical *p*-values are reported two-tailed. The critical α-errors of the two confirmative analyses sets (i.e., Study 1: binomial regressions; Study 2: mixed models) were Holm Bonferroni adjusted (Holm, 1979). This sequential procedure controls the family-wise error-rate by adjusting the critical α-level for each of the individual hypotheses. Thus, the critical α is set to 0.025 for the first and 0.05 for the second model series.

### Results

#### Preliminary Analysis

For the MCAR-test, we considered the following variable categories: sociodemographic variables (e.g., maternal age), birth-related date (e.g., gestation age), questionnaire data (PESI and questionnaires not described in the current study (e.g., the Child Behavior Checklist, Arbeitsgruppe-Deutsche-Child-Behavior-Checklist, 2002), interaction data (Coding Interactive behavior, Feldman, 1998), cortisol data and developmental data not described in the current study (Kaufman Assessment Battery for Children; Melchers and Preuß, 2009). The MCAR-test turned out non-significant (χ^2^ = 1.188, *df* = 1.132, *p* = 0.119). Thus, we concluded that the list-wise case exclusions were valid for our analyses.

#### Sample Description

In our clinical sample (*n* = 13), *n* = 8 mothers had multiple anxiety disorders (median = 2) during the postpartum period. *n* = 4 women were diagnosed with two, *n* = 3 mothers with three and *n* = 1 woman with four anxiety disorders. Overall, there were *n* = 6 mothers with a panic disorder or agoraphobia. *n* = 4 women fulfilled the criteria for a social phobia. Obsessive-compulsive disorders were diagnosed in *n* = 6 mothers, while *n* = 1 woman had a posttraumatic stress disorder. There were *n* = 6 mothers with a generalized anxiety disorder and *n* = 1 woman with an anxiety disorder not otherwise specified. *n* = 2 of the mothers were diagnosed with an additional specific phobia. *n* = 10 women reported that at least one anxiety disorder had an onset already prior to pregnancy. Another *n* = 1 mother had an onset during pregnancy and an additional *n* = 2 mothers after birth. As reported above, there were some women with comorbid disorders in our sample: *n* = 1 mother had a comorbid major depressive episode, *n* = 1 woman had a dysthymia and *n* = 1 mother was diagnosed with a comorbid binge eating disorder. For the follow-up sample, the mother with the somatoform disorder was lost. *n* = 7 mothers still suffered from an anxiety disorder 5–6 years postpartum. The full sample description and tests on comparability between subgroups is reported in Tables 3A,B. There were no differences between the two subgroups (*p* > 0.09) except for marital status (*p* < 0.01): Mothers in the control group were more frequently married than mothers in the clinical group. However, as the Spearman correlations (*r* < 0.16) with the study variables were non-significant (*p* > 0.36), we refrained from controlling marital status.

**TABLE 3A:** Maternal and infant parametric demographics and tests on comparability of subgroups 5–6 years postpartum (Study 2).

	Overall			Control		Anxiety		Test statistics	
	Range	*M*	*SD*	*M*	*SD*	*M*	*SD*	*| t|*	*p*
Maternal age (years)	27.0–48.0	39.8	5.3	40.0	5.0	39.2	5.9	0.45	0.66
Infant age at SST (months)	13.2–22.5	19.2	1.6	18.9	1.9	19.6	1.0	1.18	0.25
Child age at follow-up (years)	5.1–6.5	5.7	0.4	5.7	0.4	5.8	0.3	0.89	0.38

*t, t-value; p, empirical α-error; M, mean; SD, standard deviation; SST, Strange Situation Test.*

**TABLE 3B:** Maternal and infant non-parametric demographics and tests on comparability of subgroups 5–6 years postpartum (Study 2).

	Overall		Control		Anxiety		Test statistics	
Maternal education	*n*	%	*n*	%	*n*	%	*W*	*p*
High or low secondary qualification	9	23.1	6	23.1	3	23.1	187.5	0.55
University entrance qualification	8	20.5	4	15.4	4	30.8		
University degree	22	56.4	16	61.5	6	46.2		

**Number of children**	^a^		^b^		^c^			

One child	7	20.0	4	16.0	3	30.0	221.5	0.09
Two children	16	45.7	11	44.0	5	50.0		
Three or more children	12	34.3	10	40.0	2	20.0		

**Marital status**							***OR* 95% *CI***	***p***

Married	31	88.6	25	100.0	6	60.0	0.00 [0.00; 0.49]	<0.01
Not married	4	11.4	0	0.0	4	40.0		

**Infant sex**								

Female infants	26	66.7	15	57.7	11	84.6	0.26 [0.02;1.55]	0.15
Male infants	13	33.3	11	42.3	2	15.4		

*^a^Median = 2.*

*^b^Median = 2.*

*^c^Median = 2.*

*W, statistical value of Wilcoxon test for independent samples (U test); p, empirical α-error; OR, odds ratio; 95% CI, 95% confidence interval of test statistic.*

#### Descriptive Statistics of Study Variables

In our sample, *n* = 28 infants were classified as securely attached, while *n* = 8 infants were insecure-avoidant and *n* = 3 infants were disorganized. Thus, the insecurely attached group comprised *n* = 11 infants. The descriptive statistics for cortisol measures are demonstrated in Table 4. In mean, the samples were taken around 2 pm (*M* = 13.9) with a standard deviation of *SD* = 1.8 h and ranging from 9 am to around 4 pm. Moreover, the baseline was taken in mean at 7:30 pm (*M* = 19.5) with a standard deviation of *SD* = 0.9 h and ranging from 5 pm to 8:45 pm. Due to the high range of sample times, we checked associations to the cortisol measures. However, all correlations (*r* < 0.17) were non-significant (*p* > 0.30). Thus, we refrained from controlling for time of day.

**TABLE 4:** Descriptive statistics of cortisol measures in ng/ml.

	*M*	*SD*	*SE*	*Min*	*Max*
Measure immediately before stressor	1.45	0.91	0.15	0.47	5.40
Measure + 20 min after stressor	1.28	0.80	0.13	0.44	4.25
Measure + 40 min after stressor	1.03	0.47	0.07	0.26	2.45
Baseline 1	0.80	1.13	0.18	0.12	5.72
Baseline 2	0.58	0.40	0.06	0.17	2.16
Mean baseline	0.69	0.69	0.11	0.18	3.63

*M, mean; SD, standard deviation; SE, standard error; Min, minimal value; Max, maximum value.*

#### Main Analysis

**FIGURE 2:**
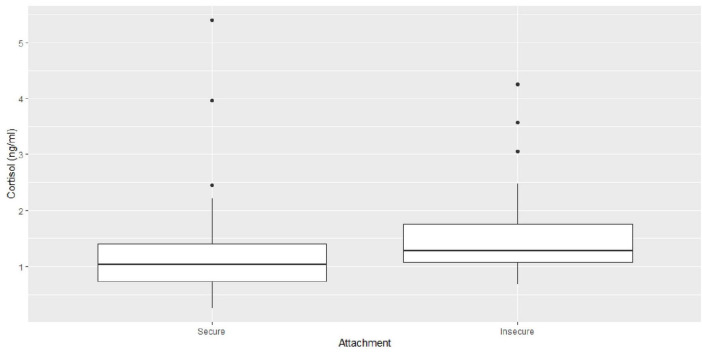
Box-plots regarding the main effect of attachment quality on cortisol measures.

**FIGURE 3:**
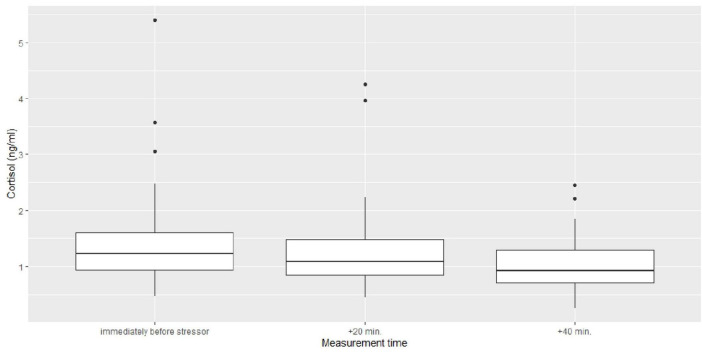
Box-plots regarding the main effect of time on cortisol measures.

The series of hierarchical mixed models on cortisol measures revealed a final model (*REML* = 233.1) with three predictors: time, cortisol baseline and attachment quality. All other predictors, maternal diagnostic status during the postpartum period and all interaction terms were stepwise eliminated as they did not significantly contribute to the model (*p* > 0.24, for details see the Supplementary Table 3). The inferential statistics are demonstrated in Table 5. The descriptive statistics of the main effects of attachment quality and time are depicted in Figures 2, 3 as well as in Table 6. The cortisol levels of children with an insecure attachment quality at 12–24 months were higher during the stress paradigm at the age of 5 to 6 years compared to the ones of securely attached children. This effect explains around 4% of variance in cortisol measures (ω^2^ = 0.04), while time explained about 8% of variance (ω^2^ = 0.08). Still, most of the variance is explained by cortisol baseline with about 9% (ω^2^ = 0.09).

**TABLE 5:** Mixed model on cortisol measures.

	Sum of squares	Mean squares	Numerator *df*	Denominator *df*	*F*	*p*
Attachment quality	1.685	1.685	1	36	5.916	0.020
Time	3.349	1.674	2	76	5.878	0.004
Cortisol baseline	3.432	3.432	1	36	12.047	0.001

*Df, degrees of freedom; F, F-statistic; p, empirical α-error.*

**TABLE 6:** Descriptive statistics on main effects of attachment and time on cortisol measures in ng/ml.

Attachment	*M*	*SD*	*SE*	*Min*	*Max*
Secure	1.15	0.72	0.08	0.26	5.40
Insecure	1.52	0.81	0.14	0.68	4.25
**Time**					
Measure 1	1.45	0.91	0.15	0.47	5.40
Measure 2	1.28	0.80	0.13	0.44	4.25
Measure 3	1.03	0.47	0.07	0.26	2.45

*M, mean; SD, standard deviation; SE, standard error; Min, minimal value; Max, maximum value.*

## Discussion

The present study aimed at testing the hypotheses that prepartum distress, maternal anxiety disorders in the postpartum period as well as latency to reparation predict infant secure vs. insecure attachment and possibly moderate each other’s effect. To the best of the authors’ knowledge, there is only one other study to date that has demonstrated associations between maternal anxiety, interactional behavior and insecure attachment in a sample of 4.5 year-olds and their mothers (*N* = 98, Stevenson-Hinde et al., 2013). These results showed that maternal anxiety was a significant predictor of maternal sensitivity measures which in turn predicted attachment security. Compared to these macro-temporal analyses of interaction behaviors, our perspective is that while maternal sensitivity may be an important predictor of attachment, in particular micro-temporal processes such as latency to reparation may represent critical key mechanisms in this context (compare Mesman, 2010). Maternal sensitivity is a macro-temporal measure in which the entire interaction is judged to be sensitive or insensitive on a rank ordered scale. Thus, it is likely that one misses to register the actual details of mother–infant engagement just in time which could lead to secure or insecure attachment quality. So far, there are only a few studies that have focused on micro-temporal processes (e.g., Beebe et al., 2010); the vast majority of studies use global rating systems to analyze interactive paradigms in this context (e.g., Stevenson-Hinde et al., 2013). Of course, micro- and macro-temporal parameters may be interrelated – thus, we assume that in dyads with sensitively interacting mothers, interactive reparation also succeeds more often (Noe, 2008). However, it is our perspective, that especially the micro-temporal mechanisms may hold the key to understanding the dynamic nature of these multifaceted processes (compare Provenzi et al., 2018). In our study, we examine the micro-temporal process of interactive reparation as a possible interactive mechanism underlying security of attachment. Using regression analysis on a set of possible predictors, as expected we found that apart from maternal diagnostic status, latency to reparation was the only other significant predictor for attachment insecurity.

To predict infant attachment security, not only maternal sensitivity but also infant affect regulation plays an important role. Braungart-Rieker et al. (2001), for example, were able to show that both aspects discriminate between secure and insecure infants. As proposed by DiCorcia and Tronick (2011), interactive reparation – the mutual adaptive process of the dyad oscillating between coordinated and uncoordinated states – seems to shape not only infant attachment security but also – more fundamentally – infant regulatory strategies. Also other studies show that for dyadic co-regulation, sensitive reactions of the caregivers are crucial not only for healthy development (e.g., Malmberg et al., 2016) but likewise for behavioral and physiological reactions (e.g., Haley and Stansbury, 2003; Conradt and Ablow, 2010). As maternal sensitivity is of great importance for infant attachment security, infants of insensitive mothers might frequently lack sufficient regulatory scaffolding with possible long-term consequences for child development (Leclère et al., 2014). And it is our understanding that this regulatory scaffolding is essential in the development of emotion regulation, and thus a secure attachment quality (Kerns and Brumariu, 2014). This is also in line with results of Beebe et al. (2010) showing that very high or very low interactive contingency was linked to insecure attachment in infancy in a sample of anxious mothers. Contingency can be interpreted as a measure of matched states and points toward the same direction as our results. Thus, our findings highlight the importance of specific micro-interactional patterns of mother–child interaction for infants’ regulation (Müller et al., 2015) and the development of a secure attachment quality. Consequently, the interactive dysregulation could be partly responsible for the increased risk of developing mental disorders later in life (compare Verhees et al., 2021). Nevertheless, child emotion regulation and their later psychopathological development was not assessed in the current study. Future projects should focus these factors when investigating developmental dependencies between early interactive patterns and child attachment.

Furthermore, our results also showed that infants of mothers with postpartum anxiety disorders have a more than fivefold increased risk of developing an insecure attachment than the infants of the control group. This is in line with previous studies indicating higher attachment insecurity in children of anxious mothers (Stevenson-Hinde et al., 2011). Concerning the mechanism of transmission, e.g., for social anxiety it was demonstrated that particular this disorder goes along with insecure attachment patterns. Consequently, attachment patterns are often transmitted from mother to offspring (for review, see Martins and Gaffan, 2000) by verbal and non-verbal interactions (Ward and Carlson, 1995; Meins et al., 2001). However, in this study we did not assess maternal attachment patterns. Future studies should consider this mediating factor when investigating the development of infant attachment quality. Notably, the effect of anxiety disorder was independent of prepartum distress which turned out not to predict infant attachment quality in our data. This was somewhat surprising given the established effects of fetal programming (van den Bergh et al., 2017), however, this may be due to the fact that in our study prepartum distress was assessed retrospectively and via self-report and not via biological measures such as salivary cortisol. Future studies should consider controlling for prepartum distress via more reliable and objective measures. Moreover, the effect of maternal disorder was not moderated by our dyadic interaction measure as observed in other studies (e.g., Grant et al., 2010a,b). However, it is possible that this is due to the micro-temporal nature of our measurement: This measure may be more sensitive to influences that escape the detection threshold of macro-temporal scales. Thus, it may represent a more direct measure of spontaneous behavior as compared to parental sensitivity measures. Possibly, the behavioral quality we observe here fits better as mediating variable in the association between maternal disorder and infant attachment. Additionally, the power to detect moderation effects may have run too low in our models. Besides increased sample sizes, future studies should investigate the idea of mediation pathways in this context as the work of Stevenson-Hinde et al. (2013) suggests for macro-temporal measures.

In a second part, this study aimed at evaluating possible links between infant attachment quality as well as maternal anxiety disorders in the postpartum period and stress reactivity at preschool age. The results showed increased cortisol levels in insecurely attached children during a stress paradigm compared to securely attached children. This finding is consistent with other studies that have shown associations between attachment security and cortisol reactivity throughout life (Bernard and Dozier, 2010; Oskis et al., 2011; Pierrehumbert et al., 2012; Kidd et al., 2013). However, it is important to emphasize here that the relationship between attachment security and cortisol reactivity in preschool age is moderated by a wide variety of factors, e.g., maternal psychopathology. The study by Luijk et al. (2010), for instance, showed that the association between insecure attachment and cortisol reactivity is stronger in children of depressed mothers. In our study we did not find a significant interaction effect between maternal diagnostic status and attachment on cortisol reactivity in preschool aged children. One reason could be, that our clinical sample consisted of women with various and different anxiety disorders. Hence, it remains unclear whether disorder-specific effects accounted for this null finding. Future studies should consider focusing on more homogenous clinical samples. Another reason could be that we missed to observe a cortisol peak due to too short observational intervals or an ineffective stress paradigm. However, as Gunnar et al. (2009) point out, on average, psychological stress paradigms do not generally induce a cortisol reactivity in developmental studies. Thus, a decrease in cortisol means is a frequent result in infant and child studies (Gunnar et al., 2009; Jansen et al., 2010a). It must be noted that the lack of observable mean cortisol peaks does not imply that the analysis of respective cortisol values is useless. Rather, it has been argued that their analysis may uncover potential risk factors that account for individual differences and may adversely affect developmental trajectories. Our study suggests that one of these risk factors is represented by infant insecure attachment.

### Limitations

First, besides a rather small sample size, and thus low statistical power especially at the 5-6-year follow-up, mothers with different and multiple anxiety disorders were included in our clinical sample. However, the sample size did not allow subgroup analyses on disorder-specific effects. Moreover, according to the DSM-5 (Falkei and Wittchen, 2015), obsessive compulsive disorders are no longer classified as anxiety disorders. Therefore, special attention needs to be paid to these disorders with regard to the outcome variables in future research. Moreover, in respect to the small sample size, the analyses are rather complex. Thus, results should be regarded with cation and focused for replication attempts in future studies. Second, our sample is characterized by an overproportion of academic degrees, whereby our data is not representative for the overall population. Consequently, besides the occurrence of anxiety disorders or not, the sample comprises families with rather low risk-constellations. Third, infant salivary cortisol was assessed prior to, immediately after and 20 min after the stress paradigm. Due to few samples or the limited time frame, it is possible that we missed the cortisol peak. Fourth, it is important to mention the limited control of effects by meantime events between the measurements as well as by the wide age ranges of the infants in both the interaction and attachment assessments. Last, as the study design was observational, causality assumptions are not appropriate.

## Conclusion

Taken together, our empirical results emphasize the importance to further investigate early interactional micro-temporal markers for infant and child development. Our results underline that latency to reparation is linked to infant attachment security and this in turn influences the child‘s stress reactivity up to preschool age. During interactions, infants experience that their success or failure in repairing mismatches affects the meanings they make about themselves in the world in relation to others and to themselves (Beeghly et al., 2011): Successful reparation leads to a sense of self as effective and a sense that we – my interactional partner and me – can overcome mismatches or failures and the certainty of being able to trust the other person. Unsuccessful reparation leads to a sense of failure and a distrust of the partner. And it is this sense of trust or distrust that leads to secure or insecure attachment. Therefore, early intervention and prevention programs may be of vital importance. Our results point toward the direction that, in addition to the treatment of clinical symptoms in parents, a promising approach might be to focus on the flexibility of interactional patterns, which is represented by latency to reparation, instead of just positive interaction patterns. As the process of interactive reparation occurs in a clearly detectable time range (seconds; see also Weinberg et al., 2006; Weinberg et al., 2008) video interventions (Reck et al., 2004; Downing et al., 2014) may turn out as useful tools for increasing the flexibility in the flow of dyadic interplay between mismatching and positive matching states. The results suggest, this might improve attachment security in infancy and children’s regulatory capacities and mental health in the longer-term (Beatson and Taryan, 2003).

## Data Availability Statement

The anonymized raw data supporting the conclusions of this article will be made available by the authors, without undue reservation.

## Ethics Statement

The studies involving human participants were reviewed and approved by Medical Faculty of the Ruprecht-Karls-University, Heidelberg, Germany. Written informed consent to participate in this study was provided by the participants or the participants’ legal guardian/next of kin respectively.

## Author Contributions

MM contributed to the conceptualization, methodology, formal analysis, writing – original draft, review and editing, and visualization. A-LZ contributed to the writing – original draft, review, and editing. NK contributed to the attachment coding and writing – review and editing. CW and NN contributed to the writing – review and editing. ET contributed to the supervision and writing – review and editing. CR contributed to the investigation, project administration, supervision, and writing – review and editing. All authors contributed to the article and approved the submitted version.

## Conflict of Interest

The authors declare that the research was conducted in the absence of any commercial or financial relationships that could be construed as a potential conflict of interest.

## Publisher’s Note

All claims expressed in this article are solely those of the authors and do not necessarily represent those of their affiliated organizations, or those of the publisher, the editors and the reviewers. Any product that may be evaluated in this article, or claim that may be made by its manufacturer, is not guaranteed or endorsed by the publisher.
