# Supplementary material for: From Early Micro-Temporal Interaction Patterns to Child Cortisol Levels: Toward the Role of Interactive Reparation and Infant Attachment in a Longitudinal Study
Source: Front Psychol. 2022 Jan 20;12:807157. doi: 10.3389/fpsyg.2021.807157 (PMC8810635; doi:10.3389/fpsyg.2021.807157)
Supplement: Supplementary file 1 [file Data_Sheet_1.pdf]

Script_Repair_Attachment

Dr. Mitho Müller

Reading data

```
data <- read_spss("data/Angststudie_Datensatz_FST_MM.sav")
View(data)
```

Labeling

File failed to load: file:///C:/Users/mitho/Dokumente/B%C3%BCro/DFG-Angst/Paper%20Nathania/Repair_Attachment/manuscript/Review%

```
data$diagnosis <- factor(data$diagnosis, labels = c("Control", "Clinical"))
data$maternal_education <- factor(data$maternal_education,
                                      labels = c("Low secondary qualification",
                                                 "High secondary qualification",
                                                 "University entrance qualificatio
n",
                                                 "University degree"))
data$panic_disorder_agoraphobia <- factor(data$panic_disorder_agoraphobia,
                                              labels = c("False", "True"))
data$sociophobia <- factor(data$sociophobia,
                               labels = c("False", "True"))
data$specific_phobia <- factor(data$specific_phobia,
                                   labels = c("False", "True"))
data$obsessive_compulsive_disorder <- factor(data$obsessive_compulsive_disorder,
                                                 labels = c("False", "True"))
data$posttraumatic_stress_disorder <- factor(data$posttraumatic_stress_disorder,
                                                 labels = c("False", "True"))
data$generalized_anxiety_disorder <- factor(data$generalized_anxiety_disorder,
                                                labels = c("False", "True"))
data$anxiety_disorder_nos <- factor(data$anxiety_disorder_nos, labels = c("False",
"True"))
data$panic_disorder_agoraphobia_onset <- factor(data$panic_disorder_agoraphobia_ons
et,
                                                    labels = c("Pre", "Peri", "Pos
t"))
data$sociophobia_onset <- factor(data$sociophobia_onset, labels = c("Pre"))
data$specific_phobia_onset <- factor(data$specific_phobia_onset, labels = c("Pre"))
data$obsessive_compulsive_disorder_onset <- factor(data$obsessive_compulsive_disord
er_onset,
                                                       labels = c("Pre", "Peri", "P
ost"))
data$generalized_anxiety_disorder_onset <- factor(data$generalized_anxiety_disorder
_onset,
                                                      labels = c("Pre", "Peri", "Po
st"))
data$anxiety_disorder_nos_onset <- factor(data$anxiety_disorder_nos_onset, labels =
c("Peri", "Post"))
data$anxiety_disorders_early_onset <- factor(data$anxiety_disorders_early_onset,
                                                 labels = c("Pre", "Peri", "Post"))
data$inpatients <- factor(data$inpatients, labels = c("False", "True"))
data$social_marriage <- factor(data$social_marriage, labels = c("False", "True"))
data$birth_PDA_epidural <- factor(data$birth_PDA_epidural, labels = c("False", "Tru
e"))
data$birth_mode <- factor(data$birth_mode,
                              labels = c("Spontaneous",
                                         "Primary sectio",
                                         "Secondary sectio"))
data$infant_sex_index_infant <- factor(data$infant_sex_index_infant,
                                           labels = c("Female", "Male"))
data$match_event <- factor(data$match_event, labels = c("False", "True"))
data$FST_numb <- factor(data$FST_numb, labels = c("Secure", "Insecure"))
data$SKID_Agora_Panik_aktuell <- factor(data$SKID_Agora_Panik_aktuell, labels = c("
False", "True"))
data$Skid_Soziale_Phobie_aktuell <- factor(data$Skid_Soziale_Phobie_aktuell,
```

File failed to load: file:///C:/Users/mitho/Dokumente/B%C3%BCro/DFG-Angst/Paper%20Nathania/Repair_Attachment/manuscript/Review%

```
                                           labels = c("False", "True"))
data$Skid_Spezifische_Phobie_aktuell <- factor(data$Skid_Spezifische_Phobie_aktuel
l,
                                               labels = c("False", "True"))
data$Skid_Zwangstörung_aktuell <- factor(data$Skid_Zwangstörung_aktuell, labels = c
("False", "True"))
data$Skid_PTSD_aktuell <- factor(data$Skid_PTSD_aktuell, labels = c("False"))
data$Skid_GAS_aktuell <- factor(data$Skid_GAS_aktuell, labels = c("False", "True"))
data$Skid_ANNB_aktuell <- factor(data$Skid_ANNB_aktuell, labels = c("False"))
data$Berufstätigkeit_aktuell <- factor(data$Berufstätigkeit_aktuell, labels = c("Fa
lse", "True"))
data$Verheiratet_aktuell <- factor(data$Verheiratet_aktuell, labels = c("False", "T
rue"))
```

Function definitions for descriptive parameters

```
vnc <- function(x) {length(x)- sum(length(which(is.na(x))))}
vm  <- function(x) {mean(x, na.rm =TRUE)}
vsd <- function(x) {sd(x, na.rm = TRUE)}
vse <- function(x) {sd(x, na.rm = TRUE) / sqrt(length(x)- sum(length(which(is.na
(x)))))}
vmin <- function(x) {min(x, na.rm = TRUE)}
vmax <- function(x) {max(x, na.rm = TRUE)}
```

Study 1 - Prediction of attachment

Select variables for MCAR-test

File failed to load: file:///C:/Users/mitho/Dokumente/B%C3%BCro/DFG-Angst/Paper%20Nathania/Repair_Attachment/manuscript/Review%

```
data %>%
  select(count_anxiety_diagnoses,
         maternal_age,
         maternal_education,
         gestation_age,
         infant_age_ICEP,
         amount_infants,
         APGAR_10,
         STAIT_A1,
         STAIS_A1,
         PBQ_16_10,
         LMSCS_A1,
         IBQ_dtn_A1,
         MIB_A1,
         EPDS_A1,
         PESI_3rd_trimenon_cor,
         duration_reunion,
         CPOS_e_CPVC_e_IPOS_INEU_latency_P3,
         bIII_cognitive_composite_score,
         bIII_language_composite_score,
         bIII_motor_composite_score,
         infant_age_A4,
         STAIT_A4,
         STAIS_A4,
         MIB_A4,
         ) -> data_red
View(data_red)
```

MCAR-test

```
mcar_test(data_red)
```

```
## # A tibble: 1 x 4
##   statistic    df p.value missing.patterns
##       <dbl> <dbl>   <dbl>            <int>
## 1     1352.  1330   0.328               78
```

Exclude cases with missing values

```
data_sub <- data %>%
  filter(is.na(c(FST_numb))==FALSE) %>%
  filter(is.na(c(match_latency))==FALSE)
View(data_sub)
```

Descriptives and tests on comparability

Parametric variables

File failed to load: file:///C:/Users/mitho/Dokumente/B%C3%BCro/DFG-Angst/Paper%20Nathania/Repair_Attachment/manuscript/Review%

```
# general means
desc_par_g <- data_sub %>%
   summarise(across(c(maternal_age,
                      gestation_age,
                      infant_age_ICEP,
                      APGAR_1,
                      APGAR_5,
                      APGAR_10,
                      infant_age_A4),
                    list(mean = vm, sd = vsd, se = vse, min = vmin, max = vmax, n =
vnc)))
View(desc_par_g)
write_xlsx(desc_par_g, "desc_par_g.xlsx")

# group-specific means
desc_par_s <- data_sub %>%
   group_by(diagnosis) %>%
   summarise(across(c(maternal_age,
                      gestation_age,
                      infant_age_ICEP,
                      APGAR_1,
                      APGAR_5,
                      APGAR_10,
                      infant_age_A4),
                    list(mean = vm, sd = vsd, se = vse, min = vmin, max = vmax, n =
vnc)))
View(desc_par_s)
write_xlsx(desc_par_s, "desc_par_s.xlsx")

# tests on comparability
data_sub_con       <- subset(data_sub, data_sub$diagnosis == "Control")
data_sub_anx       <- subset(data_sub, data_sub$diagnosis == "Clinical")
t.test(data_sub_con$maternal_age,data_sub_anx$maternal_age, alternative = "two.side
d", var.equal=TRUE, conf.level = 0.95)
```

```
##
##  Two Sample t-test
##
## data:  data_sub_con$maternal_age and data_sub_anx$maternal_age
## t = 0.56639, df = 56, p-value = 0.5734
## alternative hypothesis: true difference in means is not equal to 0
## 95 percent confidence interval:
##  -2.024354  3.620313
## sample estimates:
## mean of x mean of y
##  33.38889  32.59091
```

```
t.test(data_sub_con$gestation_age,data_sub_anx$gestation_age, alternative = "two.si
ded", var.equal=TRUE, conf.level = 0.95)
```

File failed to load: file:///C:/Users/mitho/Dokumente/B%C3%BCro/DFG-Angst/Paper%20Nathania/Repair_Attachment/manuscript/Review%

```
##
##  Two Sample t-test
##
## data:  data_sub_con$gestation_age and data_sub_anx$gestation_age
## t = 0.52155, df = 53, p-value = 0.6042
## alternative hypothesis: true difference in means is not equal to 0
## 95 percent confidence interval:
##  -0.5471731  0.9317269
## sample estimates:
## mean of x mean of y
##  39.49160  39.29932
```

```
t.test(data_sub_con$infant_age_ICEP,data_sub_anx$infant_age_ICEP, alternative = "tw
o.sided", var.equal=TRUE, conf.level = 0.95)
```

```
##
##  Two Sample t-test
##
## data:  data_sub_con$infant_age_ICEP and data_sub_anx$infant_age_ICEP
## t = -1.0412, df = 56, p-value = 0.3023
## alternative hypothesis: true difference in means is not equal to 0
## 95 percent confidence interval:
##  -0.8713014  0.2753418
## sample estimates:
## mean of x mean of y
##  3.586111  3.884091
```

```
t.test(data_sub_con$APGAR_1,data_sub_anx$APGAR_1, alternative = "two.sided", var.eq
ual=TRUE, conf.level = 0.95)
```

```
##
##  Two Sample t-test
##
## data:  data_sub_con$APGAR_1 and data_sub_anx$APGAR_1
## t = 1.7856, df = 47, p-value = 0.08061
## alternative hypothesis: true difference in means is not equal to 0
## 95 percent confidence interval:
##  -0.06052067  1.01640303
## sample estimates:
## mean of x mean of y
##  9.125000  8.647059
```

```
t.test(data_sub_con$APGAR_5,data_sub_anx$APGAR_5, alternative = "two.sided", var.eq
ual=TRUE, conf.level = 0.95)
```

File failed to load: file:///C:/Users/mitho/Dokumente/B%C3%BCro/DFG-Angst/Paper%20Nathania/Repair_Attachment/manuscript/Review%

```
##
##  Two Sample t-test
##
## data:  data_sub_con$APGAR_5 and data_sub_anx$APGAR_5
## t = 1.5947, df = 46, p-value = 0.1176
## alternative hypothesis: true difference in means is not equal to 0
## 95 percent confidence interval:
##  -0.09034068  0.77922957
## sample estimates:
## mean of x mean of y
##  9.733333  9.388889
```

```
t.test(data_sub_con$APGAR_10,data_sub_anx$APGAR_10, alternative = "two.sided", var.
equal=TRUE, conf.level = 0.95)
```

```
##
##  Two Sample t-test
##
## data:  data_sub_con$APGAR_10 and data_sub_anx$APGAR_10
## t = 0.82312, df = 47, p-value = 0.4146
## alternative hypothesis: true difference in means is not equal to 0
## 95 percent confidence interval:
##  -0.1596055  0.3806581
## sample estimates:
## mean of x mean of y
##  9.900000  9.789474
```

```
t.test(data_sub_con$infant_age_A4,data_sub_anx$infant_age_A4, alternative = "two.si
ded", var.equal=TRUE, conf.level = 0.95)
```

```
##
##  Two Sample t-test
##
## data:  data_sub_con$infant_age_A4 and data_sub_anx$infant_age_A4
## t = -1.4937, df = 41, p-value = 0.1429
## alternative hypothesis: true difference in means is not equal to 0
## 95 percent confidence interval:
##  -1.4927660  0.2234326
## sample estimates:
## mean of x mean of y
##  18.93200  19.56667
```

Ordinal variables

```
# general medians
data_sub %>%
   summarise(maternal_education_med = median(as.numeric(maternal_education)),
             amount_infants_med = median(amount_infants),
             count_anxiety_diagnoses_med = median(count_anxiety_diagnoses))
```

File failed to load: file:///C:/Users/mitho/Dokumente/B%C3%BCro/DFG-Angst/Paper%20Nathania/Repair_Attachment/manuscript/Review%

```
## # A tibble: 1 x 3
##   maternal_education_med amount_infants_med count_anxiety_diagnoses_med
##                    <dbl>              <dbl>                       <dbl>
## 1                      4                  1                           0
```

```
# general frequencies
table(data_sub$maternal_education)
```

```
## 
##       Low secondary qualification      High secondary qualification 
##                                 1                                13 
## University entrance qualification                 University degree 
##                                11                                33
```

```
table(data_sub$amount_infants)
```

```
## 
##  1  2  3  4 
## 33 19  5  1
```

```
table(data_sub$count_anxiety_diagnoses)
```

```
## 
##  0  1  2  3  4 
## 36  8  8  4  2
```

```
# group-specific medians
data_sub %>%
   group_by(diagnosis) %>%
   summarise(maternal_education_med = median(as.numeric(maternal_education)),
             amount_infants_med = median(amount_infants),
             count_anxiety_diagnoses_med = median(count_anxiety_diagnoses))
```

```
## # A tibble: 2 x 4
##   diagnosis maternal_education_med amount_infants_med count_anxiety_diagnoses_m~
##   <fct>                      <dbl>              <dbl>                      <dbl>
## 1 Control                        4                  2                          0
## 2 Clinical                       4                  1                          2
```

```
# group-specific frequencies
table(data_sub$maternal_education, data_sub$diagnosis)
```

File failed to load: file:///C:/Users/mitho/Dokumente/B%C3%BCro/DFG-Angst/Paper%20Nathania/Repair_Attachment/manuscript/Review%

```
##
##                                      Control Clinical
##   Low secondary qualification              1        0
##   High secondary qualification             7        6
##   University entrance qualification        7        4
##   University degree                       21       12
```

```
table(data_sub$amount_infants, data_sub$diagnosis)
```

```
##
##     Control Clinical
##   1      16       17
##   2      15        4
##   3       4        1
##   4       1        0
```

```
table(data_sub$count_anxiety_diagnoses, data_sub$diagnosis)
```

```
##
##     Control Clinical
##   0      36        0
##   1       0        8
##   2       0        8
##   3       0        4
##   4       0        2
```

```
# tests on comparability
wilcox.test(as.numeric(maternal_education) ~ diagnosis, data = data_sub)
```

```
## Warning in wilcox.test.default(x = c(3, 4, 4, 4, 4, 4, 3, 4, 4, 4, 4, 4, :
## cannot compute exact p-value with ties
```

```
##
##  Wilcoxon rank sum test with continuity correction
##
## data:  as.numeric(maternal_education) by diagnosis
## W = 413, p-value = 0.7672
## alternative hypothesis: true location shift is not equal to 0
```

```
wilcox.test(amount_infants ~ diagnosis, data = data_sub)
```

```
## Warning in wilcox.test.default(x = c(1, 1, 2, 1, 2, 1, 3, 2, 1, 2, 1, 1, :
## cannot compute exact p-value with ties
```

File failed to load: file:///C:/Users/mitho/Dokumente/B%C3%BCro/DFG-Angst/Paper%20Nathania/Repair_Attachment/manuscript/Review%

```
##
##  Wilcoxon rank sum test with continuity correction
##
## data:  amount_infants by diagnosis
## W = 529, p-value = 0.01622
## alternative hypothesis: true location shift is not equal to 0
```

Nominal variables

```
# Diagnoses
table(data_sub$diagnosis)
```

```
##
##  Control Clinical
##       36       22
```

```
table(data_sub$panic_disorder_agoraphobia, data_sub$diagnosis)
```

```
##
##         Control Clinical
##   False       0       12
##   True        0       10
```

```
table(data_sub$sociophobia, data_sub$diagnosis)
```

```
##
##         Control Clinical
##   False       0       16
##   True        0        6
```

```
table(data_sub$specific_phobia, data_sub$diagnosis)
```

```
##
##         Control Clinical
##   False       0       16
##   True        0        6
```

```
table(data_sub$obsessive_compulsive_disorder, data_sub$diagnosis)
```

```
##
##         Control Clinical
##   False       0       14
##   True        0        8
```

```
table(data_sub$posttraumatic_stress_disorder, data_sub$diagnosis)
```

File failed to load: file:///C:/Users/mitho/Dokumente/B%C3%BCro/DFG-Angst/Paper%20Nathania/Repair_Attachment/manuscript/Review%

```
##
##         Control Clinical
##   False       0       21
##   True        0        1
```

```
table(data_sub$generalized_anxiety_disorder, data_sub$diagnosis)
```

```
##
##         Control Clinical
##   False       0       10
##   True        0       12
```

```
table(data_sub$anxiety_disorder_nos, data_sub$diagnosis)
```

```
##
##         Control Clinical
##   False       0       21
##   True        0        1
```

```
table(data_sub$panic_disorder_agoraphobia_onset, data_sub$diagnosis)
```

```
##
##        Control Clinical
##   Pre        0        9
##   Peri       0        1
##   Post       0        0
```

```
table(data_sub$sociophobia_onset, data_sub$diagnosis)
```

```
##
##       Control Clinical
##   Pre       0        6
```

```
table(data_sub$specific_phobia_onset, data_sub$diagnosis)
```

```
##
##       Control Clinical
##   Pre       0        6
```

```
table(data_sub$obsessive_compulsive_disorder_onset, data_sub$diagnosis)
```

```
##
##        Control Clinical
##   Pre        0        5
##   Peri       0        0
##   Post       0        2
```

File failed to load: file:///C:/Users/mitho/Dokumente/B%C3%BCro/DFG-Angst/Paper%20Nathania/Repair_Attachment/manuscript/Review%

```
table(data_sub$posttraumatic_stress_disorder_onset, data_sub$diagnosis)
```

```
##
##     Control Clinical
##   0       0        1
```

```
table(data_sub$generalized_anxiety_disorder_onset, data_sub$diagnosis)
```

```
##
##        Control Clinical
##   Pre        0        6
##   Peri       0        3
##   Post       0        3
```

```
table(data_sub$anxiety_disorder_nos_onset, data_sub$diagnosis)
```

```
##
##        Control Clinical
##   Peri       0        1
##   Post       0        0
```

```
table(data_sub$anxiety_disorders_early_onset, data_sub$diagnosis)
```

```
##
##        Control Clinical
##   Pre        0       16
##   Peri       0        3
##   Post       0        3
```

```
table(data_sub$inpatients, data_sub$diagnosis)
```

```
##
##         Control Clinical
##   False      36       17
##   True        0        5
```

```
# General and specific sociodemographics & tests on comparability
table(data_sub$social_marriage)
```

```
##
## False  True
##    15    40
```

```
table(data_sub$social_marriage, data_sub$diagnosis)
```

File failed to load: file:///C:/Users/mitho/Dokumente/B%C3%BCro/DFG-Angst/Paper%20Nathania/Repair_Attachment/manuscript/Review%

```
##
##         Control Clinical
##   False       8        7
##   True       26       14
```

```
fisher.test(data_sub$social_marriage, data_sub$diagnosis)
```

```
##
##  Fisher's Exact Test for Count Data
##
## data:  data_sub$social_marriage and data_sub$diagnosis
## p-value = 0.5366
## alternative hypothesis: true odds ratio is not equal to 1
## 95 percent confidence interval:
##  0.1572259 2.4736325
## sample estimates:
## odds ratio
##  0.6210338
```

```
table(data_sub$birth_PDA_epidural)
```

```
##
## False  True
##    37    20
```

```
table(data_sub$birth_PDA_epidural, data_sub$diagnosis)
```

```
##
##         Control Clinical
##   False      23       14
##   True       12        8
```

```
fisher.test(data_sub$birth_PDA_epidural, data_sub$diagnosis)
```

```
##
##  Fisher's Exact Test for Count Data
##
## data:  data_sub$birth_PDA_epidural and data_sub$diagnosis
## p-value = 1
## alternative hypothesis: true odds ratio is not equal to 1
## 95 percent confidence interval:
##  0.3056895 3.8044614
## sample estimates:
## odds ratio
##   1.093484
```

```
table(data_sub$birth_mode)
```

File failed to load: file:///C:/Users/mitho/Dokumente/B%C3%BCro/DFG-Angst/Paper%20Nathania/Repair_Attachment/manuscript/Review%

```
##
##      Spontaneous   Primary sectio Secondary sectio
##               39               12                6
```

```
table(data_sub$birth_mode, data_sub$diagnosis)
```

```
##
##                     Control Clinical
##   Spontaneous            25       14
##   Primary sectio          6        6
##   Secondary sectio        4        2
```

```
fisher.test(data_sub$birth_mode, data_sub$diagnosis, simulate.p.value = TRUE, B = 5
000) # frequencies below 5
```

```
##
##  Fisher's Exact Test for Count Data with simulated p-value (based on
##  5000 replicates)
##
## data:  data_sub$birth_mode and data_sub$diagnosis
## p-value = 0.6881
## alternative hypothesis: two.sided
```

```
table(data_sub$infant_sex_index_infant)
```

```
##
## Female   Male
##     34     24
```

```
table(data_sub$infant_sex_index_infant, data_sub$diagnosis)
```

```
##
##          Control Clinical
##   Female      19       15
##   Male        17        7
```

```
fisher.test(data_sub$infant_sex_index_infant, data_sub$diagnosis)
```

File failed to load: file:///C:/Users/mitho/Dokumente/B%C3%BCro/DFG-Angst/Paper%20Nathania/Repair_Attachment/manuscript/Review%

```
##
##  Fisher's Exact Test for Count Data
##
## data:  data_sub$infant_sex_index_infant and data_sub$diagnosis
## p-value = 0.2837
## alternative hypothesis: true odds ratio is not equal to 1
## 95 percent confidence interval:
##  0.1447294 1.7874985
## sample estimates:
## odds ratio
##  0.5274379
```

Descriptives study variables

```
# Parametric
## general means
desc_par_out_g <- data_sub %>%
   summarise(across(c(CPOS_e_CPVC_e_IPOS_INEU_latency_P3,
                      PESI_3rd_trimenon_cor),
                    list(mean = vm, sd = vsd, se = vse, min = vmin, max = vmax, n =
vnc)))
View(desc_par_out_g)
write_xlsx(desc_par_out_g, "desc_par_out_g.xlsx")

## group-specific means
desc_par_out_s <- data_sub %>%
   group_by(diagnosis) %>%
   summarise(across(c(CPOS_e_CPVC_e_IPOS_INEU_latency_P3,
                      PESI_3rd_trimenon_cor),
                    list(mean = vm, sd = vsd, se = vse, min = vmin, max = vmax, n =
vnc)))
View(desc_par_out_s)
write_xlsx(desc_par_out_s, "desc_par_out_s.xlsx")

# Nominal
## general and specific frequencies
table(data_sub$match_event)
```

```
##
## False  True
##     9    49
```

```
table(data_sub$match_event, data_sub$diagnosis)
```

```
##
##          Control Clinical
##   False        5        4
##   True        31       18
```

```
table(data_sub$FST_ges)
```

File failed to load: file:///C:/Users/mitho/Dokumente/B%C3%BCro/DFG-Angst/Paper%20Nathania/Repair_Attachment/manuscript/Review%

```
##
##  A  B  C  D
## 13 37  1  7
```

```
table(data_sub$FST_ges, data_sub$diagnosis)
```

```
##
##     Control Clinical
##   A       6        7
##   B      28        9
##   C       1        0
##   D       1        6
```

```
table(data_sub$FST_numb)
```

```
##
##   Secure Insecure
##       37       21
```

```
table(data_sub$FST_numb, data_sub$diagnosis)
```

```
##
##            Control Clinical
##   Secure        28        9
##   Insecure       8       13
```

Confounder Analysis

```
# Spearman-Correlations between study variables and ordinal number of infants as si
gnificantly different between diagnostic groups
cor.test(data_sub$CPOS_e_CPVC_e_IPOS_INEU_latency_P3, data_sub$amount_infants, meth
od = "spearman")
```

```
## Warning in cor.test.default(data_sub$CPOS_e_CPVC_e_IPOS_INEU_latency_P3, :
## Cannot compute exact p-value with ties
```

```
##
##  Spearman's rank correlation rho
##
## data:  data_sub$CPOS_e_CPVC_e_IPOS_INEU_latency_P3 and data_sub$amount_infants
## S = 21815, p-value = 0.4394
## alternative hypothesis: true rho is not equal to 0
## sample estimates:
##        rho
## -0.1130139
```

File failed to load: file:///C:/Users/mitho/Dokumente/B%C3%BCro/DFG-Angst/Paper%20Nathania/Repair_Attachment/manuscript/Review%

```
cor.test(data_sub$PESI_3rd_trimenon_cor, data_sub$amount_infants, method = "spearma
n")
```

```
## Warning in cor.test.default(data_sub$PESI_3rd_trimenon_cor,
## data_sub$amount_infants, : Cannot compute exact p-value with ties
```

```
##
##  Spearman's rank correlation rho
##
## data:  data_sub$PESI_3rd_trimenon_cor and data_sub$amount_infants
## S = 30395, p-value = 0.4833
## alternative hypothesis: true rho is not equal to 0
## sample estimates:
##         rho
## -0.09650897
```

```
cor.test(as.numeric(data_sub$match_event), data_sub$amount_infants, method = "spear
man")
```

```
## Warning in cor.test.default(as.numeric(data_sub$match_event),
## data_sub$amount_infants, : Cannot compute exact p-value with ties
```

```
##
##  Spearman's rank correlation rho
##
## data:  as.numeric(data_sub$match_event) and data_sub$amount_infants
## S = 35597, p-value = 0.4781
## alternative hypothesis: true rho is not equal to 0
## sample estimates:
##         rho
## -0.09499796
```

```
cor.test(as.numeric(data_sub$FST_numb), data_sub$amount_infants, method = "spearma
n")
```

```
## Warning in cor.test.default(as.numeric(data_sub$FST_numb),
## data_sub$amount_infants, : Cannot compute exact p-value with ties
```

```
##
##  Spearman's rank correlation rho
##
## data:  as.numeric(data_sub$FST_numb) and data_sub$amount_infants
## S = 37912, p-value = 0.2125
## alternative hypothesis: true rho is not equal to 0
## sample estimates:
##        rho
## -0.1661849
```

File failed to load: file:///C:/Users/mitho/Dokumente/B%C3%BCro/DFG-Angst/Paper%20Nathania/Repair_Attachment/manuscript/Review%

Main analysis

Hierarchical generalized binomial regression onto binary attachment (backwards)

```
model_1 <- glm(FST_numb~diagnosis+
                  CPOS_e_CPVC_e_IPOS_INEU_latency_P3+
                  PESI_3rd_trimenon_cor+
                  diagnosis:CPOS_e_CPVC_e_IPOS_INEU_latency_P3+
                  diagnosis:PESI_3rd_trimenon_cor+
                  CPOS_e_CPVC_e_IPOS_INEU_latency_P3:PESI_3rd_trimenon_cor+
                  diagnosis:CPOS_e_CPVC_e_IPOS_INEU_latency_P3:PESI_3rd_trimenon_co
r,
               family=binomial(link=logit),
               data=data_sub)
summary (model_1) # model summary
```

File failed to load: file:///C:/Users/mitho/Dokumente/B%C3%BCro/DFG-Angst/Paper%20Nathania/Repair_Attachment/manuscript/Review%

```
##
## Call:
## glm(formula = FST_numb ~ diagnosis + CPOS_e_CPVC_e_IPOS_INEU_latency_P3 +
##     PESI_3rd_trimenon_cor + diagnosis:CPOS_e_CPVC_e_IPOS_INEU_latency_P3 +
##     diagnosis:PESI_3rd_trimenon_cor + CPOS_e_CPVC_e_IPOS_INEU_latency_P3:PESI_3r
d_trimenon_cor +
##     diagnosis:CPOS_e_CPVC_e_IPOS_INEU_latency_P3:PESI_3rd_trimenon_cor,
##     family = binomial(link = logit), data = data_sub)
##
## Deviance Residuals:
##     Min       1Q   Median       3Q      Max
## -1.5908  -0.5517  -0.4142   0.3532   2.3588
##
## Coefficients:
##                                                                       Estim
ate
## (Intercept)                                                           -2.58
472
## diagnosisClinical                                                     -0.97
358
## CPOS_e_CPVC_e_IPOS_INEU_latency_P3                                    -0.20
221
## PESI_3rd_trimenon_cor                                                  0.03
330
## diagnosisClinical:CPOS_e_CPVC_e_IPOS_INEU_latency_P3                   0.49
282
## diagnosisClinical:PESI_3rd_trimenon_cor                                0.01
857
## CPOS_e_CPVC_e_IPOS_INEU_latency_P3:PESI_3rd_trimenon_cor               0.01
493
## diagnosisClinical:CPOS_e_CPVC_e_IPOS_INEU_latency_P3:PESI_3rd_trimenon_cor -0.01
790
##                                                                       Std.
Error
## (Intercept)                                                              1.
41742
## diagnosisClinical                                                        3.
06776
## CPOS_e_CPVC_e_IPOS_INEU_latency_P3                                       0.
23733
## PESI_3rd_trimenon_cor                                                    0.
06943
## diagnosisClinical:CPOS_e_CPVC_e_IPOS_INEU_latency_P3                     0.
41214
## diagnosisClinical:PESI_3rd_trimenon_cor                                  0.
08064
## CPOS_e_CPVC_e_IPOS_INEU_latency_P3:PESI_3rd_trimenon_cor                 0.
01439
## diagnosisClinical:CPOS_e_CPVC_e_IPOS_INEU_latency_P3:PESI_3rd_trimenon_cor    0.
01509
##                                                                       z val
ue
## (Intercept)                                                            -1.8
24
```

File failed to load: file:///C:/Users/mitho/Dokumente/B%C3%BCro/DFG-Angst/Paper%20Nathania/Repair_Attachment/manuscript/Review%

```
## diagnosisClinical                                                       -0.3
17
## CPOS_e_CPVC_e_IPOS_INEU_latency_P3                                      -0.8
52
## PESI_3rd_trimenon_cor                                                    0.4
80
## diagnosisClinical:CPOS_e_CPVC_e_IPOS_INEU_latency_P3                     1.1
96
## diagnosisClinical:PESI_3rd_trimenon_cor                                  0.2
30
## CPOS_e_CPVC_e_IPOS_INEU_latency_P3:PESI_3rd_trimenon_cor                 1.0
38
## diagnosisClinical:CPOS_e_CPVC_e_IPOS_INEU_latency_P3:PESI_3rd_trimenon_cor  -1.1
86
##                                                                        Pr(>|
z|)
## (Intercept)                                                              0.0
682
## diagnosisClinical                                                        0.7
510
## CPOS_e_CPVC_e_IPOS_INEU_latency_P3                                       0.3
942
## PESI_3rd_trimenon_cor                                                    0.6
316
## diagnosisClinical:CPOS_e_CPVC_e_IPOS_INEU_latency_P3                     0.2
318
## diagnosisClinical:PESI_3rd_trimenon_cor                                  0.8
179
## CPOS_e_CPVC_e_IPOS_INEU_latency_P3:PESI_3rd_trimenon_cor                 0.2
994
## diagnosisClinical:CPOS_e_CPVC_e_IPOS_INEU_latency_P3:PESI_3rd_trimenon_cor   0.2
358
##
## (Intercept)                                                            .
## diagnosisClinical
## CPOS_e_CPVC_e_IPOS_INEU_latency_P3
## PESI_3rd_trimenon_cor
## diagnosisClinical:CPOS_e_CPVC_e_IPOS_INEU_latency_P3
## diagnosisClinical:PESI_3rd_trimenon_cor
## CPOS_e_CPVC_e_IPOS_INEU_latency_P3:PESI_3rd_trimenon_cor
## diagnosisClinical:CPOS_e_CPVC_e_IPOS_INEU_latency_P3:PESI_3rd_trimenon_cor
## ---
## Signif. codes:  0 '***' 0.001 '**' 0.01 '*' 0.05 '.' 0.1 ' ' 1
##
## (Dispersion parameter for binomial family taken to be 1)
##
##     Null deviance: 56.534  on 45  degrees of freedom
## Residual deviance: 37.962  on 38  degrees of freedom
##   (12 Beobachtungen als fehlend gelöscht)
## AIC: 53.962
##
## Number of Fisher Scoring iterations: 7
```

```
confint(model_1) # 95% CI for the coefficients
```

File failed to load: file:///C:/Users/mitho/Dokumente/B%C3%BCro/DFG-Angst/Paper%20Nathania/Repair_Attachment/manuscript/Review%

```
## Waiting for profiling to be done...
```

```
## Warning: glm.fit: fitted probabilities numerically 0 or 1 occurred

## Warning: glm.fit: fitted probabilities numerically 0 or 1 occurred

## Warning: glm.fit: fitted probabilities numerically 0 or 1 occurred

## Warning: glm.fit: fitted probabilities numerically 0 or 1 occurred

## Warning: glm.fit: fitted probabilities numerically 0 or 1 occurred

## Warning: glm.fit: fitted probabilities numerically 0 or 1 occurred

## Warning: glm.fit: fitted probabilities numerically 0 or 1 occurred

## Warning: glm.fit: fitted probabilities numerically 0 or 1 occurred

## Warning: glm.fit: fitted probabilities numerically 0 or 1 occurred
```

File failed to load: file:///C:/Users/mitho/Dokumente/B%C3%BCro/DFG-Angst/Paper%20Nathania/Repair_Attachment/manuscript/Review%

```
##
2.5 %
## (Intercept)                                                                -5.82
3101692
## diagnosisClinical                                                          -8.87
4882972
## CPOS_e_CPVC_e_IPOS_INEU_latency_P3                                         -0.85
5873196
## PESI_3rd_trimenon_cor                                                      -0.11
9022736
## diagnosisClinical:CPOS_e_CPVC_e_IPOS_INEU_latency_P3                       -0.11
0413274
## diagnosisClinical:PESI_3rd_trimenon_cor                                    -0.13
5448683
## CPOS_e_CPVC_e_IPOS_INEU_latency_P3:PESI_3rd_trimenon_cor                   -0.00
6591964
## diagnosisClinical:CPOS_e_CPVC_e_IPOS_INEU_latency_P3:PESI_3rd_trimenon_cor -0.05
8486379
##
97.5 %
## (Intercept)                                                                0.048
394562
## diagnosisClinical                                                          4.180
797687
## CPOS_e_CPVC_e_IPOS_INEU_latency_P3                                         0.139
879763
## PESI_3rd_trimenon_cor                                                      0.170
969146
## diagnosisClinical:CPOS_e_CPVC_e_IPOS_INEU_latency_P3                       1.615
614330
## diagnosisClinical:PESI_3rd_trimenon_cor                                    0.195
642284
## CPOS_e_CPVC_e_IPOS_INEU_latency_P3:PESI_3rd_trimenon_cor                   0.054
716869
## diagnosisClinical:CPOS_e_CPVC_e_IPOS_INEU_latency_P3:PESI_3rd_trimenon_cor 0.006
819804
```

```
exp(coef(model_1)) # exponentiated coefficients
```

File failed to load: file:///C:/Users/mitho/Dokumente/B%C3%BCro/DFG-Angst/Paper%20Nathania/Repair_Attachment/manuscript/Review%

```
##                                                              (Intercept)
##                                                               0.07541749
##                                                        diagnosisClinical
##                                                               0.37772681
##                                     CPOS_e_CPVC_e_IPOS_INEU_latency_P3
##                                                               0.81692381
##                                                 PESI_3rd_trimenon_cor
##                                                               1.03385716
##                   diagnosisClinical:CPOS_e_CPVC_e_IPOS_INEU_latency_P3
##                                                               1.63692188
##                               diagnosisClinical:PESI_3rd_trimenon_cor
##                                                               1.01874407
##                CPOS_e_CPVC_e_IPOS_INEU_latency_P3:PESI_3rd_trimenon_cor
##                                                               1.01504422
## diagnosisClinical:CPOS_e_CPVC_e_IPOS_INEU_latency_P3:PESI_3rd_trimenon_cor
##                                                               0.98226391
```

```
exp(confint(model_1)) # 95% CI for exponentiated coefficients
```

```
## Waiting for profiling to be done...
```

```
## Warning: glm.fit: fitted probabilities numerically 0 or 1 occurred

## Warning: glm.fit: fitted probabilities numerically 0 or 1 occurred

## Warning: glm.fit: fitted probabilities numerically 0 or 1 occurred

## Warning: glm.fit: fitted probabilities numerically 0 or 1 occurred

## Warning: glm.fit: fitted probabilities numerically 0 or 1 occurred

## Warning: glm.fit: fitted probabilities numerically 0 or 1 occurred

## Warning: glm.fit: fitted probabilities numerically 0 or 1 occurred

## Warning: glm.fit: fitted probabilities numerically 0 or 1 occurred

## Warning: glm.fit: fitted probabilities numerically 0 or 1 occurred
```

File failed to load: file:///C:/Users/mitho/Dokumente/B%C3%BCro/DFG-Angst/Paper%20Nathania/Repair_Attachment/manuscript/Review%

```
##
2.5 %
## (Intercept)                                                               0.002
958415
## diagnosisClinical                                                         0.000
139858
## CPOS_e_CPVC_e_IPOS_INEU_latency_P3                                        0.424
911997
## PESI_3rd_trimenon_cor                                                     0.887
787616
## diagnosisClinical:CPOS_e_CPVC_e_IPOS_INEU_latency_P3                      0.895
463987
## diagnosisClinical:PESI_3rd_trimenon_cor                                   0.873
323978
## CPOS_e_CPVC_e_IPOS_INEU_latency_P3:PESI_3rd_trimenon_cor                  0.993
429716
## diagnosisClinical:CPOS_e_CPVC_e_IPOS_INEU_latency_P3:PESI_3rd_trimenon_cor 0.943
191087
##                                                                             9
7.5 %
## (Intercept)                                                                1.04
9585
## diagnosisClinical                                                         65.41
8016
## CPOS_e_CPVC_e_IPOS_INEU_latency_P3                                         1.15
0136
## PESI_3rd_trimenon_cor                                                      1.18
6454
## diagnosisClinical:CPOS_e_CPVC_e_IPOS_INEU_latency_P3                       5.03
0978
## diagnosisClinical:PESI_3rd_trimenon_cor                                    1.21
6092
## CPOS_e_CPVC_e_IPOS_INEU_latency_P3:PESI_3rd_trimenon_cor                   1.05
6242
## diagnosisClinical:CPOS_e_CPVC_e_IPOS_INEU_latency_P3:PESI_3rd_trimenon_cor  1.00
6843
```

```
anova(model_1, test = "Chisq") # Wald coefficient test
```

File failed to load: file:///C:/Users/mitho/Dokumente/B%C3%BCro/DFG-Angst/Paper%20Nathania/Repair_Attachment/manuscript/Review%

```
## Analysis of Deviance Table
##
## Model: binomial, link: logit
##
## Response: FST_numb
##
## Terms added sequentially (first to last)
##
##
##                                                                     Df Deviance
## NULL
## diagnosis                                                            1   5.3443
## CPOS_e_CPVC_e_IPOS_INEU_latency_P3                                   1   4.7806
## PESI_3rd_trimenon_cor                                                1   4.2754
## diagnosis:CPOS_e_CPVC_e_IPOS_INEU_latency_P3                         1   1.6103
## diagnosis:PESI_3rd_trimenon_cor                                      1   0.5917
## CPOS_e_CPVC_e_IPOS_INEU_latency_P3:PESI_3rd_trimenon_cor             1   0.0342
## diagnosis:CPOS_e_CPVC_e_IPOS_INEU_latency_P3:PESI_3rd_trimenon_cor  1   1.9362
##                                                                     Resid. Df
## NULL                                                                       45
## diagnosis                                                                  44
## CPOS_e_CPVC_e_IPOS_INEU_latency_P3                                         43
## PESI_3rd_trimenon_cor                                                      42
## diagnosis:CPOS_e_CPVC_e_IPOS_INEU_latency_P3                               41
## diagnosis:PESI_3rd_trimenon_cor                                            40
## CPOS_e_CPVC_e_IPOS_INEU_latency_P3:PESI_3rd_trimenon_cor                   39
## diagnosis:CPOS_e_CPVC_e_IPOS_INEU_latency_P3:PESI_3rd_trimenon_cor        38
##                                                                     Resid. Dev
## NULL                                                                    56.534
## diagnosis                                                               51.190
## CPOS_e_CPVC_e_IPOS_INEU_latency_P3                                      46.409
## PESI_3rd_trimenon_cor                                                   42.134
## diagnosis:CPOS_e_CPVC_e_IPOS_INEU_latency_P3                            40.524
## diagnosis:PESI_3rd_trimenon_cor                                         39.932
## CPOS_e_CPVC_e_IPOS_INEU_latency_P3:PESI_3rd_trimenon_cor                39.898
## diagnosis:CPOS_e_CPVC_e_IPOS_INEU_latency_P3:PESI_3rd_trimenon_cor     37.962
##                                                                     Pr(>Chi)
## NULL
## diagnosis                                                            0.02079 *
## CPOS_e_CPVC_e_IPOS_INEU_latency_P3                                   0.02878 *
## PESI_3rd_trimenon_cor                                                0.03867 *
## diagnosis:CPOS_e_CPVC_e_IPOS_INEU_latency_P3                         0.20445
## diagnosis:PESI_3rd_trimenon_cor                                      0.44176
## CPOS_e_CPVC_e_IPOS_INEU_latency_P3:PESI_3rd_trimenon_cor             0.85329
## diagnosis:CPOS_e_CPVC_e_IPOS_INEU_latency_P3:PESI_3rd_trimenon_cor  0.16408
## ---
## Signif. codes:  0 '***' 0.001 '**' 0.01 '*' 0.05 '.' 0.1 ' ' 1
```

File failed to load: file:///C:/Users/mitho/Dokumente/B%C3%BCro/DFG-Angst/Paper%20Nathania/Repair_Attachment/manuscript/Review%

```
model_2 <- glm(FST_numb~diagnosis+
                  CPOS_e_CPVC_e_IPOS_INEU_latency_P3+
                  PESI_3rd_trimenon_cor+
                  diagnosis:CPOS_e_CPVC_e_IPOS_INEU_latency_P3+
                  diagnosis:PESI_3rd_trimenon_cor+
                  diagnosis:CPOS_e_CPVC_e_IPOS_INEU_latency_P3:PESI_3rd_trimenon_co
r,
               family=binomial(link=logit),
               data=data_sub)
summary(model_2) # model summary
```

File failed to load: file:///C:/Users/mitho/Dokumente/B%C3%BCro/DFG-Angst/Paper%20Nathania/Repair_Attachment/manuscript/Review%

```
##
## Call:
## glm(formula = FST_numb ~ diagnosis + CPOS_e_CPVC_e_IPOS_INEU_latency_P3 +
##     PESI_3rd_trimenon_cor + diagnosis:CPOS_e_CPVC_e_IPOS_INEU_latency_P3 +
##     diagnosis:PESI_3rd_trimenon_cor + diagnosis:CPOS_e_CPVC_e_IPOS_INEU_latency_
P3:PESI_3rd_trimenon_cor,
##     family = binomial(link = logit), data = data_sub)
##
## Deviance Residuals:
##     Min       1Q   Median       3Q      Max
## -1.5908  -0.5517  -0.4142   0.3532   2.3588
##
## Coefficients:
##                                                                         Esti
mate
## (Intercept)                                                            -2.58
4716
## diagnosisClinical                                                      -0.97
3584
## CPOS_e_CPVC_e_IPOS_INEU_latency_P3                                     -0.20
2209
## PESI_3rd_trimenon_cor                                                   0.03
3297
## diagnosisClinical:CPOS_e_CPVC_e_IPOS_INEU_latency_P3                    0.49
2818
## diagnosisClinical:PESI_3rd_trimenon_cor                                 0.01
8571
## diagnosisControl:CPOS_e_CPVC_e_IPOS_INEU_latency_P3:PESI_3rd_trimenon_cor   0.01
4932
## diagnosisClinical:CPOS_e_CPVC_e_IPOS_INEU_latency_P3:PESI_3rd_trimenon_cor -0.00
2963
##                                                                        Std. 
Error
## (Intercept)                                                              1.4
17422
## diagnosisClinical                                                        3.0
67757
## CPOS_e_CPVC_e_IPOS_INEU_latency_P3                                       0.2
37330
## PESI_3rd_trimenon_cor                                                    0.0
69434
## diagnosisClinical:CPOS_e_CPVC_e_IPOS_INEU_latency_P3                     0.4
12142
## diagnosisClinical:PESI_3rd_trimenon_cor                                  0.0
80640
## diagnosisControl:CPOS_e_CPVC_e_IPOS_INEU_latency_P3:PESI_3rd_trimenon_cor    0.0
14390
## diagnosisClinical:CPOS_e_CPVC_e_IPOS_INEU_latency_P3:PESI_3rd_trimenon_cor   0.0
04556
##                                                                        z val
ue
## (Intercept)                                                             -1.8
24
## diagnosisClinical                                                       -0.3
```

File failed to load: file:///C:/Users/mitho/Dokumente/B%C3%BCro/DFG-Angst/Paper%20Nathania/Repair_Attachment/manuscript/Review%

```
17
## CPOS_e_CPVC_e_IPOS_INEU_latency_P3                                        -0.8
52
## PESI_3rd_trimenon_cor                                                       0.4
80
## diagnosisClinical:CPOS_e_CPVC_e_IPOS_INEU_latency_P3                       1.1
96
## diagnosisClinical:PESI_3rd_trimenon_cor                                     0.2
30
## diagnosisControl:CPOS_e_CPVC_e_IPOS_INEU_latency_P3:PESI_3rd_trimenon_cor    1.0
38
## diagnosisClinical:CPOS_e_CPVC_e_IPOS_INEU_latency_P3:PESI_3rd_trimenon_cor  -0.6
50
##                                                                          Pr(>|
z|)
## (Intercept)                                                                 0.0
682
## diagnosisClinical                                                           0.7
510
## CPOS_e_CPVC_e_IPOS_INEU_latency_P3                                          0.3
942
## PESI_3rd_trimenon_cor                                                       0.6
316
## diagnosisClinical:CPOS_e_CPVC_e_IPOS_INEU_latency_P3                       0.2
318
## diagnosisClinical:PESI_3rd_trimenon_cor                                     0.8
179
## diagnosisControl:CPOS_e_CPVC_e_IPOS_INEU_latency_P3:PESI_3rd_trimenon_cor    0.2
994
## diagnosisClinical:CPOS_e_CPVC_e_IPOS_INEU_latency_P3:PESI_3rd_trimenon_cor   0.5
154
##
## (Intercept)                                                               .
## diagnosisClinical
## CPOS_e_CPVC_e_IPOS_INEU_latency_P3
## PESI_3rd_trimenon_cor
## diagnosisClinical:CPOS_e_CPVC_e_IPOS_INEU_latency_P3
## diagnosisClinical:PESI_3rd_trimenon_cor
## diagnosisControl:CPOS_e_CPVC_e_IPOS_INEU_latency_P3:PESI_3rd_trimenon_cor
## diagnosisClinical:CPOS_e_CPVC_e_IPOS_INEU_latency_P3:PESI_3rd_trimenon_cor
## ---
## Signif. codes:  0 '***' 0.001 '**' 0.01 '*' 0.05 '.' 0.1 ' ' 1
##
## (Dispersion parameter for binomial family taken to be 1)
##
##     Null deviance: 56.534  on 45  degrees of freedom
## Residual deviance: 37.962  on 38  degrees of freedom
##   (12 Beobachtungen als fehlend gelöscht)
## AIC: 53.962
##
## Number of Fisher Scoring iterations: 7
```

```
confint(model_2) # 95% CI for the coefficients
```

File failed to load: file:///C:/Users/mitho/Dokumente/B%C3%BCro/DFG-Angst/Paper%20Nathania/Repair_Attachment/manuscript/Review%

```
## Waiting for profiling to be done...
```

```
## Warning: glm.fit: fitted probabilities numerically 0 or 1 occurred

## Warning: glm.fit: fitted probabilities numerically 0 or 1 occurred

## Warning: glm.fit: fitted probabilities numerically 0 or 1 occurred

## Warning: glm.fit: fitted probabilities numerically 0 or 1 occurred

## Warning: glm.fit: fitted probabilities numerically 0 or 1 occurred

## Warning: glm.fit: fitted probabilities numerically 0 or 1 occurred

## Warning: glm.fit: fitted probabilities numerically 0 or 1 occurred

## Warning: glm.fit: fitted probabilities numerically 0 or 1 occurred

## Warning: glm.fit: fitted probabilities numerically 0 or 1 occurred

## Warning: glm.fit: fitted probabilities numerically 0 or 1 occurred

## Warning: glm.fit: fitted probabilities numerically 0 or 1 occurred

## Warning: glm.fit: fitted probabilities numerically 0 or 1 occurred

## Warning: glm.fit: fitted probabilities numerically 0 or 1 occurred

## Warning: glm.fit: fitted probabilities numerically 0 or 1 occurred

## Warning: glm.fit: fitted probabilities numerically 0 or 1 occurred

## Warning: glm.fit: fitted probabilities numerically 0 or 1 occurred

## Warning: glm.fit: fitted probabilities numerically 0 or 1 occurred

## Warning: glm.fit: fitted probabilities numerically 0 or 1 occurred
```

File failed to load: file:///C:/Users/mitho/Dokumente/B%C3%BCro/DFG-Angst/Paper%20Nathania/Repair_Attachment/manuscript/Review%

```
##
2.5 %
## (Intercept)                                                              -5.82
3101692
## diagnosisClinical                                                        -8.87
4882972
## CPOS_e_CPVC_e_IPOS_INEU_latency_P3                                       -0.85
5873196
## PESI_3rd_trimenon_cor                                                    -0.11
9022736
## diagnosisClinical:CPOS_e_CPVC_e_IPOS_INEU_latency_P3                     -0.11
0413274
## diagnosisClinical:PESI_3rd_trimenon_cor                                  -0.13
5448683
## diagnosisControl:CPOS_e_CPVC_e_IPOS_INEU_latency_P3:PESI_3rd_trimenon_cor  -0.00
6591964
## diagnosisClinical:CPOS_e_CPVC_e_IPOS_INEU_latency_P3:PESI_3rd_trimenon_cor -0.01
5680049
##
97.5 %
## (Intercept)                                                              0.048
394562
## diagnosisClinical                                                        4.180
797687
## CPOS_e_CPVC_e_IPOS_INEU_latency_P3                                       0.139
879763
## PESI_3rd_trimenon_cor                                                    0.170
969146
## diagnosisClinical:CPOS_e_CPVC_e_IPOS_INEU_latency_P3                     1.615
614330
## diagnosisClinical:PESI_3rd_trimenon_cor                                  0.195
642284
## diagnosisControl:CPOS_e_CPVC_e_IPOS_INEU_latency_P3:PESI_3rd_trimenon_cor  0.054
716869
## diagnosisClinical:CPOS_e_CPVC_e_IPOS_INEU_latency_P3:PESI_3rd_trimenon_cor 0.009
351764
```

```
exp(coef(model_2)) # exponentiated coefficients
```

File failed to load: file:///C:/Users/mitho/Dokumente/B%C3%BCro/DFG-Angst/Paper%20Nathania/Repair_Attachment/manuscript/Review%

```
##                                                                  (Intercept) 
##                                                                   0.07541749 
##                                                            diagnosisClinical 
##                                                                   0.37772681 
##                                          CPOS_e_CPVC_e_IPOS_INEU_latency_P3 
##                                                                   0.81692381 
##                                                        PESI_3rd_trimenon_cor 
##                                                                   1.03385716 
##                        diagnosisClinical:CPOS_e_CPVC_e_IPOS_INEU_latency_P3 
##                                                                   1.63692188 
##                                     diagnosisClinical:PESI_3rd_trimenon_cor 
##                                                                   1.01874407 
##  diagnosisControl:CPOS_e_CPVC_e_IPOS_INEU_latency_P3:PESI_3rd_trimenon_cor 
##                                                                   1.01504422 
## diagnosisClinical:CPOS_e_CPVC_e_IPOS_INEU_latency_P3:PESI_3rd_trimenon_cor 
##                                                                   0.99704131
```

```
exp(confint(model_2)) # 95% CI for exponentiated coefficients
```

```
## Waiting for profiling to be done...
```

File failed to load: file:///C:/Users/mitho/Dokumente/B%C3%BCro/DFG-Angst/Paper%20Nathania/Repair_Attachment/manuscript/Review%

```
## Warning: glm.fit: fitted probabilities numerically 0 or 1 occurred

## Warning: glm.fit: fitted probabilities numerically 0 or 1 occurred

## Warning: glm.fit: fitted probabilities numerically 0 or 1 occurred

## Warning: glm.fit: fitted probabilities numerically 0 or 1 occurred

## Warning: glm.fit: fitted probabilities numerically 0 or 1 occurred

## Warning: glm.fit: fitted probabilities numerically 0 or 1 occurred

## Warning: glm.fit: fitted probabilities numerically 0 or 1 occurred

## Warning: glm.fit: fitted probabilities numerically 0 or 1 occurred

## Warning: glm.fit: fitted probabilities numerically 0 or 1 occurred

## Warning: glm.fit: fitted probabilities numerically 0 or 1 occurred

## Warning: glm.fit: fitted probabilities numerically 0 or 1 occurred

## Warning: glm.fit: fitted probabilities numerically 0 or 1 occurred

## Warning: glm.fit: fitted probabilities numerically 0 or 1 occurred

## Warning: glm.fit: fitted probabilities numerically 0 or 1 occurred

## Warning: glm.fit: fitted probabilities numerically 0 or 1 occurred

## Warning: glm.fit: fitted probabilities numerically 0 or 1 occurred

## Warning: glm.fit: fitted probabilities numerically 0 or 1 occurred

## Warning: glm.fit: fitted probabilities numerically 0 or 1 occurred
```

File failed to load: file:///C:/Users/mitho/Dokumente/B%C3%BCro/DFG-Angst/Paper%20Nathania/Repair_Attachment/manuscript/Review%

```
##
2.5 %
## (Intercept)                                                              0.002
958415
## diagnosisClinical                                                        0.000
139858
## CPOS_e_CPVC_e_IPOS_INEU_latency_P3                                       0.424
911997
## PESI_3rd_trimenon_cor                                                    0.887
787616
## diagnosisClinical:CPOS_e_CPVC_e_IPOS_INEU_latency_P3                     0.895
463987
## diagnosisClinical:PESI_3rd_trimenon_cor                                  0.873
323978
## diagnosisControl:CPOS_e_CPVC_e_IPOS_INEU_latency_P3:PESI_3rd_trimenon_cor  0.993
429716
## diagnosisClinical:CPOS_e_CPVC_e_IPOS_INEU_latency_P3:PESI_3rd_trimenon_cor 0.984
442243
##                                                                            9
7.5 %
## (Intercept)                                                               1.04
9585
## diagnosisClinical                                                        65.41
8016
## CPOS_e_CPVC_e_IPOS_INEU_latency_P3                                        1.15
0136
## PESI_3rd_trimenon_cor                                                     1.18
6454
## diagnosisClinical:CPOS_e_CPVC_e_IPOS_INEU_latency_P3                      5.03
0978
## diagnosisClinical:PESI_3rd_trimenon_cor                                   1.21
6092
## diagnosisControl:CPOS_e_CPVC_e_IPOS_INEU_latency_P3:PESI_3rd_trimenon_cor   1.05
6242
## diagnosisClinical:CPOS_e_CPVC_e_IPOS_INEU_latency_P3:PESI_3rd_trimenon_cor  1.00
9396
```

```
anova(model_2, test = "Chisq") # Wald coefficient test
```

File failed to load: file:///C:/Users/mitho/Dokumente/B%C3%BCro/DFG-Angst/Paper%20Nathania/Repair_Attachment/manuscript/Review%

```
## Analysis of Deviance Table
##
## Model: binomial, link: logit
##
## Response: FST_numb
##
## Terms added sequentially (first to last)
##
##
##                                                                 Df Deviance
## NULL
## diagnosis                                                        1   5.3443
## CPOS_e_CPVC_e_IPOS_INEU_latency_P3                               1   4.7806
## PESI_3rd_trimenon_cor                                            1   4.2754
## diagnosis:CPOS_e_CPVC_e_IPOS_INEU_latency_P3                     1   1.6103
## diagnosis:PESI_3rd_trimenon_cor                                  1   0.5917
## diagnosis:CPOS_e_CPVC_e_IPOS_INEU_latency_P3:PESI_3rd_trimenon_cor  2   1.9704
##                                                                 Resid. Df
## NULL                                                                   45
## diagnosis                                                              44
## CPOS_e_CPVC_e_IPOS_INEU_latency_P3                                     43
## PESI_3rd_trimenon_cor                                                  42
## diagnosis:CPOS_e_CPVC_e_IPOS_INEU_latency_P3                           41
## diagnosis:PESI_3rd_trimenon_cor                                        40
## diagnosis:CPOS_e_CPVC_e_IPOS_INEU_latency_P3:PESI_3rd_trimenon_cor       38
##                                                                 Resid. Dev
## NULL                                                                56.534
## diagnosis                                                           51.190
## CPOS_e_CPVC_e_IPOS_INEU_latency_P3                                  46.409
## PESI_3rd_trimenon_cor                                               42.134
## diagnosis:CPOS_e_CPVC_e_IPOS_INEU_latency_P3                        40.524
## diagnosis:PESI_3rd_trimenon_cor                                     39.932
## diagnosis:CPOS_e_CPVC_e_IPOS_INEU_latency_P3:PESI_3rd_trimenon_cor     37.962
##                                                                 Pr(>Chi)
## NULL
## diagnosis                                                        0.02079 *
## CPOS_e_CPVC_e_IPOS_INEU_latency_P3                               0.02878 *
## PESI_3rd_trimenon_cor                                            0.03867 *
## diagnosis:CPOS_e_CPVC_e_IPOS_INEU_latency_P3                     0.20445
## diagnosis:PESI_3rd_trimenon_cor                                  0.44176
## diagnosis:CPOS_e_CPVC_e_IPOS_INEU_latency_P3:PESI_3rd_trimenon_cor  0.37336
## ---
## Signif. codes:  0 '***' 0.001 '**' 0.01 '*' 0.05 '.' 0.1 ' ' 1
```

```
model_3 <- glm(FST_numb~diagnosis+
                  CPOS_e_CPVC_e_IPOS_INEU_latency_P3+
                  PESI_3rd_trimenon_cor+
                  diagnosis:CPOS_e_CPVC_e_IPOS_INEU_latency_P3+
                  diagnosis:CPOS_e_CPVC_e_IPOS_INEU_latency_P3:PESI_3rd_trimenon_co
r,
               family=binomial(link=logit),
               data=data_sub)
summary(model_3) # model summary
```

File failed to load: file:///C:/Users/mitho/Dokumente/B%C3%BCro/DFG-Angst/Paper%20Nathania/Repair_Attachment/manuscript/Review%

```
##
## Call:
## glm(formula = FST_numb ~ diagnosis + CPOS_e_CPVC_e_IPOS_INEU_latency_P3 +
##     PESI_3rd_trimenon_cor + diagnosis:CPOS_e_CPVC_e_IPOS_INEU_latency_P3 +
##     diagnosis:CPOS_e_CPVC_e_IPOS_INEU_latency_P3:PESI_3rd_trimenon_cor,
##     family = binomial(link = logit), data = data_sub)
##
## Deviance Residuals:
##     Min       1Q   Median       3Q      Max
## -1.5114  -0.5476  -0.3851   0.3538   2.4245
##
## Coefficients:
##                                                                          Esti
mate
## (Intercept)                                                             -2.84
4533
## diagnosisClinical                                                       -0.41
8270
## CPOS_e_CPVC_e_IPOS_INEU_latency_P3                                      -0.17
0512
## PESI_3rd_trimenon_cor                                                    0.04
7215
## diagnosisClinical:CPOS_e_CPVC_e_IPOS_INEU_latency_P3                     0.44
2793
## diagnosisControl:CPOS_e_CPVC_e_IPOS_INEU_latency_P3:PESI_3rd_trimenon_cor   0.01
3002
## diagnosisClinical:CPOS_e_CPVC_e_IPOS_INEU_latency_P3:PESI_3rd_trimenon_cor -0.00
2709
##                                                                         Std.
Error
## (Intercept)                                                              0.8
96532
## diagnosisClinical                                                        1.7
78370
## CPOS_e_CPVC_e_IPOS_INEU_latency_P3                                       0.1
78847
## PESI_3rd_trimenon_cor                                                    0.0
33745
## diagnosisClinical:CPOS_e_CPVC_e_IPOS_INEU_latency_P3                     0.3
41620
## diagnosisControl:CPOS_e_CPVC_e_IPOS_INEU_latency_P3:PESI_3rd_trimenon_cor    0.0
10888
## diagnosisClinical:CPOS_e_CPVC_e_IPOS_INEU_latency_P3:PESI_3rd_trimenon_cor   0.0
04394
##                                                                         z val
ue
## (Intercept)                                                              -3.1
73
## diagnosisClinical                                                        -0.2
35
## CPOS_e_CPVC_e_IPOS_INEU_latency_P3                                       -0.9
53
## PESI_3rd_trimenon_cor                                                     1.3
99
```

File failed to load: file:///C:/Users/mitho/Dokumente/B%C3%BCro/DFG-Angst/Paper%20Nathania/Repair_Attachment/manuscript/Review%

```
## diagnosisClinical:CPOS_e_CPVC_e_IPOS_INEU_latency_P3                     1.2
96
## diagnosisControl:CPOS_e_CPVC_e_IPOS_INEU_latency_P3:PESI_3rd_trimenon_cor    1.1
94
## diagnosisClinical:CPOS_e_CPVC_e_IPOS_INEU_latency_P3:PESI_3rd_trimenon_cor  -0.6
17
##                                                                           Pr(>|
z|)
## (Intercept)                                                                0.00
151
## diagnosisClinical                                                          0.81
405
## CPOS_e_CPVC_e_IPOS_INEU_latency_P3                                         0.34
039
## PESI_3rd_trimenon_cor                                                      0.16
177
## diagnosisClinical:CPOS_e_CPVC_e_IPOS_INEU_latency_P3                       0.19
492
## diagnosisControl:CPOS_e_CPVC_e_IPOS_INEU_latency_P3:PESI_3rd_trimenon_cor   0.23
240
## diagnosisClinical:CPOS_e_CPVC_e_IPOS_INEU_latency_P3:PESI_3rd_trimenon_cor  0.53
751
##
## (Intercept)                                                               **
## diagnosisClinical
## CPOS_e_CPVC_e_IPOS_INEU_latency_P3
## PESI_3rd_trimenon_cor
## diagnosisClinical:CPOS_e_CPVC_e_IPOS_INEU_latency_P3
## diagnosisControl:CPOS_e_CPVC_e_IPOS_INEU_latency_P3:PESI_3rd_trimenon_cor
## diagnosisClinical:CPOS_e_CPVC_e_IPOS_INEU_latency_P3:PESI_3rd_trimenon_cor
## ---
## Signif. codes:  0 '***' 0.001 '**' 0.01 '*' 0.05 '.' 0.1 ' ' 1
##
## (Dispersion parameter for binomial family taken to be 1)
##
##     Null deviance: 56.534  on 45  degrees of freedom
## Residual deviance: 38.016  on 39  degrees of freedom
##   (12 Beobachtungen als fehlend gelöscht)
## AIC: 52.016
##
## Number of Fisher Scoring iterations: 7
```

```
confint(model_3) # 95% CI for the coefficients
```

```
## Waiting for profiling to be done...
```

File failed to load: file:///C:/Users/mitho/Dokumente/B%C3%BCro/DFG-Angst/Paper%20Nathania/Repair_Attachment/manuscript/Review%

```
## Warning: glm.fit: fitted probabilities numerically 0 or 1 occurred

## Warning: glm.fit: fitted probabilities numerically 0 or 1 occurred

## Warning: glm.fit: fitted probabilities numerically 0 or 1 occurred

## Warning: glm.fit: fitted probabilities numerically 0 or 1 occurred

## Warning: glm.fit: fitted probabilities numerically 0 or 1 occurred

## Warning: glm.fit: fitted probabilities numerically 0 or 1 occurred

## Warning: glm.fit: fitted probabilities numerically 0 or 1 occurred

## Warning: glm.fit: fitted probabilities numerically 0 or 1 occurred

## Warning: glm.fit: fitted probabilities numerically 0 or 1 occurred

## Warning: glm.fit: fitted probabilities numerically 0 or 1 occurred

## Warning: glm.fit: fitted probabilities numerically 0 or 1 occurred

## Warning: glm.fit: fitted probabilities numerically 0 or 1 occurred

## Warning: glm.fit: fitted probabilities numerically 0 or 1 occurred

## Warning: glm.fit: fitted probabilities numerically 0 or 1 occurred

## Warning: glm.fit: fitted probabilities numerically 0 or 1 occurred

## Warning: glm.fit: fitted probabilities numerically 0 or 1 occurred

## Warning: glm.fit: fitted probabilities numerically 0 or 1 occurred

## Warning: glm.fit: fitted probabilities numerically 0 or 1 occurred
```

File failed to load: file:///C:/Users/mitho/Dokumente/B%C3%BCro/DFG-Angst/Paper%20Nathania/Repair_Attachment/manuscript/Review%

```
##
2.5 %
## (Intercept)                                                                   -4.95
8078317
## diagnosisClinical                                                             -4.53
9183303
## CPOS_e_CPVC_e_IPOS_INEU_latency_P3                                            -0.60
7918406
## PESI_3rd_trimenon_cor                                                         -0.00
9079502
## diagnosisClinical:CPOS_e_CPVC_e_IPOS_INEU_latency_P3                          -0.06
0937691
## diagnosisControl:CPOS_e_CPVC_e_IPOS_INEU_latency_P3:PESI_3rd_trimenon_cor  -0.00
4867507
## diagnosisClinical:CPOS_e_CPVC_e_IPOS_INEU_latency_P3:PESI_3rd_trimenon_cor -0.01
4870278
##
97.5 %
## (Intercept)                                                                   -1.32
7420929
## diagnosisClinical                                                              2.58
0307560
## CPOS_e_CPVC_e_IPOS_INEU_latency_P3                                             0.11
0903947
## PESI_3rd_trimenon_cor                                                          0.12
6660325
## diagnosisClinical:CPOS_e_CPVC_e_IPOS_INEU_latency_P3                           1.43
2996367
## diagnosisControl:CPOS_e_CPVC_e_IPOS_INEU_latency_P3:PESI_3rd_trimenon_cor   0.03
9599669
## diagnosisClinical:CPOS_e_CPVC_e_IPOS_INEU_latency_P3:PESI_3rd_trimenon_cor  0.00
9445476
```

```
exp(coef(model_3)) # exponentiated coefficients
```

```
##                                                                   (Intercept)
##                                                                     0.0581614
##                                                             diagnosisClinical
##                                                                     0.6581847
##                                            CPOS_e_CPVC_e_IPOS_INEU_latency_P3
##                                                                     0.8432333
##                                                         PESI_3rd_trimenon_cor
##                                                                     1.0483473
##                          diagnosisClinical:CPOS_e_CPVC_e_IPOS_INEU_latency_P3
##                                                                     1.5570506
##   diagnosisControl:CPOS_e_CPVC_e_IPOS_INEU_latency_P3:PESI_3rd_trimenon_cor
##                                                                     1.0130870
## diagnosisClinical:CPOS_e_CPVC_e_IPOS_INEU_latency_P3:PESI_3rd_trimenon_cor
##                                                                     0.9972944
```

```
exp(confint(model_3)) # 95% CI for exponentiated coefficients
```

File failed to load: file:///C:/Users/mitho/Dokumente/B%C3%BCro/DFG-Angst/Paper%20Nathania/Repair_Attachment/manuscript/Review%

```
## Waiting for profiling to be done...
```

```
## Warning: glm.fit: fitted probabilities numerically 0 or 1 occurred

## Warning: glm.fit: fitted probabilities numerically 0 or 1 occurred

## Warning: glm.fit: fitted probabilities numerically 0 or 1 occurred

## Warning: glm.fit: fitted probabilities numerically 0 or 1 occurred

## Warning: glm.fit: fitted probabilities numerically 0 or 1 occurred

## Warning: glm.fit: fitted probabilities numerically 0 or 1 occurred

## Warning: glm.fit: fitted probabilities numerically 0 or 1 occurred

## Warning: glm.fit: fitted probabilities numerically 0 or 1 occurred

## Warning: glm.fit: fitted probabilities numerically 0 or 1 occurred

## Warning: glm.fit: fitted probabilities numerically 0 or 1 occurred

## Warning: glm.fit: fitted probabilities numerically 0 or 1 occurred

## Warning: glm.fit: fitted probabilities numerically 0 or 1 occurred

## Warning: glm.fit: fitted probabilities numerically 0 or 1 occurred

## Warning: glm.fit: fitted probabilities numerically 0 or 1 occurred

## Warning: glm.fit: fitted probabilities numerically 0 or 1 occurred

## Warning: glm.fit: fitted probabilities numerically 0 or 1 occurred

## Warning: glm.fit: fitted probabilities numerically 0 or 1 occurred

## Warning: glm.fit: fitted probabilities numerically 0 or 1 occurred
```

File failed to load: file:///C:/Users/mitho/Dokumente/B%C3%BCro/DFG-Angst/Paper%20Nathania/Repair_Attachment/manuscript/Review%

```
##
2.5 %
## (Intercept)                                                                    0.007
026417
## diagnosisClinical                                                              0.010
682127
## CPOS_e_CPVC_e_IPOS_INEU_latency_P3                                             0.544
483083
## PESI_3rd_trimenon_cor                                                          0.990
961592
## diagnosisClinical:CPOS_e_CPVC_e_IPOS_INEU_latency_P3                           0.940
881864
## diagnosisControl:CPOS_e_CPVC_e_IPOS_INEU_latency_P3:PESI_3rd_trimenon_cor  0.995
144320
## diagnosisClinical:CPOS_e_CPVC_e_IPOS_INEU_latency_P3:PESI_3rd_trimenon_cor 0.985
239739
##                                                                                   9
7.5 %
## (Intercept)                                                                     0.26
51602
## diagnosisClinical                                                              13.20
11977
## CPOS_e_CPVC_e_IPOS_INEU_latency_P3                                              1.11
72876
## PESI_3rd_trimenon_cor                                                           1.13
50314
## diagnosisClinical:CPOS_e_CPVC_e_IPOS_INEU_latency_P3                            4.19
12389
## diagnosisControl:CPOS_e_CPVC_e_IPOS_INEU_latency_P3:PESI_3rd_trimenon_cor   1.04
03942
## diagnosisClinical:CPOS_e_CPVC_e_IPOS_INEU_latency_P3:PESI_3rd_trimenon_cor  1.00
94902
```

```
anova(model_3, test = "Chisq") # Wald coefficient test
```

File failed to load: file:///C:/Users/mitho/Dokumente/B%C3%BCro/DFG-Angst/Paper%20Nathania/Repair_Attachment/manuscript/Review%

```
## Analysis of Deviance Table
##
## Model: binomial, link: logit
##
## Response: FST_numb
##
## Terms added sequentially (first to last)
##
##
##                                                          Df Deviance
## NULL
## diagnosis                                                 1   5.3443
## CPOS_e_CPVC_e_IPOS_INEU_latency_P3                        1   4.7806
## PESI_3rd_trimenon_cor                                     1   4.2754
## diagnosis:CPOS_e_CPVC_e_IPOS_INEU_latency_P3              1   1.6103
## diagnosis:CPOS_e_CPVC_e_IPOS_INEU_latency_P3:PESI_3rd_trimenon_cor  2   2.5082
##                                                          Resid. Df
## NULL                                                            45
## diagnosis                                                       44
## CPOS_e_CPVC_e_IPOS_INEU_latency_P3                              43
## PESI_3rd_trimenon_cor                                           42
## diagnosis:CPOS_e_CPVC_e_IPOS_INEU_latency_P3                    41
## diagnosis:CPOS_e_CPVC_e_IPOS_INEU_latency_P3:PESI_3rd_trimenon_cor        39
##                                                          Resid. Dev
## NULL                                                        56.534
## diagnosis                                                   51.190
## CPOS_e_CPVC_e_IPOS_INEU_latency_P3                          46.409
## PESI_3rd_trimenon_cor                                       42.134
## diagnosis:CPOS_e_CPVC_e_IPOS_INEU_latency_P3                40.524
## diagnosis:CPOS_e_CPVC_e_IPOS_INEU_latency_P3:PESI_3rd_trimenon_cor     38.016
##                                                          Pr(>Chi)
## NULL
## diagnosis                                                 0.02079 *
## CPOS_e_CPVC_e_IPOS_INEU_latency_P3                        0.02878 *
## PESI_3rd_trimenon_cor                                     0.03867 *
## diagnosis:CPOS_e_CPVC_e_IPOS_INEU_latency_P3              0.20445
## diagnosis:CPOS_e_CPVC_e_IPOS_INEU_latency_P3:PESI_3rd_trimenon_cor  0.28534
## ---
## Signif. codes:  0 '***' 0.001 '**' 0.01 '*' 0.05 '.' 0.1 ' ' 1
```

```
model_4 <- glm(FST_numb~diagnosis+
                  CPOS_e_CPVC_e_IPOS_INEU_latency_P3+
                  PESI_3rd_trimenon_cor+
                  diagnosis:CPOS_e_CPVC_e_IPOS_INEU_latency_P3,
               family=binomial(link=logit),
               data=data_sub)
summary(model_4) # model summary
```

File failed to load: file:///C:/Users/mitho/Dokumente/B%C3%BCro/DFG-Angst/Paper%20Nathania/Repair_Attachment/manuscript/Review%

```
##
## Call:
## glm(formula = FST_numb ~ diagnosis + CPOS_e_CPVC_e_IPOS_INEU_latency_P3 +
##     PESI_3rd_trimenon_cor + diagnosis:CPOS_e_CPVC_e_IPOS_INEU_latency_P3,
##     family = binomial(link = logit), data = data_sub)
##
## Deviance Residuals:
##     Min       1Q   Median       3Q      Max
## -1.4571  -0.6190  -0.4569   0.4716   2.3022
##
## Coefficients:
##                                                     Estimate Std. Error
## (Intercept)                                         -2.85120    0.84994
## diagnosisClinical                                   -1.29764    1.72705
## CPOS_e_CPVC_e_IPOS_INEU_latency_P3                   0.03173    0.02261
## PESI_3rd_trimenon_cor                                0.06044    0.03148
## diagnosisClinical:CPOS_e_CPVC_e_IPOS_INEU_latency_P3  0.11461    0.13743
##                                                     z value Pr(>|z|)
## (Intercept)                                          -3.355 0.000795 ***
## diagnosisClinical                                    -0.751 0.452437
## CPOS_e_CPVC_e_IPOS_INEU_latency_P3                    1.403 0.160494
## PESI_3rd_trimenon_cor                                 1.920 0.054876 .
## diagnosisClinical:CPOS_e_CPVC_e_IPOS_INEU_latency_P3   0.834 0.404310
## ---
## Signif. codes:  0 '***' 0.001 '**' 0.01 '*' 0.05 '.' 0.1 ' ' 1
##
## (Dispersion parameter for binomial family taken to be 1)
##
##     Null deviance: 56.534  on 45  degrees of freedom
## Residual deviance: 40.524  on 41  degrees of freedom
##   (12 Beobachtungen als fehlend gelöscht)
## AIC: 50.524
##
## Number of Fisher Scoring iterations: 7
```

```
confint(model_4) # 95% CI for the coefficients
```

```
## Waiting for profiling to be done...
```

```
## Warning: glm.fit: fitted probabilities numerically 0 or 1 occurred

## Warning: glm.fit: fitted probabilities numerically 0 or 1 occurred
```

```
##                                                            2.5 %      97.5 %
## (Intercept)                                         -4.822214943 -1.40697517
## diagnosisClinical                                   -5.174136179  1.61873867
## CPOS_e_CPVC_e_IPOS_INEU_latency_P3                  -0.007125265  0.09479259
## PESI_3rd_trimenon_cor                                0.006957091  0.13109693
## diagnosisClinical:CPOS_e_CPVC_e_IPOS_INEU_latency_P3 -0.038810765  0.46202982
```

```
exp(coef(model_4)) # exponentiated coefficients
```

File failed to load: file:///C:/Users/mitho/Dokumente/B%C3%BCro/DFG-Angst/Paper%20Nathania/Repair_Attachment/manuscript/Review%

```
##                                            (Intercept)
##                                             0.05777508
##                                      diagnosisClinical
##                                             0.27317681
##                     CPOS_e_CPVC_e_IPOS_INEU_latency_P3
##                                             1.03223968
##                                PESI_3rd_trimenon_cor
##                                             1.06230601
## diagnosisClinical:CPOS_e_CPVC_e_IPOS_INEU_latency_P3
##                                             1.12143286
```

```
exp(confint(model_4)) # 95% CI for exponentiated coefficients
```

```
## Waiting for profiling to be done...
```

```
## Warning: glm.fit: fitted probabilities numerically 0 or 1 occurred

## Warning: glm.fit: fitted probabilities numerically 0 or 1 occurred
```

```
##                                                            2.5 %    97.5 %
## (Intercept)                                          0.008048939 0.2448829
## diagnosisClinical                                    0.005661105 5.0467207
## CPOS_e_CPVC_e_IPOS_INEU_latency_P3                   0.992900060 1.0994308
## PESI_3rd_trimenon_cor                                1.006981347 1.1400783
## diagnosisClinical:CPOS_e_CPVC_e_IPOS_INEU_latency_P3 0.961932723 1.5872926
```

```
anova(model_4, test = "Chisq") # Wald coefficient test
```

File failed to load: file:///C:/Users/mitho/Dokumente/B%C3%BCro/DFG-Angst/Paper%20Nathania/Repair_Attachment/manuscript/Review%

```
## Analysis of Deviance Table
##
## Model: binomial, link: logit
##
## Response: FST_numb
##
## Terms added sequentially (first to last)
##
##
##                                              Df Deviance Resid. Df Resid. Dev
## NULL                                                           45     56.534
## diagnosis                                     1   5.3443        44     51.190
## CPOS_e_CPVC_e_IPOS_INEU_latency_P3            1   4.7806        43     46.409
## PESI_3rd_trimenon_cor                         1   4.2754        42     42.134
## diagnosis:CPOS_e_CPVC_e_IPOS_INEU_latency_P3  1   1.6103        41     40.524
##                                              Pr(>Chi)
## NULL
## diagnosis                                     0.02079 *
## CPOS_e_CPVC_e_IPOS_INEU_latency_P3            0.02878 *
## PESI_3rd_trimenon_cor                         0.03867 *
## diagnosis:CPOS_e_CPVC_e_IPOS_INEU_latency_P3  0.20445
## ---
## Signif. codes:  0 '***' 0.001 '**' 0.01 '*' 0.05 '.' 0.1 ' ' 1
```

```
model_5 <- glm(FST_numb~diagnosis+
                  CPOS_e_CPVC_e_IPOS_INEU_latency_P3+
                  PESI_3rd_trimenon_cor,
               family=binomial(link=logit),
               data=data_sub)
summary(model_5) # model summary
```

File failed to load: file:///C:/Users/mitho/Dokumente/B%C3%BCro/DFG-Angst/Paper%20Nathania/Repair_Attachment/manuscript/Review%

```
##
## Call:
## glm(formula = FST_numb ~ diagnosis + CPOS_e_CPVC_e_IPOS_INEU_latency_P3 +
##     PESI_3rd_trimenon_cor, family = binomial(link = logit), data = data_sub)
##
## Deviance Residuals:
##     Min       1Q   Median       3Q      Max
## -1.4622  -0.5909  -0.4781   0.6062   2.2616
##
## Coefficients:
##                                    Estimate Std. Error z value Pr(>|z|)
## (Intercept)                        -2.73138    0.78499  -3.480 0.000502 ***
## diagnosisClinical                  -0.18666    1.22687  -0.152 0.879074
## CPOS_e_CPVC_e_IPOS_INEU_latency_P3  0.04581    0.02518   1.820 0.068813 .
## PESI_3rd_trimenon_cor               0.04835    0.02619   1.846 0.064827 .
## ---
## Signif. codes:  0 '***' 0.001 '**' 0.01 '*' 0.05 '.' 0.1 ' ' 1
##
## (Dispersion parameter for binomial family taken to be 1)
##
##     Null deviance: 56.534  on 45  degrees of freedom
## Residual deviance: 42.134  on 42  degrees of freedom
##   (12 Beobachtungen als fehlend gelöscht)
## AIC: 50.134
##
## Number of Fisher Scoring iterations: 5
```

```
confint(model_5) # 95% CI for the coefficients
```

```
## Waiting for profiling to be done...
```

```
##                                           2.5 %     97.5 %
## (Intercept)                        -4.539584751 -1.3814783
## diagnosisClinical                  -2.794980400  2.0871094
## CPOS_e_CPVC_e_IPOS_INEU_latency_P3  0.007193540  0.1103901
## PESI_3rd_trimenon_cor               0.002317745  0.1067220
```

```
exp(coef(model_5)) # exponentiated coefficients
```

```
##                        (Intercept)                  diagnosisClinical
##                         0.06512958                         0.82972614
## CPOS_e_CPVC_e_IPOS_INEU_latency_P3              PESI_3rd_trimenon_cor
##                         1.04688028                         1.04953988
```

```
exp(confint(model_5)) # 95% CI for exponentiated coefficients
```

```
## Waiting for profiling to be done...
```

File failed to load: file:///C:/Users/mitho/Dokumente/B%C3%BCro/DFG-Angst/Paper%20Nathania/Repair_Attachment/manuscript/Review%

```
##                                        2.5 %    97.5 %
## (Intercept)                       0.01067784 0.2512069
## diagnosisClinical                 0.06111607 8.0615786
## CPOS_e_CPVC_e_IPOS_INEU_latency_P3 1.00721948 1.1167136
## PESI_3rd_trimenon_cor             1.00232043 1.1126249
```

```
anova(model_5, test = "Chisq") # Wald coefficient test
```

```
## Analysis of Deviance Table
##
## Model: binomial, link: logit
##
## Response: FST_numb
##
## Terms added sequentially (first to last)
##
##
##                                    Df Deviance Resid. Df Resid. Dev Pr(>Chi)
## NULL                                                  45     56.534
## diagnosis                           1   5.3443        44     51.190  0.02079 *
## CPOS_e_CPVC_e_IPOS_INEU_latency_P3  1   4.7806        43     46.409  0.02878 *
## PESI_3rd_trimenon_cor               1   4.2754        42     42.134  0.03867 *
## ---
## Signif. codes:  0 '***' 0.001 '**' 0.01 '*' 0.05 '.' 0.1 ' ' 1
```

```
model_6 <- glm(FST_numb~diagnosis +
                  CPOS_e_CPVC_e_IPOS_INEU_latency_P3,
               family=binomial(link=logit),
               data=data_sub)
summary(model_6) # model summary
```

File failed to load: file:///C:/Users/mitho/Dokumente/B%C3%BCro/DFG-Angst/Paper%20Nathania/Repair_Attachment/manuscript/Review%

```
##
## Call:
## glm(formula = FST_numb ~ diagnosis + CPOS_e_CPVC_e_IPOS_INEU_latency_P3,
##     family = binomial(link = logit), data = data_sub)
##
## Deviance Residuals:
##     Min       1Q   Median       3Q      Max
## -1.2862  -0.6096  -0.5568   0.8579   1.9566
##
## Coefficients:
##                                    Estimate Std. Error z value Pr(>|z|)
## (Intercept)                        -1.82505    0.52639  -3.467 0.000526 ***
## diagnosisClinical                   1.69486    0.69924   2.424 0.015356 *
## CPOS_e_CPVC_e_IPOS_INEU_latency_P3  0.04102    0.02334   1.757 0.078902 .
## ---
## Signif. codes:  0 '***' 0.001 '**' 0.01 '*' 0.05 '.' 0.1 ' ' 1
##
## (Dispersion parameter for binomial family taken to be 1)
##
##     Null deviance: 61.906  on 48  degrees of freedom
## Residual deviance: 49.947  on 46  degrees of freedom
##   (9 Beobachtungen als fehlend gelöscht)
## AIC: 55.947
##
## Number of Fisher Scoring iterations: 4
```

```
confint(model_6) # 95% CI for the coefficients
```

```
## Waiting for profiling to be done...
```

```
##                                           2.5 %     97.5 %
## (Intercept)                        -2.998029919 -0.8883635
## diagnosisClinical                   0.362812514  3.1426817
## CPOS_e_CPVC_e_IPOS_INEU_latency_P3  0.005250028  0.1043587
```

```
exp(coef(model_6)) # exponentiated coefficients
```

```
##                        (Intercept)                  diagnosisClinical
##                           0.161210                           5.445894
## CPOS_e_CPVC_e_IPOS_INEU_latency_P3
##                           1.041871
```

```
exp(confint(model_6)) # 95% CI for exponentiated coefficients
```

```
## Waiting for profiling to be done...
```

File failed to load: file:///C:/Users/mitho/Dokumente/B%C3%BCro/DFG-Angst/Paper%20Nathania/Repair_Attachment/manuscript/Review%

```
##                                     2.5 %     97.5 %
## (Intercept)                     0.04988525  0.4113284
## diagnosisClinical               1.43736635 23.1659065
## CPOS_e_CPVC_e_IPOS_INEU_latency_P3 1.00526383  1.1099985
```

```
anova(model_6, test = "Chisq") # Wald coefficient test
```

```
## Analysis of Deviance Table
##
## Model: binomial, link: logit
##
## Response: FST_numb
##
## Terms added sequentially (first to last)
##
##
##                                    Df Deviance Resid. Df Resid. Dev Pr(>Chi)
## NULL                                                  48     61.906
## diagnosis                           1   6.7131        47     55.193 0.009571 **
## CPOS_e_CPVC_e_IPOS_INEU_latency_P3  1   5.2463        46     49.947 0.021993 *
## ---
## Signif. codes:  0 '***' 0.001 '**' 0.01 '*' 0.05 '.' 0.1 ' ' 1
```

Survival analysis onto match-event

```
# defining survival object
Surv_obj <- with(data_sub, Surv(match_latency, as.numeric(match_event)))

# Null-model
Surv_1 <- survfit(Surv_obj~1, data=data_sub)
summary(Surv_1)
```

```
## Call: survfit(formula = Surv_obj ~ 1, data = data_sub)
##
##    time n.risk n.event survival std.err lower 95% CI upper 95% CI
##    0.00     58       3    0.948  0.0291       0.8930        1.000
##    0.44     55       1    0.931  0.0333       0.8681        0.999
##    0.48     54       1    0.914  0.0369       0.8443        0.989
##    0.56     53       2    0.879  0.0428       0.7993        0.967
##    0.68     51       2    0.845  0.0475       0.7566        0.943
##    0.72     49       1    0.828  0.0496       0.7359        0.931
##    0.84     48       1    0.810  0.0515       0.7155        0.918
##    0.88     47       1    0.793  0.0532       0.6954        0.905
##    0.96     46       2    0.759  0.0562       0.6561        0.877
##    1.00     44       2    0.724  0.0587       0.6178        0.849
##    1.08     42       1    0.707  0.0598       0.5989        0.834
##    1.12     41       1    0.690  0.0607       0.5803        0.820
##    1.24     40       1    0.672  0.0616       0.5619        0.805
##    1.36     39       1    0.655  0.0624       0.5436        0.790
##    1.48     38       1    0.638  0.0631       0.5255        0.774
##    1.60     37       1    0.621  0.0637       0.5076        0.759
##    1.68     36       1    0.603  0.0642       0.4898        0.743
##    1.72     35       1    0.586  0.0647       0.4722        0.728
##    1.76     34       1    0.569  0.0650       0.4548        0.712
##    1.92     33       1    0.552  0.0653       0.4375        0.696
##    2.36     32       1    0.534  0.0655       0.4204        0.680
##    2.72     31       1    0.517  0.0656       0.4034        0.663
##    2.88     30       1    0.500  0.0657       0.3865        0.647
##    2.92     29       1    0.483  0.0656       0.3699        0.630
##    3.56     28       1    0.466  0.0655       0.3533        0.613
##    3.68     27       1    0.448  0.0653       0.3369        0.596
##    4.04     26       1    0.431  0.0650       0.3207        0.579
##    4.08     25       1    0.414  0.0647       0.3046        0.562
##    4.24     24       1    0.397  0.0642       0.2887        0.545
##    5.32     23       1    0.379  0.0637       0.2729        0.527
##    5.44     22       1    0.362  0.0631       0.2573        0.510
##    5.72     21       1    0.345  0.0624       0.2418        0.492
##    5.76     20       1    0.328  0.0616       0.2266        0.474
##    6.20     19       1    0.310  0.0607       0.2115        0.455
##    8.08     18       1    0.293  0.0598       0.1965        0.437
##   10.28     17       1    0.276  0.0587       0.1818        0.419
##   16.12     16       1    0.259  0.0575       0.1673        0.400
##   16.44     15       1    0.241  0.0562       0.1530        0.381
##   22.92     14       1    0.224  0.0548       0.1389        0.362
##   50.64     13       1    0.207  0.0532       0.1250        0.342
##   58.88     12       1    0.190  0.0515       0.1114        0.323
##   60.44     11       1    0.172  0.0496       0.0981        0.303
##  109.16     10       1    0.155  0.0475       0.0851        0.283
```

```
ggsurvplot(Surv_1, palette = "lightgrey",
           linetype = 1,
           conf.int = TRUE,
           legend.title="",
           legend.labs="all") +
   labs(x = "Latency to interactive repair (sec)", y = "Probability for mismatch")
```

File failed to load: file:///C:/Users/mitho/Dokumente/B%C3%BCro/DFG-Angst/Paper%20Nathania/Repair_Attachment/manuscript/Review%

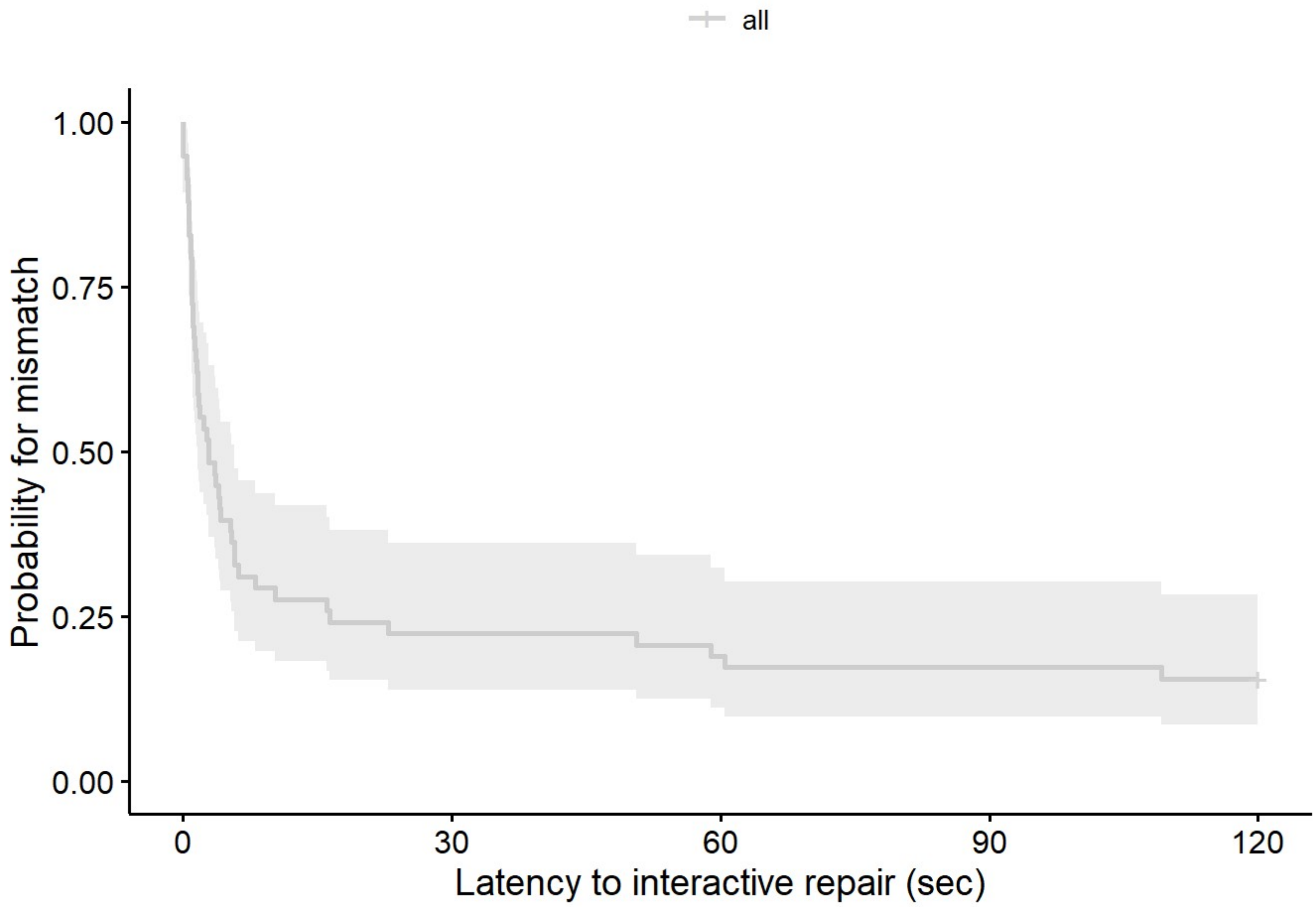


```
Cox_0 <- coxph(Surv_obj~1, data = data_sub, ties = "breslow")
Cox_0
```

```
## Call:  coxph(formula = Surv_obj ~ 1, data = data_sub, ties = "breslow")
##
## Null model
##   log likelihood= -167.7908
##   n= 58
```

```
# Hierarchical Cox-regressions (backwards)
Cox_1 <- coxph(Surv_obj~FST_numb+diagnosis+PESI_3rd_trimenon_cor, data = data_sub)
Cox_1
```

```
## Call:
## coxph(formula = Surv_obj ~ FST_numb + diagnosis + PESI_3rd_trimenon_cor,
##     data = data_sub)
##
##                            coef exp(coef)  se(coef)      z      p
## FST_numbInsecure      -0.713158  0.490094  0.354681 -2.011 0.0444
## diagnosisClinical      0.440809  1.553963  0.475029  0.928 0.3534
## PESI_3rd_trimenon_cor -0.011530  0.988537  0.009518 -1.211 0.2257
##
## Likelihood ratio test=6.2  on 3 df, p=0.1023
## n= 55, number of events= 46
##    (3 Beobachtungen als fehlend gelöscht)
```

File failed to load: file:///C:/Users/mitho/Dokumente/B%C3%BCro/DFG-Angst/Paper%20Nathania/Repair_Attachment/manuscript/Review%

```
Cox_2 <- coxph(Surv_obj~FST_numb+PESI_3rd_trimenon_cor, data = data_sub)
Cox_2
```

```
## Call:
## coxph(formula = Surv_obj ~ FST_numb + PESI_3rd_trimenon_cor,
##     data = data_sub)
##
##                            coef exp(coef)  se(coef)      z      p
## FST_numbInsecure      -0.617579  0.539249  0.335274 -1.842 0.0655
## PESI_3rd_trimenon_cor -0.005313  0.994701  0.006964 -0.763 0.4455
##
## Likelihood ratio test=5.37  on 2 df, p=0.06811
## n= 55, number of events= 46
##    (3 Beobachtungen als fehlend gelöscht)
```

```
Cox_3 <- coxph(Surv_obj~FST_numb, data = data_sub)
Cox_3
```

```
## Call:
## coxph(formula = Surv_obj ~ FST_numb, data = data_sub)
##
##                     coef exp(coef) se(coef)      z      p
## FST_numbInsecure -0.6623    0.5157   0.3082 -2.149 0.0317
##
## Likelihood ratio test=4.9  on 1 df, p=0.02692
## n= 58, number of events= 49
```

```
# Exploring final model
Surv_2 <- survfit(Surv_obj~FST_numb, data=data_sub)
summary(Surv_2)
```

File failed to load: file:///C:/Users/mitho/Dokumente/B%C3%BCro/DFG-Angst/Paper%20Nathania/Repair_Attachment/manuscript/Review%

```
## Call: survfit(formula = Surv_obj ~ FST_numb, data = data_sub)
##
##                 FST_numb=Secure
##   time n.risk n.event survival std.err lower 95% CI upper 95% CI
##   0.00     37       3    0.919  0.0449       0.8350        1.000
##   0.44     34       1    0.892  0.0510       0.7972        0.998
##   0.48     33       1    0.865  0.0562       0.7614        0.982
##   0.56     32       2    0.811  0.0644       0.6939        0.947
##   0.68     30       2    0.757  0.0705       0.6304        0.908
##   0.72     28       1    0.730  0.0730       0.5998        0.888
##   0.84     27       1    0.703  0.0751       0.5698        0.867
##   0.96     26       1    0.676  0.0770       0.5405        0.845
##   1.00     25       1    0.649  0.0785       0.5117        0.822
##   1.12     24       1    0.622  0.0797       0.4834        0.799
##   1.24     23       1    0.595  0.0807       0.4557        0.776
##   1.36     22       1    0.568  0.0814       0.4284        0.752
##   1.48     21       1    0.541  0.0819       0.4016        0.728
##   1.68     20       1    0.514  0.0822       0.3753        0.703
##   1.76     19       1    0.486  0.0822       0.3494        0.677
##   1.92     18       1    0.459  0.0819       0.3239        0.652
##   2.72     17       1    0.432  0.0814       0.2990        0.626
##   2.88     16       1    0.405  0.0807       0.2744        0.599
##   2.92     15       1    0.378  0.0797       0.2504        0.572
##   3.56     14       1    0.351  0.0785       0.2268        0.544
##   3.68     13       1    0.324  0.0770       0.2037        0.516
##   4.08     12       1    0.297  0.0751       0.1812        0.488
##   5.44     11       1    0.270  0.0730       0.1592        0.459
##   5.76     10       1    0.243  0.0705       0.1378        0.429
##   6.20      9       1    0.216  0.0677       0.1171        0.399
##   8.08      8       1    0.189  0.0644       0.0971        0.369
##  10.28      7       1    0.162  0.0606       0.0780        0.337
##  16.44      6       1    0.135  0.0562       0.0598        0.305
##  50.64      5       1    0.108  0.0510       0.0428        0.273
##
##                 FST_numb=Insecure
##    time n.risk n.event survival std.err lower 95% CI upper 95% CI
##    0.88     21       1    0.952  0.0465        0.866        1.000
##    0.96     20       1    0.905  0.0641        0.788        1.000
##    1.00     19       1    0.857  0.0764        0.720        1.000
##    1.08     18       1    0.810  0.0857        0.658        0.996
##    1.60     17       1    0.762  0.0929        0.600        0.968
##    1.72     16       1    0.714  0.0986        0.545        0.936
##    2.36     15       1    0.667  0.1029        0.493        0.902
##    4.04     14       1    0.619  0.1060        0.443        0.866
##    4.24     13       1    0.571  0.1080        0.395        0.828
##    5.32     12       1    0.524  0.1090        0.348        0.788
##    5.72     11       1    0.476  0.1090        0.304        0.746
##   16.12     10       1    0.429  0.1080        0.262        0.702
##   22.92      9       1    0.381  0.1060        0.221        0.657
##   58.88      8       1    0.333  0.1029        0.182        0.610
##   60.44      7       1    0.286  0.0986        0.145        0.562
##  109.16      6       1    0.238  0.0929        0.111        0.512
```

File failed to load: file:///C:/Users/mitho/Dokumente/B%C3%BCro/DFG-Angst/Paper%20Nathania/Repair_Attachment/manuscript/Review%

```
survdiff(Surv_obj~FST_numb, data=data_sub)
```

```
## Call:
## survdiff(formula = Surv_obj ~ FST_numb, data = data_sub)
##
##                      N Observed Expected (O-E)^2/E (O-E)^2/V
## FST_numb=Secure     37       33     25.5      2.22      4.76
## FST_numb=Insecure 21       16     23.5      2.40      4.76
##
##  Chisq= 4.8  on 1 degrees of freedom, p= 0.03
```

```
ggsurvplot(Surv_2, palette = c("lightgreen", "lightblue"),
           linetype = 1,
           conf.int = TRUE,
           legend.title="Attachment",
           legend.labs=c("Secure", "Insecure")) +
   labs(x = "Latency to interactive repair (sec)", y = "Probability for mismatch")
```

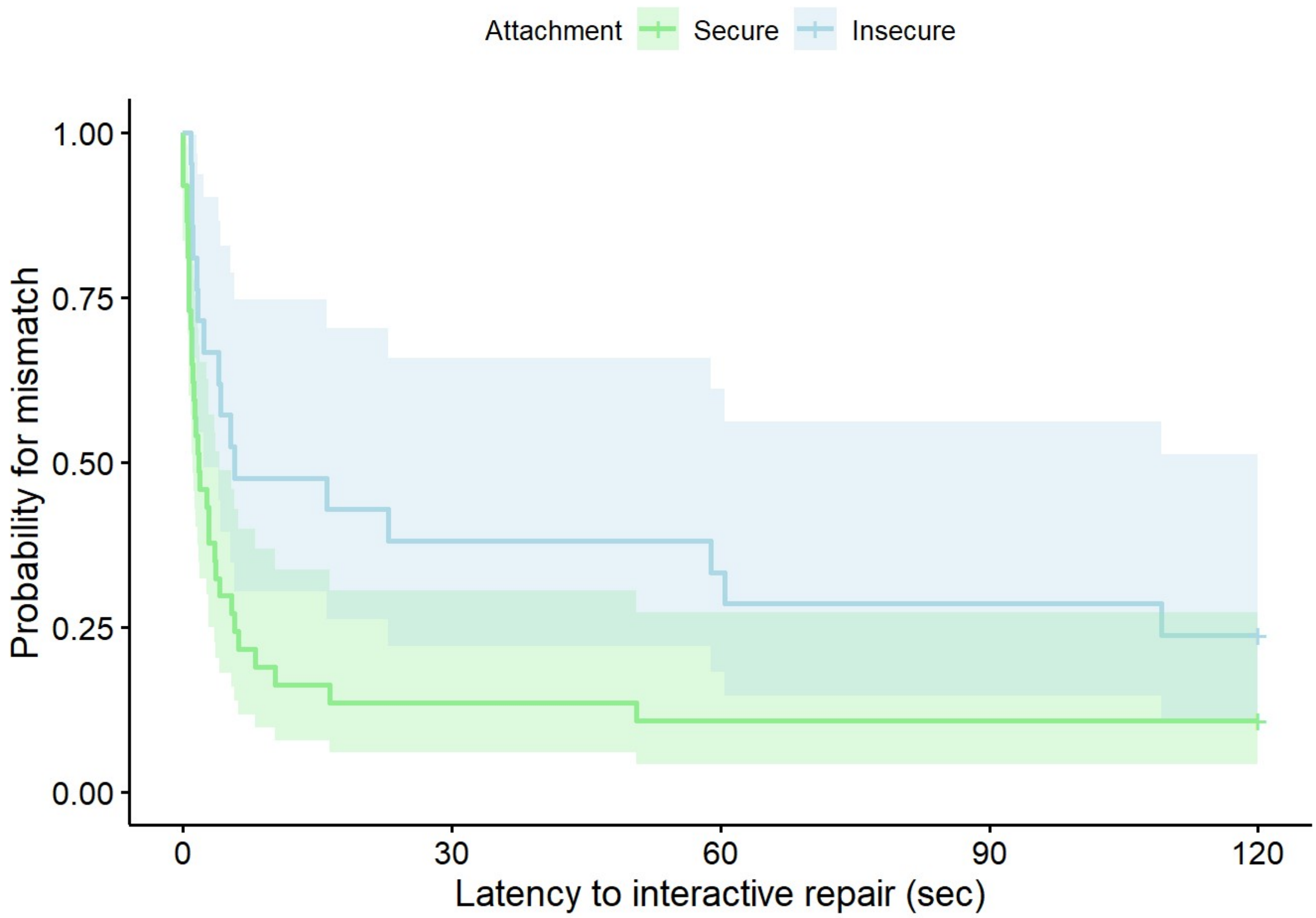

Study 2

Select variables for MCAR-test

File failed to load: file:///C:/Users/mitho/Dokumente/B%C3%BCro/DFG-Angst/Paper%20Nathania/Repair_Attachment/manuscript/Review%

```
data %>%
  select(count_anxiety_diagnoses,
         maternal_education,
         gestation_age,
         APGAR_10,
         PBQ_16_10,
         IBQ_dtn_A1,
         MIB_A1,
         PESI_3rd_trimenon_cor,
         duration_reunion,
         CPOS_e_CPVC_e_IPOS_INEU_latency_P3,
         infant_age_A4,
         MIB_A4,
         child_age_A5_dec,
         maternal_age_BI_A5,
         number_of_children,
         MIB_V_mean,
         CBCL_Internal,
         KABC_SED,
         KABC_SGD,
         SKID_Anxiety_aktuell_count,
         C1_ng_ml,
         C2_ng_ml,
         C3_ng_ml,
         B_mean,
         CIB_P_Sensitivity,
         ) -> data_red_2
View(data_red_2)
```

MCAR-test

```
mcar_test(data_red_2)
```

```
## # A tibble: 1 x 4
##   statistic    df p.value missing.patterns
##       <dbl> <dbl>   <dbl>            <int>
## 1     1188.  1132   0.119               81
```

Exclude cases with missing values

```
data_sub_2 <- data %>%
   filter(is.na(c(FST_numb))==FALSE) %>%
   filter(is.na(c(C1_ng_ml))==FALSE) %>%
   filter(is.na(c(C2_ng_ml))==FALSE) %>%
   filter(is.na(c(C3_ng_ml))==FALSE) %>%
   filter(is.na(c(B_mean))==FALSE)
View(data_sub_2)
```

Descriptives and tests on comparability

File failed to load: file:///C:/Users/mitho/Dokumente/B%C3%BCro/DFG-Angst/Paper%20Nathania/Repair_Attachment/manuscript/Review%

Parametric variables

```
# general means
desc_par_g_2 <- data_sub_2 %>%
   summarise(across(c(maternal_age_BI_A5,
                      child_age_A5_dec,
                      infant_age_A4),
                    list(mean = vm, sd = vsd, se = vse, min = vmin, max = vmax, n =
vnc)))
view(desc_par_g_2)
write_xlsx(desc_par_g_2, "desc_par_g_2.xlsx")

# group-specific means
desc_par_s_2 <- data_sub_2 %>%
   group_by(diagnosis) %>%
   summarise(across(c(maternal_age_BI_A5,
                      child_age_A5_dec,
                      infant_age_A4),
                    list(mean = vm, sd = vsd, se = vse, min = vmin, max = vmax, n =
vnc)))
view(desc_par_s_2)
write_xlsx(desc_par_s_2, "desc_par_s_2.xlsx")

# tests on comparability
data_sub_con_2       <- subset(data_sub_2, data_sub_2$diagnosis == "Control")
data_sub_anx_2       <- subset(data_sub_2, data_sub_2$diagnosis == "Clinical")

t.test(data_sub_con_2$maternal_age_BI_A5,data_sub_anx_2$maternal_age_BI_A5, alterna
tive = "two.sided", var.equal=TRUE, conf.level = 0.95)
```

```
##
##  Two Sample t-test
##
## data:  data_sub_con_2$maternal_age_BI_A5 and data_sub_anx_2$maternal_age_BI_A5
## t = 0.44564, df = 36, p-value = 0.6585
## alternative hypothesis: true difference in means is not equal to 0
## 95 percent confidence interval:
##  -2.873562  4.492023
## sample estimates:
## mean of x mean of y
##  40.04000  39.23077
```

```
t.test(data_sub_con_2$child_age_A5_dec,data_sub_anx_2$child_age_A5_dec, alternative
= "two.sided", var.equal=TRUE, conf.level = 0.95)
```

File failed to load: file:///C:/Users/mitho/Dokumente/B%C3%BCro/DFG-Angst/Paper%20Nathania/Repair_Attachment/manuscript/Review%

```
##
##  Two Sample t-test
##
## data:  data_sub_con_2$child_age_A5_dec and data_sub_anx_2$child_age_A5_dec
## t = -0.88506, df = 36, p-value = 0.382
## alternative hypothesis: true difference in means is not equal to 0
## 95 percent confidence interval:
##  -0.4118557  0.1615993
## sample estimates:
## mean of x mean of y
##  5.663333  5.788462
```

```
t.test(data_sub_con_2$infant_age_A4,data_sub_anx_2$infant_age_A4, alternative = "tw
o.sided", var.equal=TRUE, conf.level = 0.95)
```

```
##
##  Two Sample t-test
##
## data:  data_sub_con_2$infant_age_A4 and data_sub_anx_2$infant_age_A4
## t = -1.1816, df = 26, p-value = 0.2481
## alternative hypothesis: true difference in means is not equal to 0
## 95 percent confidence interval:
##  -1.9439690  0.5248024
## sample estimates:
## mean of x mean of y
##  18.88625  19.59583
```

Ordinal variables

```
# general medians
data_sub_2 %>%
   summarise(maternal_education_med = median(as.numeric(maternal_education)),
             number_of_children_med = median(number_of_children, na.rm = TRUE), SKI
D_Anxiety_aktuell_count_med = median(SKID_Anxiety_aktuell_count, na.rm = TRUE))
```

```
## # A tibble: 1 x 3
##   maternal_education_med number_of_children_med SKID_Anxiety_aktuell_count_med
##                    <dbl>                  <dbl>                          <dbl>
## 1                      4                      2                              0
```

```
# general frequencies
table(data_sub_2$maternal_education)
```

```
##
##       Low secondary qualification      High secondary qualification
##                                 1                                 8
## University entrance qualification                 University degree
##                                 8                                22
```

File failed to load: file:///C:/Users/mitho/Dokumente/B%C3%BCro/DFG-Angst/Paper%20Nathania/Repair_Attachment/manuscript/Review%

```
table(data_sub_2$number_of_children)
```

```
##
##  1  2  3  4  5
##  7 16  8  3  1
```

```
table(data_sub_2$SKID_Anxiety_aktuell_count)
```

```
##
##  0  1
## 31  7
```

```
# group-specific medians
data_sub_2 %>%
   group_by(diagnosis) %>%
   summarise(maternal_education_med = median(as.numeric(maternal_education)),
             number_of_children_med = median(number_of_children, na.rm = TRUE), SKI
D_Anxiety_aktuell_count_med = median(SKID_Anxiety_aktuell_count, na.rm = TRUE))
```

```
## # A tibble: 2 x 4
##   diagnosis maternal_education_med number_of_children_med SKID_Anxiety_aktuell_~
##   <fct>                      <dbl>                  <dbl>                  <dbl>
## 1 Control                        4                      2                      0
## 2 Clinical                       3                      2                      1
```

```
# group-specific frequencies and tests on comparability
table(data_sub_2$maternal_education, data_sub_2$diagnosis)
```

```
##
##                                    Control Clinical
##   Low secondary qualification            1        0
##   High secondary qualification           5        3
##   University entrance qualification      4        4
##   University degree                     16        6
```

```
wilcox.test(as.numeric(maternal_education) ~ diagnosis, data = data_sub_2)
```

```
## Warning in wilcox.test.default(x = c(3, 4, 4, 4, 4, 4, 4, 2, 2, 4, 4, 4, :
## cannot compute exact p-value with ties
```

```
##
##  Wilcoxon rank sum test with continuity correction
##
## data:  as.numeric(maternal_education) by diagnosis
## W = 187.5, p-value = 0.5497
## alternative hypothesis: true location shift is not equal to 0
```

File failed to load: file:///C:/Users/mitho/Dokumente/B%C3%BCro/DFG-Angst/Paper%20Nathania/Repair_Attachment/manuscript/Review%

```
table(data_sub_2$number_of_children, data_sub_2$diagnosis)
```

```
##
##     Control Clinical
##   1       4        3
##   2      11        5
##   3       6        2
##   4       3        0
##   5       1        0
```

```
wilcox.test(amount_infants ~ diagnosis, data = data_sub_2)
```

```
## Warning in wilcox.test.default(x = c(1, 1, 2, 2, 1, 2, 1, 2, 1, 2, 2, 3, :
## cannot compute exact p-value with ties
```

```
##
##  Wilcoxon rank sum test with continuity correction
##
## data:  amount_infants by diagnosis
## W = 221.5, p-value = 0.08845
## alternative hypothesis: true location shift is not equal to 0
```

```
table(data_sub_2$SKID_Anxiety_aktuell_count, data_sub_2$diagnosis)
```

```
##
##     Control Clinical
##   0      25        6
##   1       0        7
```

Nominal variables

```
### Diagnoses

table(data_sub_2$diagnosis)
```

```
##
##  Control Clinical
##       26       13
```

```
table(data_sub_2$SKID_Agora_Panik_aktuell, data_sub_2$diagnosis)
```

```
##
##         Control Clinical
##   False      25       10
##   True        0        3
```

File failed to load: file:///C:/Users/mitho/Dokumente/B%C3%BCro/DFG-Angst/Paper%20Nathania/Repair_Attachment/manuscript/Review%

```r
table(data_sub_2$Skid_Soziale_Phobie_aktuell, data_sub_2$diagnosis)
```

```
##
##         Control Clinical
##   False      25       13
##   True        0        0
```

```r
table(data_sub_2$Skid_Spezifische_Phobie_aktuell, data_sub_2$diagnosis)
```

```
##
##         Control Clinical
##   False      25       13
##   True        0        0
```

```r
table(data_sub_2$Skid_Zwangstörung_aktuell, data_sub_2$diagnosis)
```

```
##
##         Control Clinical
##   False      25       10
##   True        0        3
```

```r
table(data_sub_2$Skid_PTSD_aktuell, data_sub_2$diagnosis)
```

```
##
##         Control Clinical
##   False      25       13
```

```r
table(data_sub_2$Skid_GAS_aktuell, data_sub_2$diagnosis)
```

```
##
##         Control Clinical
##   False      25       12
##   True        0        1
```

```r
table(data_sub_2$Skid_ANNB_aktuell, data_sub_2$diagnosis)
```

```
##
##         Control Clinical
##   False      25       13
```

```r
### General and specific frequencies of sociodemographics & tests on comparability
table(data_sub_2$Berufstätigkeit_aktuell)
```

File failed to load: file:///C:/Users/mitho/Dokumente/B%C3%BCro/DFG-Angst/Paper%20Nathania/Repair_Attachment/manuscript/Review%

```
##
## False  True
##     9    26
```

```
table(data_sub_2$Berufstätigkeit_aktuell, data_sub_2$diagnosis)
```

```
##
##         Control Clinical
##   False       6        3
##   True       19        7
```

```
fisher.test(data_sub_2$Berufstätigkeit_aktuell, data_sub_2$diagnosis)
```

```
##
##  Fisher's Exact Test for Count Data
##
## data:  data_sub_2$Berufstätigkeit_aktuell and data_sub_2$diagnosis
## p-value = 0.6936
## alternative hypothesis: true odds ratio is not equal to 1
## 95 percent confidence interval:
##  0.1145883 5.8534109
## sample estimates:
## odds ratio
##  0.7434909
```

```
table(data_sub_2$Verheiratet_aktuell)
```

```
##
## False  True
##     4    31
```

```
table(data_sub_2$Verheiratet_aktuell, data_sub_2$diagnosis)
```

```
##
##         Control Clinical
##   False       0        4
##   True       25        6
```

```
fisher.test(data_sub_2$Verheiratet_aktuell, data_sub_2$diagnosis)
```

File failed to load: file:///C:/Users/mitho/Dokumente/B%C3%BCro/DFG-Angst/Paper%20Nathania/Repair_Attachment/manuscript/Review%

```
##
##  Fisher's Exact Test for Count Data
##
## data:  data_sub_2$Verheiratet_aktuell and data_sub_2$diagnosis
## p-value = 0.004011
## alternative hypothesis: true odds ratio is not equal to 1
## 95 percent confidence interval:
##  0.0000000 0.4911337
## sample estimates:
## odds ratio
##          0
```

```
table(data_sub_2$infant_sex_index_infant)
```

```
##
## Female   Male
##     26     13
```

```
table(data_sub_2$infant_sex_index_infant, data_sub_2$diagnosis)
```

```
##
##          Control Clinical
##   Female      15       11
##   Male        11        2
```

```
fisher.test(data_sub_2$infant_sex_index_infant, data_sub_2$diagnosis)
```

```
##
##  Fisher's Exact Test for Count Data
##
## data:  data_sub_2$infant_sex_index_infant and data_sub_2$diagnosis
## p-value = 0.1514
## alternative hypothesis: true odds ratio is not equal to 1
## 95 percent confidence interval:
##  0.02310758 1.55234007
## sample estimates:
## odds ratio
##  0.2563287
```

Descriptives Outcomes

File failed to load: file:///C:/Users/mitho/Dokumente/B%C3%BCro/DFG-Angst/Paper%20Nathania/Repair_Attachment/manuscript/Review%

```
# Parametric variables
## General means
desc_par_out_g_2 <- data_sub_2 %>%
   summarise(across(c(C1_ng_ml, C2_ng_ml, C3_ng_ml, B4_ng_ml, B5_ng_ml, B_mean, A5_
Uhrzeit_para,
                      A6_Uhrzeit_para,
                      B4_Uhrzeit_para,
                      B5_Uhrzeit_para,
                      B_Uhrzeit_para_mean),
                    list(mean = vm, sd = vsd, se = vse, min = vmin, max = vmax, n =
vnc)))
View(desc_par_out_g_2)
write_xlsx(desc_par_out_g_2, "desc_par_out_g_2.xlsx")

## Group-specific means
desc_par_out_s_2 <- data_sub_2 %>%
   group_by(diagnosis) %>%
   summarise(across(c(C1_ng_ml, C2_ng_ml, C3_ng_ml, B4_ng_ml, B5_ng_ml, B_mean, A5_
Uhrzeit_para,
                      A6_Uhrzeit_para,
                      B4_Uhrzeit_para,
                      B5_Uhrzeit_para,
                      B_Uhrzeit_para_mean),
                    list(mean = vm, sd = vsd, se = vse, min = vmin, max = vmax, n =
vnc)))
View(desc_par_out_s_2)
write_xlsx(desc_par_out_s_2, "desc_par_out_s_2.xlsx")
```

Confounder Analysis

```
# Pearson correlations between social status and study variables as significantly d
ifferent between groups
cor.test(as.numeric(data_sub_2$Verheiratet_aktuell), data_sub_2$C1_ng_ml)
```

```
##
##  Pearson's product-moment correlation
##
## data:  as.numeric(data_sub_2$Verheiratet_aktuell) and data_sub_2$C1_ng_ml
## t = 0.41522, df = 33, p-value = 0.6807
## alternative hypothesis: true correlation is not equal to 0
## 95 percent confidence interval:
##  -0.2675832  0.3958288
## sample estimates:
##        cor
## 0.07209183
```

```
cor.test(as.numeric(data_sub_2$Verheiratet_aktuell), data_sub_2$C2_ng_ml)
```

File failed to load: file:///C:/Users/mitho/Dokumente/B%C3%BCro/DFG-Angst/Paper%20Nathania/Repair_Attachment/manuscript/Review%

```
##
##  Pearson's product-moment correlation
##
## data:  as.numeric(data_sub_2$Verheiratet_aktuell) and data_sub_2$C2_ng_ml
## t = 0.63921, df = 33, p-value = 0.5271
## alternative hypothesis: true correlation is not equal to 0
## 95 percent confidence interval:
##  -0.2311774  0.4280599
## sample estimates:
##       cor
## 0.1105888
```

```
cor.test(as.numeric(data_sub_2$Verheiratet_aktuell), data_sub_2$C3_ng_ml)
```

```
##
##  Pearson's product-moment correlation
##
## data:  as.numeric(data_sub_2$Verheiratet_aktuell) and data_sub_2$C3_ng_ml
## t = 0.2681, df = 33, p-value = 0.7903
## alternative hypothesis: true correlation is not equal to 0
## 95 percent confidence interval:
##  -0.2911497  0.3740554
## sample estimates:
##        cor
## 0.04662015
```

```
cor.test(as.numeric(data_sub_2$Verheiratet_aktuell), data_sub_2$B4_ng_ml)
```

```
##
##  Pearson's product-moment correlation
##
## data:  as.numeric(data_sub_2$Verheiratet_aktuell) and data_sub_2$B4_ng_ml
## t = 0.62671, df = 33, p-value = 0.5352
## alternative hypothesis: true correlation is not equal to 0
## 95 percent confidence interval:
##  -0.2332230  0.4262923
## sample estimates:
##       cor
## 0.1084526
```

```
cor.test(as.numeric(data_sub_2$Verheiratet_aktuell), data_sub_2$B5_ng_ml)
```

File failed to load: file:///C:/Users/mitho/Dokumente/B%C3%BCro/DFG-Angst/Paper%20Nathania/Repair_Attachment/manuscript/Review%

```
##
##  Pearson's product-moment correlation
##
## data:  as.numeric(data_sub_2$Verheiratet_aktuell) and data_sub_2$B5_ng_ml
## t = 0.91223, df = 33, p-value = 0.3683
## alternative hypothesis: true correlation is not equal to 0
## 95 percent confidence interval:
##  -0.1861406  0.4657393
## sample estimates:
##       cor
## 0.1568345
```

```
cor.test(as.numeric(data_sub_2$Verheiratet_aktuell), data_sub_2$B_mean)
```

```
##
##  Pearson's product-moment correlation
##
## data:  as.numeric(data_sub_2$Verheiratet_aktuell) and data_sub_2$B_mean
## t = 0.77295, df = 33, p-value = 0.4451
## alternative hypothesis: true correlation is not equal to 0
## 95 percent confidence interval:
##  -0.2091908  0.4467456
## sample estimates:
##      cor
## 0.133352
```

```
# Pearson correlations between time of day and cortisol as potential confounder
cor.test(as.numeric(data_sub_2$A5_Uhrzeit_para), data_sub_2$C1_ng_ml)
```

```
##
##  Pearson's product-moment correlation
##
## data:  as.numeric(data_sub_2$A5_Uhrzeit_para) and data_sub_2$C1_ng_ml
## t = 0.10133, df = 36, p-value = 0.9199
## alternative hypothesis: true correlation is not equal to 0
## 95 percent confidence interval:
##  -0.3044414  0.3347616
## sample estimates:
##        cor
## 0.01688532
```

```
cor.test(as.numeric(data_sub_2$A5_Uhrzeit_para), data_sub_2$C2_ng_ml)
```

File failed to load: file:///C:/Users/mitho/Dokumente/B%C3%BCro/DFG-Angst/Paper%20Nathania/Repair_Attachment/manuscript/Review%

```
##
##  Pearson's product-moment correlation
##
## data:  as.numeric(data_sub_2$A5_Uhrzeit_para) and data_sub_2$C2_ng_ml
## t = -0.17085, df = 36, p-value = 0.8653
## alternative hypothesis: true correlation is not equal to 0
## 95 percent confidence interval:
##  -0.3450072  0.2938944
## sample estimates:
##         cor
## -0.02846315
```

```
cor.test(as.numeric(data_sub_2$A5_Uhrzeit_para), data_sub_2$C3_ng_ml)
```

```
##
##  Pearson's product-moment correlation
##
## data:  as.numeric(data_sub_2$A5_Uhrzeit_para) and data_sub_2$C3_ng_ml
## t = -0.31496, df = 36, p-value = 0.7546
## alternative hypothesis: true correlation is not equal to 0
## 95 percent confidence interval:
##  -0.3659714  0.2718175
## sample estimates:
##         cor
## -0.05242099
```

```
cor.test(as.numeric(data_sub_2$A6_Uhrzeit_para), data_sub_2$C1_ng_ml)
```

```
##
##  Pearson's product-moment correlation
##
## data:  as.numeric(data_sub_2$A6_Uhrzeit_para) and data_sub_2$C1_ng_ml
## t = 0.1819, df = 36, p-value = 0.8567
## alternative hypothesis: true correlation is not equal to 0
## 95 percent confidence interval:
##  -0.2922108  0.3466288
## sample estimates:
##        cor
## 0.03030338
```

```
cor.test(as.numeric(data_sub_2$A6_Uhrzeit_para), data_sub_2$C2_ng_ml)
```

File failed to load: file:///C:/Users/mitho/Dokumente/B%C3%BCro/DFG-Angst/Paper%20Nathania/Repair_Attachment/manuscript/Review%

```
##
##  Pearson's product-moment correlation
##
## data:  as.numeric(data_sub_2$A6_Uhrzeit_para) and data_sub_2$C2_ng_ml
## t = 0.29938, df = 36, p-value = 0.7664
## alternative hypothesis: true correlation is not equal to 0
## 95 percent confidence interval:
##  -0.2742176  0.3637232
## sample estimates:
##        cor
## 0.04983439
```

```
cor.test(as.numeric(data_sub_2$A6_Uhrzeit_para), data_sub_2$C3_ng_ml)
```

```
##
##  Pearson's product-moment correlation
##
## data:  as.numeric(data_sub_2$A6_Uhrzeit_para) and data_sub_2$C3_ng_ml
## t = 0.056479, df = 36, p-value = 0.9553
## alternative hypothesis: true correlation is not equal to 0
## 95 percent confidence interval:
##  -0.3112070  0.3281088
## sample estimates:
##         cor
## 0.009412795
```

```
cor.test(data_sub_2$B4_Uhrzeit_para, data_sub_2$B4_ng_ml)
```

```
##
##  Pearson's product-moment correlation
##
## data:  data_sub_2$B4_Uhrzeit_para and data_sub_2$B4_ng_ml
## t = 0.65547, df = 32, p-value = 0.5169
## alternative hypothesis: true correlation is not equal to 0
## 95 percent confidence interval:
##  -0.2320985  0.4362853
## sample estimates:
##       cor
## 0.1151012
```

```
cor.test(data_sub_2$B5_Uhrzeit_para, data_sub_2$B5_ng_ml)
```

File failed to load: file:///C:/Users/mitho/Dokumente/B%C3%BCro/DFG-Angst/Paper%20Nathania/Repair_Attachment/manuscript/Review%

```
##
##  Pearson's product-moment correlation
##
## data:  data_sub_2$B5_Uhrzeit_para and data_sub_2$B5_ng_ml
## t = -0.14993, df = 32, p-value = 0.8818
## alternative hypothesis: true correlation is not equal to 0
## 95 percent confidence interval:
##  -0.3614230  0.3144879
## sample estimates:
##         cor
## -0.02649557
```

```
cor.test(data_sub_2$B_Uhrzeit_para_mean, data_sub_2$B_mean)
```

```
##
##  Pearson's product-moment correlation
##
## data:  data_sub_2$B_Uhrzeit_para_mean and data_sub_2$B_mean
## t = 0.69347, df = 33, p-value = 0.4929
## alternative hypothesis: true correlation is not equal to 0
## 95 percent confidence interval:
##  -0.2222770  0.4356926
## sample estimates:
##      cor
## 0.119847
```

Main analysis: Hierarchichal multi-level-models onto cortisol (backwards)

File failed to load: file:///C:/Users/mitho/Dokumente/B%C3%BCro/DFG-Angst/Paper%20Nathania/Repair_Attachment/manuscript/Review%

```
# pivot data to long format
data_sub_2 %>%
   select(dyade, diagnosis, FST_numb, C1_ng_ml, C2_ng_ml, C3_ng_ml, B_mean) %>%
   rename("1" = C1_ng_ml, "2" = C2_ng_ml, "3" = C3_ng_ml) %>%
   pivot_longer(-c("dyade", "diagnosis", "FST_numb", "B_mean"),
                names_to = "time",
                values_to = "cort_ng_ml") -> data_long
# factorizing variables
data_long$time <- factor(data_long$time, labels = c("immediately before stressor",
"+20 min.", "+40 min."))
data_long$dyade <- factor(data_long$dyade)
View(data_long)

# Model tests
MLM_1 <- lmer(cort_ng_ml ~ diagnosis +
                 FST_numb +
                 time +
                 diagnosis:FST_numb +
                 diagnosis:time +
                 FST_numb:time +
                 diagnosis:FST_numb:time +
                 (1|dyade) +
                 B_mean,
            data=data_long)
summary(MLM_1)
```

File failed to load: file:///C:/Users/mitho/Dokumente/B%C3%BCro/DFG-Angst/Paper%20Nathania/Repair_Attachment/manuscript/Review%

```
## Linear mixed model fit by REML. t-tests use Satterthwaite's method [
## lmerModLmerTest]
## Formula: cort_ng_ml ~ diagnosis + FST_numb + time + diagnosis:FST_numb +
##     diagnosis:time + FST_numb:time + diagnosis:FST_numb:time +
##     (1 | dyade) + B_mean
##    Data: data_long
##
## REML criterion at convergence: 228.9
##
## Scaled residuals:
##     Min      1Q  Median      3Q     Max
## -2.0095 -0.4655 -0.1001  0.3046  4.4533
##
## Random effects:
##  Groups   Name        Variance Std.Dev.
##  dyade    (Intercept) 0.1727   0.4156
##  Residual             0.2866   0.5354
## Number of obs: 117, groups:  dyade, 39
##
## Fixed effects:
##                                                  Estimate Std. Error      df
## (Intercept)                                        0.9466     0.1718 62.9515
## diagnosisClinical                                  0.2508     0.3123 79.9374
## FST_numbInsecure                                   0.5934     0.3693 79.6878
## time+20 min.                                      -0.2800     0.1614 70.0000
## time+40 min.                                      -0.3186     0.1614 70.0000
## B_mean                                             0.4338     0.1226 34.0000
## diagnosisClinical:FST_numbInsecure                -0.2907     0.5272 79.9577
## diagnosisClinical:time+20 min.                     0.3400     0.3487 70.0000
## diagnosisClinical:time+40 min.                    -0.0297     0.3487 70.0000
## FST_numbInsecure:time+20 min.                      0.4475     0.4115 70.0000
## FST_numbInsecure:time+40 min.                     -0.4089     0.4115 70.0000
## diagnosisClinical:FST_numbInsecure:time+20 min.  -0.7218     0.5889 70.0000
## diagnosisClinical:FST_numbInsecure:time+40 min.   0.1758     0.5889 70.0000
##                                                  t value Pr(>|t|)
## (Intercept)                                        5.510  7.1e-07 ***
## diagnosisClinical                                  0.803  0.42427
## FST_numbInsecure                                   1.607  0.11202
## time+20 min.                                      -1.735  0.08721 .
## time+40 min.                                      -1.974  0.05233 .
## B_mean                                             3.539  0.00119 **
## diagnosisClinical:FST_numbInsecure                -0.551  0.58297
## diagnosisClinical:time+20 min.                     0.975  0.33290
## diagnosisClinical:time+40 min.                    -0.085  0.93237
## FST_numbInsecure:time+20 min.                      1.087  0.28060
## FST_numbInsecure:time+40 min.                     -0.993  0.32389
## diagnosisClinical:FST_numbInsecure:time+20 min.  -1.226  0.22443
## diagnosisClinical:FST_numbInsecure:time+40 min.   0.298  0.76623
## ---
## Signif. codes:  0 '***' 0.001 '**' 0.01 '*' 0.05 '.' 0.1 ' ' 1
```

File failed to load: file:///C:/Users/mitho/Dokumente/B%C3%BCro/DFG-Angst/Paper%20Nathania/Repair_Attachment/manuscript/Review%

```
##
## Correlation matrix not shown by default, as p = 13 > 12.
## Use print(x, correlation=TRUE)  or
##     vcov(x)        if you need it
```

```
anova(MLM_1)
```

```
## Type III Analysis of Variance Table with Satterthwaite's method
##                         Sum Sq Mean Sq NumDF DenDF F value   Pr(>F)
## diagnosis               0.0982  0.0982     1    34  0.3425 0.562273
## FST_numb                0.9569  0.9569     1    34  3.3387 0.076456 .
## time                    3.7998  1.8999     2    70  6.6287 0.002310 **
## B_mean                  3.5895  3.5895     1    34 12.5236 0.001187 **
## diagnosis:FST_numb      0.3943  0.3943     1    34  1.3759 0.248961
## diagnosis:time          0.0222  0.0111     2    70  0.0387 0.962022
## FST_numb:time           0.6097  0.3049     2    70  1.0637 0.350703
## diagnosis:FST_numb:time 0.7479  0.3740     2    70  1.3048 0.277749
## ---
## Signif. codes:  0 '***' 0.001 '**' 0.01 '*' 0.05 '.' 0.1 ' ' 1
```

```
MLM_2 <- lmer(cort_ng_ml ~ diagnosis +
                 FST_numb +
                 time +
                 diagnosis:FST_numb +
                 FST_numb:time +
                 diagnosis:FST_numb:time +
                 (1|dyade) +
                 B_mean,
            data=data_long)
summary(MLM_2)
```

File failed to load: file:///C:/Users/mitho/Dokumente/B%C3%BCro/DFG-Angst/Paper%20Nathania/Repair_Attachment/manuscript/Review%

```
## Linear mixed model fit by REML. t-tests use Satterthwaite's method [
## lmerModLmerTest]
## Formula: cort_ng_ml ~ diagnosis + FST_numb + time + diagnosis:FST_numb +
##     FST_numb:time + diagnosis:FST_numb:time + (1 | dyade) + B_mean
##    Data: data_long
##
## REML criterion at convergence: 228.9
##
## Scaled residuals:
##     Min      1Q  Median      3Q     Max
## -2.0095 -0.4655 -0.1001  0.3046  4.4533
##
## Random effects:
##  Groups   Name        Variance Std.Dev.
##  dyade    (Intercept) 0.1727   0.4156
##  Residual             0.2866   0.5354
## Number of obs: 117, groups:  dyade, 39
##
## Fixed effects:
##                                                 Estimate Std. Error      df
## (Intercept)                                       0.9466     0.1718 62.9515
## diagnosisClinical                                 0.2508     0.3123 79.9374
## FST_numbInsecure                                  0.5934     0.3693 79.6878
## time+20 min.                                     -0.2800     0.1614 70.0000
## time+40 min.                                     -0.3186     0.1614 70.0000
## B_mean                                            0.4338     0.1226 34.0000
## diagnosisClinical:FST_numbInsecure               -0.2907     0.5272 79.9577
## FST_numbInsecure:time+20 min.                     0.4475     0.4115 70.0000
## FST_numbInsecure:time+40 min.                    -0.4089     0.4115 70.0000
## diagnosisClinical:FST_numbSecure:time+20 min.     0.3400     0.3487 70.0000
## diagnosisClinical:FST_numbInsecure:time+20 min.  -0.3818     0.4746 70.0000
## diagnosisClinical:FST_numbSecure:time+40 min.    -0.0297     0.3487 70.0000
## diagnosisClinical:FST_numbInsecure:time+40 min.   0.1461     0.4746 70.0000
##                                                 t value Pr(>|t|)
## (Intercept)                                       5.510  7.1e-07 ***
## diagnosisClinical                                 0.803  0.42427
## FST_numbInsecure                                  1.607  0.11202
## time+20 min.                                     -1.735  0.08721 .
## time+40 min.                                     -1.974  0.05233 .
## B_mean                                            3.539  0.00119 **
## diagnosisClinical:FST_numbInsecure               -0.551  0.58297
## FST_numbInsecure:time+20 min.                     1.087  0.28060
## FST_numbInsecure:time+40 min.                    -0.993  0.32389
## diagnosisClinical:FST_numbSecure:time+20 min.     0.975  0.33290
## diagnosisClinical:FST_numbInsecure:time+20 min.  -0.805  0.42382
## diagnosisClinical:FST_numbSecure:time+40 min.    -0.085  0.93237
## diagnosisClinical:FST_numbInsecure:time+40 min.   0.308  0.75914
## ---
## Signif. codes:  0 '***' 0.001 '**' 0.01 '*' 0.05 '.' 0.1 ' ' 1
```

File failed to load: file:///C:/Users/mitho/Dokumente/B%C3%BCro/DFG-Angst/Paper%20Nathania/Repair_Attachment/manuscript/Review%

```
##
## Correlation matrix not shown by default, as p = 13 > 12.
## Use print(x, correlation=TRUE)  or
##     vcov(x)        if you need it
```

```
anova(MLM_2)
```

```
## Type III Analysis of Variance Table with Satterthwaite's method
##                         Sum Sq Mean Sq NumDF  DenDF F value   Pr(>F)
## diagnosis               0.0982  0.0982     1 34.000  0.3425 0.562272
## FST_numb                1.3364  1.3364     1 49.611  4.6626 0.035687 *
## time                    3.7998  1.8999     2 70.000  6.6287 0.002310 **
## B_mean                  3.5895  3.5895     1 34.000 12.5236 0.001187 **
## diagnosis:FST_numb      0.0871  0.0871     1 79.958  0.3039 0.582973
## FST_numb:time           1.2419  0.6210     2 70.000  2.1665 0.122203
## diagnosis:FST_numb:time 0.7760  0.1940     4 70.000  0.6769 0.610267
## ---
## Signif. codes:  0 '***' 0.001 '**' 0.01 '*' 0.05 '.' 0.1 ' ' 1
```

```
MLM_3 <- lmer(cort_ng_ml ~ diagnosis +
                 FST_numb +
                 time +
                 diagnosis:FST_numb +
                 FST_numb:time +
                 (1|dyade) +
                 B_mean,
            data=data_long)
summary(MLM_3)
```

File failed to load: file:///C:/Users/mitho/Dokumente/B%C3%BCro/DFG-Angst/Paper%20Nathania/Repair_Attachment/manuscript/Review%

```
## Linear mixed model fit by REML. t-tests use Satterthwaite's method [
## lmerModLmerTest]
## Formula: cort_ng_ml ~ diagnosis + FST_numb + time + diagnosis:FST_numb +
##     FST_numb:time + (1 | dyade) + B_mean
##    Data: data_long
##
## REML criterion at convergence: 231.2
##
## Scaled residuals:
##     Min      1Q  Median      3Q     Max
## -2.1319 -0.4292 -0.1239  0.2801  4.5255
##
## Random effects:
##  Groups   Name        Variance Std.Dev.
##  dyade    (Intercept) 0.1744   0.4176
##  Residual             0.2816   0.5307
## Number of obs: 117, groups:  dyade, 39
##
## Fixed effects:
##                                   Estimate Std. Error      df t value Pr(>|t|)
## (Intercept)                         0.9244     0.1659 56.7138   5.571 7.26e-07
## diagnosisClinical                   0.3542     0.2387 34.0000   1.484  0.14700
## FST_numbInsecure                    0.6656     0.3220 54.9997   2.067  0.04345
## time+20 min.                       -0.2071     0.1418 74.0000  -1.461  0.14838
## time+40 min.                       -0.3250     0.1418 74.0000  -2.292  0.02478
## B_mean                              0.4338     0.1226 34.0000   3.539  0.00119
## diagnosisClinical:FST_numbInsecure  -0.4727     0.4030 34.0000  -1.173  0.24896
## FST_numbInsecure:time+20 min.        0.1317     0.2671 74.0000   0.493  0.62339
## FST_numbInsecure:time+40 min.       -0.3095     0.2671 74.0000  -1.159  0.25014
##
## (Intercept)                       ***
## diagnosisClinical
## FST_numbInsecure                  *
## time+20 min.
## time+40 min.                      *
## B_mean                            **
## diagnosisClinical:FST_numbInsecure
## FST_numbInsecure:time+20 min.
## FST_numbInsecure:time+40 min.
## ---
## Signif. codes:  0 '***' 0.001 '**' 0.01 '*' 0.05 '.' 0.1 ' ' 1
##
## Correlation of Fixed Effects:
##             (Intr) dgnssC FST_nI t+20m. t+40m. B_mean dC:FST FST_I:+2m
## dignssClncl -0.327
## FST_nmbInsc -0.399  0.161
## time+20min. -0.427  0.000  0.220
## time+40min. -0.427  0.000  0.220  0.500
## B_mean      -0.560  0.035  0.080  0.000  0.000
## dgnsC:FST_I  0.197 -0.592 -0.613  0.000  0.000 -0.025
## FST_I:+20m.  0.227  0.000 -0.415 -0.531 -0.266  0.000  0.000
## FST_I:+40m.  0.227  0.000 -0.415 -0.266 -0.531  0.000  0.000  0.500
```

File failed to load: file:///C:/Users/mitho/Dokumente/B%C3%BCro/DFG-Angst/Paper%20Nathania/Repair_Attachment/manuscript/Review%

```
anova(MLM_3)
```

```
## Type III Analysis of Variance Table with Satterthwaite's method
##                    Sum Sq Mean Sq NumDF DenDF F value   Pr(>F)
## diagnosis          0.0964  0.0964     1    34  0.3425 0.562272
## FST_numb           0.9402  0.9402     1    34  3.3387 0.076456 .
## time               3.8404  1.9202     2    74  6.8185 0.001915 **
## B_mean             3.5268  3.5268     1    34 12.5236 0.001187 **
## diagnosis:FST_numb 0.3875  0.3875     1    34  1.3759 0.248961
## FST_numb:time      0.8104  0.4052     2    74  1.4389 0.243757
## ---
## Signif. codes:  0 '***' 0.001 '**' 0.01 '*' 0.05 '.' 0.1 ' ' 1
```

```
MLM_4 <- lmer(cort_ng_ml ~ FST_numb +
                 time +
                 diagnosis:FST_numb +
                 FST_numb:time +
                 (1|dyade) +
                 B_mean,
             data=data_long)
summary(MLM_4)
```

File failed to load: file:///C:/Users/mitho/Dokumente/B%C3%BCro/DFG-Angst/Paper%20Nathania/Repair_Attachment/manuscript/Review%

```
## Linear mixed model fit by REML. t-tests use Satterthwaite's method [
## lmerModLmerTest]
## Formula: cort_ng_ml ~ FST_numb + time + diagnosis:FST_numb + FST_numb:time +
##     (1 | dyade) + B_mean
##    Data: data_long
##
## REML criterion at convergence: 231.2
##
## Scaled residuals:
##     Min      1Q  Median      3Q     Max
## -2.1319 -0.4292 -0.1239  0.2801  4.5255
##
## Random effects:
##  Groups   Name        Variance Std.Dev.
##  dyade    (Intercept) 0.1744   0.4176
##  Residual             0.2816   0.5307
## Number of obs: 117, groups:  dyade, 39
##
## Fixed effects:
##                                   Estimate Std. Error      df t value Pr(>|t|)
## (Intercept)                         0.9244     0.1659 56.7138   5.571 7.26e-07
## FST_numbInsecure                    0.6656     0.3220 54.9997   2.067  0.04345
## time+20 min.                       -0.2071     0.1418 74.0000  -1.461  0.14838
## time+40 min.                       -0.3250     0.1418 74.0000  -2.292  0.02478
## B_mean                              0.4338     0.1226 34.0000   3.539  0.00119
## FST_numbSecure:diagnosisClinical     0.3542     0.2387 34.0000   1.484  0.14700
## FST_numbInsecure:diagnosisClinical  -0.1184     0.3246 34.0000  -0.365  0.71747
## FST_numbInsecure:time+20 min.        0.1317     0.2671 74.0000   0.493  0.62339
## FST_numbInsecure:time+40 min.       -0.3095     0.2671 74.0000  -1.159  0.25014
##
## (Intercept)                       ***
## FST_numbInsecure                  *
## time+20 min.
## time+40 min.                      *
## B_mean                            **
## FST_numbSecure:diagnosisClinical
## FST_numbInsecure:diagnosisClinical
## FST_numbInsecure:time+20 min.
## FST_numbInsecure:time+40 min.
## ---
## Signif. codes:  0 '***' 0.001 '**' 0.01 '*' 0.05 '.' 0.1 ' ' 1
##
## Correlation of Fixed Effects:
##             (Intr) FST_nI t+20m. t+40m. B_mean FST_S: FST_I: FST_I:+2m
## FST_nmbInsc -0.399
## time+20min. -0.427  0.220
## time+40min. -0.427  0.220  0.500
## B_mean      -0.560  0.080  0.000  0.000
## FST_nmbSc:C -0.327  0.161  0.000  0.000  0.035
## FST_nmbIn:C  0.003 -0.642  0.000  0.000 -0.006  0.000
## FST_I:+20m.  0.227 -0.415 -0.531 -0.266  0.000  0.000  0.000
## FST_I:+40m.  0.227 -0.415 -0.266 -0.531  0.000  0.000  0.000  0.500
```

File failed to load: file:///C:/Users/mitho/Dokumente/B%C3%BCro/DFG-Angst/Paper%20Nathania/Repair_Attachment/manuscript/Review%

```
anova(MLM_4)
```

```
## Type III Analysis of Variance Table with Satterthwaite's method
##                    Sum Sq Mean Sq NumDF DenDF F value   Pr(>F)
## FST_numb           1.2954  1.2954     1    34  4.5998 0.039208 *
## time               3.8404  1.9202     2    74  6.8185 0.001915 **
## B_mean             3.5268  3.5268     1    34 12.5236 0.001187 **
## FST_numb:diagnosis 0.6577  0.3288     2    34  1.1677 0.323247
## FST_numb:time      0.8104  0.4052     2    74  1.4389 0.243757
## ---
## Signif. codes:  0 '***' 0.001 '**' 0.01 '*' 0.05 '.' 0.1 ' ' 1
```

```
MLM_5 <- lmer(cort_ng_ml ~ FST_numb +
                 time +
                 FST_numb:time +
                 (1|dyade) +
                 B_mean,
            data=data_long)
summary(MLM_5)
```

File failed to load: file:///C:/Users/mitho/Dokumente/B%C3%BCro/DFG-Angst/Paper%20Nathania/Repair_Attachment/manuscript/Review%

```
## Linear mixed model fit by REML. t-tests use Satterthwaite's method [
## lmerModLmerTest]
## Formula: cort_ng_ml ~ FST_numb + time + FST_numb:time + (1 | dyade) +
##     B_mean
##    Data: data_long
##
## REML criterion at convergence: 232.1
##
## Scaled residuals:
##     Min      1Q  Median      3Q     Max
## -2.1736 -0.4282 -0.1107  0.2914  4.4837
##
## Random effects:
##  Groups   Name        Variance Std.Dev.
##  dyade    (Intercept) 0.1769   0.4206
##  Residual             0.2816   0.5307
## Number of obs: 117, groups:  dyade, 39
##
## Fixed effects:
##                               Estimate Std. Error      df t value Pr(>|t|)
## (Intercept)                     1.0053     0.1573 63.4656   6.390 2.22e-08 ***
## FST_numbInsecure                0.5131     0.2420 83.1078   2.120  0.03700 *
## time+20 min.                   -0.2071     0.1418 74.0000  -1.461  0.14838
## time+40 min.                   -0.3250     0.1418 74.0000  -2.292  0.02478 *
## B_mean                          0.4271     0.1231 36.0000   3.471  0.00137 **
## FST_numbInsecure:time+20 min.   0.1317     0.2671 74.0000   0.493  0.62339
## FST_numbInsecure:time+40 min.  -0.3095     0.2671 74.0000  -1.159  0.25014
## ---
## Signif. codes:  0 '***' 0.001 '**' 0.01 '*' 0.05 '.' 0.1 ' ' 1
##
## Correlation of Fixed Effects:
##             (Intr) FST_nI t+20m. t+40m. B_mean FST_I:+2m
## FST_nmbInsc -0.485
## time+20min. -0.451  0.293
## time+40min. -0.451  0.293  0.500
## B_mean      -0.582  0.095  0.000  0.000
## FST_I:+20m.  0.239 -0.552 -0.531 -0.266  0.000
## FST_I:+40m.  0.239 -0.552 -0.266 -0.531  0.000  0.500
```

```
anova(MLM_5)
```

```
## Type III Analysis of Variance Table with Satterthwaite's method
##               Sum Sq Mean Sq NumDF DenDF F value   Pr(>F)
## FST_numb      1.6661  1.6661     1    36  5.9163 0.020102 *
## time          3.8404  1.9202     2    74  6.8185 0.001915 **
## B_mean        3.3926  3.3926     1    36 12.0469 0.001365 **
## FST_numb:time 0.8104  0.4052     2    74  1.4389 0.243757
## ---
## Signif. codes:  0 '***' 0.001 '**' 0.01 '*' 0.05 '.' 0.1 ' ' 1
```

File failed to load: file:///C:/Users/mitho/Dokumente/B%C3%BCro/DFG-Angst/Paper%20Nathania/Repair_Attachment/manuscript/Review%

```
MLM_6 <- lmer(cort_ng_ml ~ FST_numb +
                 time +
                 (1|dyade) +
                 B_mean,
              data=data_long)
summary(MLM_6)
```

```
## Linear mixed model fit by REML. t-tests use Satterthwaite's method [
## lmerModLmerTest]
## Formula: cort_ng_ml ~ FST_numb + time + (1 | dyade) + B_mean
##    Data: data_long
##
## REML criterion at convergence: 233.1
##
## Scaled residuals:
##     Min      1Q  Median      3Q     Max
## -2.2568 -0.4454 -0.1556  0.2980  4.4320
##
## Random effects:
##  Groups   Name        Variance Std.Dev.
##  dyade    (Intercept) 0.1758   0.4193
##  Residual             0.2849   0.5337
## Number of obs: 117, groups:  dyade, 39
##
## Fixed effects:
##                  Estimate Std. Error      df t value Pr(>|t|)
## (Intercept)        1.0220     0.1514 56.1186   6.752 8.81e-09 ***
## FST_numbInsecure   0.4538     0.1866 36.0000   2.432  0.02010 *
## time+20 min.      -0.1700     0.1209 76.0000  -1.407  0.16365
## time+40 min.      -0.4123     0.1209 76.0000  -3.411  0.00104 **
## B_mean             0.4271     0.1231 36.0000   3.471  0.00137 **
## ---
## Signif. codes:  0 '***' 0.001 '**' 0.01 '*' 0.05 '.' 0.1 ' ' 1
##
## Correlation of Fixed Effects:
##             (Intr) FST_nI t+20m. t+40m.
## FST_nmbInsc -0.417
## time+20min. -0.399  0.000
## time+40min. -0.399  0.000  0.500
## B_mean      -0.605  0.123  0.000  0.000
```

```
anova(MLM_6)
```

```
## Type III Analysis of Variance Table with Satterthwaite's method
##          Sum Sq Mean Sq NumDF DenDF F value   Pr(>F)
## FST_numb 1.6853  1.6853     1    36  5.9163 0.020102 *
## time     3.3489  1.6745     2    76  5.8781 0.004230 **
## B_mean   3.4317  3.4317     1    36 12.0469 0.001365 **
## ---
## Signif. codes:  0 '***' 0.001 '**' 0.01 '*' 0.05 '.' 0.1 ' ' 1
```

File failed to load: file:///C:/Users/mitho/Dokumente/B%C3%BCro/DFG-Angst/Paper%20Nathania/Repair_Attachment/manuscript/Review%

```
# Plotting
## main effect group
data_long %>%
   ggplot(aes(x = FST_numb,
              y = cort_ng_ml)) +
   geom_boxplot() +
   labs(x = "Attachment", y = "Cortisol (ng/ml)")
```

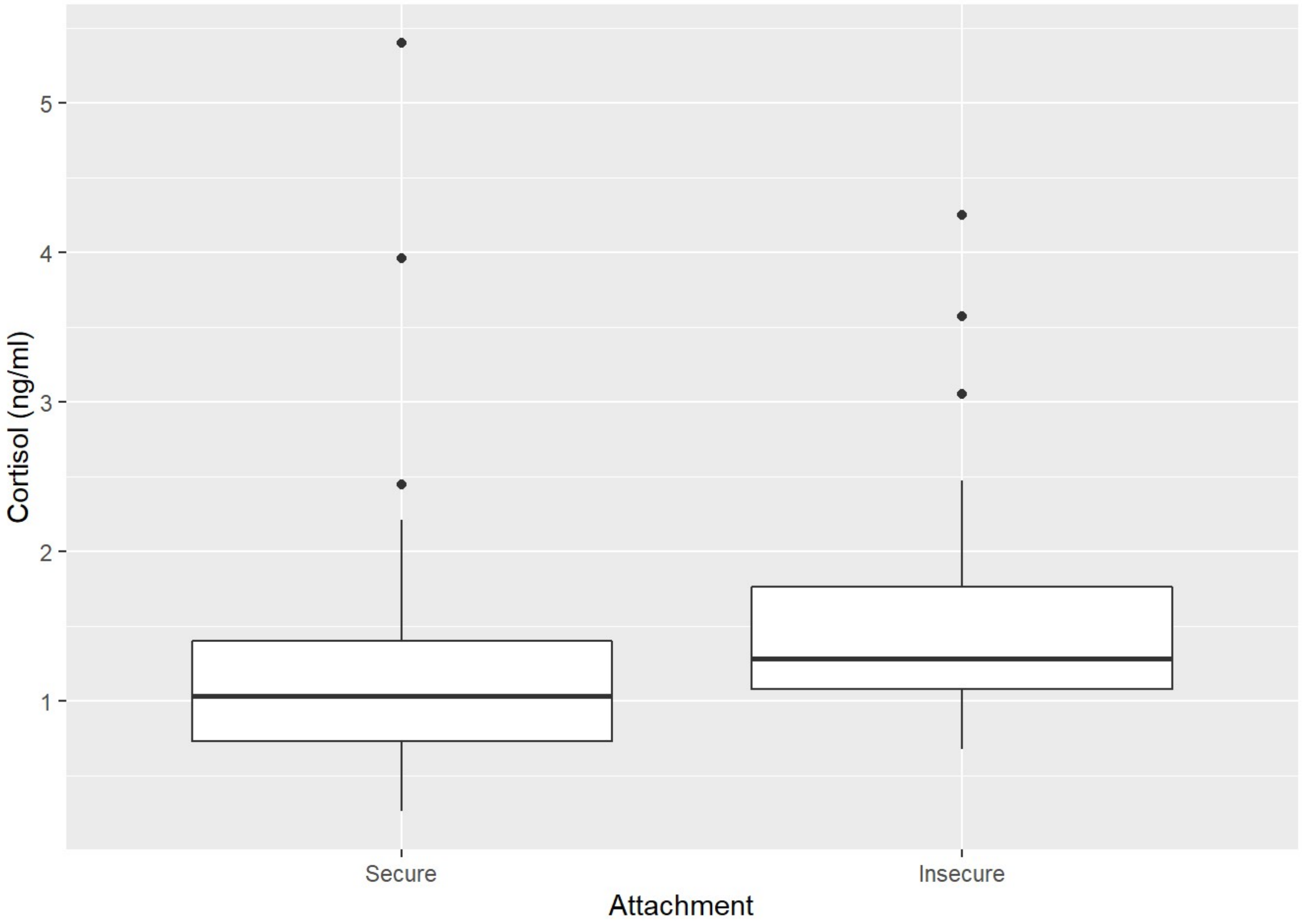


```
## main effect time
data_long %>%
   ggplot(aes(x = time,
              y = cort_ng_ml)) +
   geom_boxplot() +
   labs(x = "Measurement time", y = "Cortisol (ng/ml)")
```

File failed to load: file:///C:/Users/mitho/Dokumente/B%C3%BCro/DFG-Angst/Paper%20Nathania/Repair_Attachment/manuscript/Review%

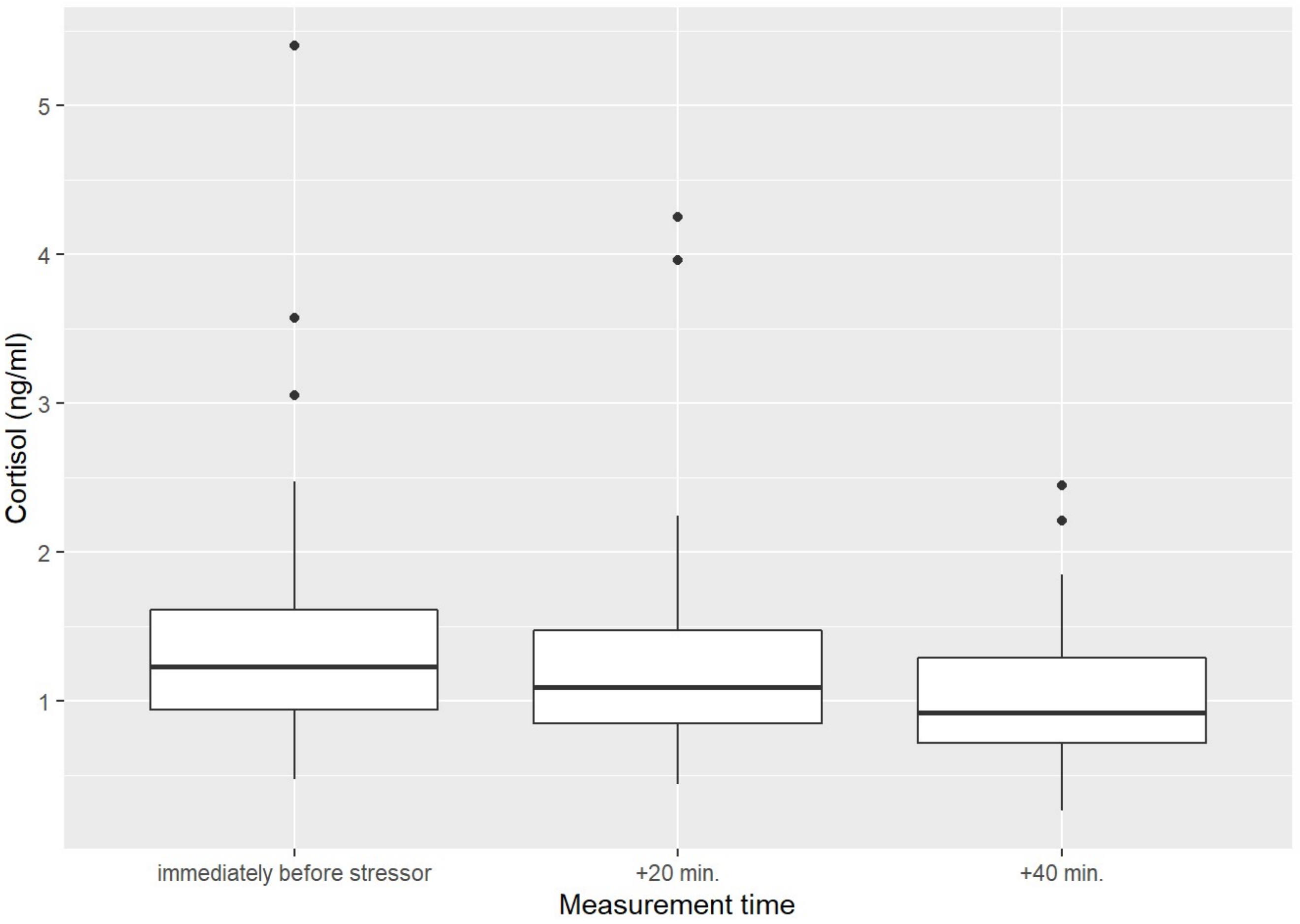


```
# Descriptives
## group-specific means
data_long %>%
   group_by(FST_numb) %>%
   summarise(across(c(cort_ng_ml),
                    list(mean = vm, sd = vsd, se = vse, min = vmin, max = vmax, n =
vnc)))
```

```
## # A tibble: 2 x 7
##   FST_numb cort_ng_ml_mean cort_ng_ml_sd cort_ng_ml_se cort_ng_ml_min
##   <fct>              <dbl>         <dbl>         <dbl>          <dbl>
## 1 Secure              1.15         0.719        0.0784           0.26
## 2 Insecure            1.52         0.813        0.142            0.68
## # ... with 2 more variables: cort_ng_ml_max <dbl>, cort_ng_ml_n <int>
```

```
## time-specific means
data_long %>%
   group_by(time) %>%
   summarise(across(c(cort_ng_ml),
                    list(mean = vm, sd = vsd, se = vse, min = vmin, max = vmax, n =
vnc)))
```

File failed to load: file:///C:/Users/mitho/Dokumente/B%C3%BCro/DFG-Angst/Paper%20Nathania/Repair_Attachment/manuscript/Review%

```
## # A tibble: 3 x 7
##   time                cort_ng_ml_mean cort_ng_ml_sd cort_ng_ml_se cort_ng_ml_min
##   <fct>                         <dbl>         <dbl>         <dbl>          <dbl>
## 1 immediately before~            1.45         0.910        0.146            0.47
## 2 +20 min.                       1.28         0.799        0.128            0.44
## 3 +40 min.                       1.03         0.466        0.0747           0.26
## # ... with 2 more variables: cort_ng_ml_max <dbl>, cort_ng_ml_n <int>
```

File failed to load: file:///C:/Users/mitho/Dokumente/B%C3%BCro/DFG-Angst/Paper%20Nathania/Repair_Attachment/manuscript/Review%
