# Supplementary material for: From Early Micro-Temporal Interaction Patterns to Child Cortisol Levels: Toward the Role of Interactive Reparation and Infant Attachment in a Longitudinal Study
Source: Front Psychol. 2022 Jan 20;12:807157. doi: 10.3389/fpsyg.2021.807157 (PMC8810635; doi:10.3389/fpsyg.2021.807157)
Supplement: Supplementary file 2 [file Table_1.pdf]

Table 1. *Excluded generalized binomial regression models on infant attachment out of hierarchical backward procedure*.

Model	Predictors	*OR*	95%*CI OR* lower bound	95%*CI OR* upper bound	*p*
Model 2	Intercept	0.075	0.003	1.050	/
	Anxiety disorder	0.378	0.000	65.418	0.021
	Interactive reparation	0.817	0.425	1.150	0.029
	Prepartum distress	1.034	0.888	1.186	0.039
	Anxiety disorder * Interactive reparation	1.637	0.895	5.031	0.204
	Anxiety disorder * Prepartum distress	1.019	0.873	1.216	0.442
	Anxiety disorder * Interactive reparation * Prepartum distress	0.997	0.984	1.009	0.373
Model 3	Intercept	0.058	0.007	0.265	/
	Anxiety disorder	0.658	0.011	13.201	0.021
	Interactive reparation	0.843	0.544	1.117	0.029
	Prepartum distress	1.048	0.991	1.135	0.039
	Anxiety disorder * Interactive reparation	1.557	0.941	4.191	0.204
	Anxiety disorder * Interactive reparation * Prepartum distress	0.997	0.985	1.009	0.285

Model 4	Intercept	0.058	0.008	0.245	/
	Anxiety disorder	0.273	0.006	5.047	0.021
	Interactive reparation	1.032	0.993	1.099	0.029
	Prepartum distress	1.062	1.007	1.140	0.039
	Anxiety disorder * Interactive reparation	1.121	0.962	1.587	0.204
Model 5	Intercept	0.065	0.011	0.251	/
	Anxiety disorder	0.830	0.061	8.062	0.021
	Interactive reparation	1.047	1.007	1.117	0.029
	Prepartum distress	1.050	1.002	1.113	0.039

Notes. *OR* = Odds ratio; *CI* = Confidence interval; *p* = empirical α-error; Model 2: *AIC* = 53.962, fitted probabilities numerically 0 or 1 occurred; Model 3: *AIC* = 52.016, fitted probabilities numerically 0 or 1 occurred; Model 4: *AIC* = 50.524, fitted probabilities numerically 0 or 1 occurred; Model 5: *AIC* = 50.134
