# Supplementary material for: From Early Micro-Temporal Interaction Patterns to Child Cortisol Levels: Toward the Role of Interactive Reparation and Infant Attachment in a Longitudinal Study
Source: Front Psychol. 2022 Jan 20;12:807157. doi: 10.3389/fpsyg.2021.807157 (PMC8810635; doi:10.3389/fpsyg.2021.807157)
Supplement: Supplementary file 3 [file Table_2.pdf]

Table 2. *Excluded Cox-regression models on match event out of hierarchical backward procedure.*

Model	Predictors	*HR*	95%*CI HR* lower bound	95%*CI HR* upper bound	*p*
Model 1	Insecure attachment	0.490	0.245	0.982	0.044
	Anxiety disorder	1.554	0.612	3.943	0.353
	Prepartum distress	0.989	0.970	1.007	0.226
Model 2	Insecure attachment	0.539	0.280	1.040	0.066
	Prepartum distress	0.995	0.981	1.008	0.446

Notes. *HR* = Hazard ratio; *CI* = Confidence interval; *p* = empirical α-error; Model 1: *LR* = 6.2, *df* = 3, *p* = 0.102; Model 1: *LR* = 5.37, *df* = 2, *p* = 0.068
