# Supplementary material for: From Early Micro-Temporal Interaction Patterns to Child Cortisol Levels: Toward the Role of Interactive Reparation and Infant Attachment in a Longitudinal Study
Source: Front Psychol. 2022 Jan 20;12:807157. doi: 10.3389/fpsyg.2021.807157 (PMC8810635; doi:10.3389/fpsyg.2021.807157)
Supplement: Supplementary file 4 [file Table_3.pdf]

**Table 3.** *Excluded mixed models on cortisol measures out of hierarchical backward procedure*.

| Model | Predictors | Sum of Squares | Mean Squares | Numerator *df* | Denominator *df* | *F* | *p* |
|---|---|---|---|---|---|---|---|
| Model 1 | Anxiety disorder | 0.098 | 0.098 | 1 | 34.000 | 0.343 | 0.562 |
| | Infant attachment | 0.957 | 0.957 | 1 | 34.000 | 3.339 | 0.076 |
| | Measurement time | 3.800 | 1.900 | 2 | 70.000 | 6.629 | 0.002 |
| | Cortisol baseline | 3.590 | 3.590 | 1 | 34.000 | 12.524 | 0.001 |
| | Anxiety disorder * Infant attachment | 0.394 | 0.394 | 1 | 34.000 | 1.376 | 0.249 |
| | Anxiety disorder * Measurement time | 0.022 | 0.011 | 2 | 70.000 | 0.039 | 0.962 |
| | Infant attachment * Measurement time | 0.610 | 0.305 | 2 | 70.000 | 1.064 | 0.351 |

| | | | | | | | |
|---|---|---|---|---|---|---|---|
| | Anxiety disorder * Infant attachment * Measurement time | 0.748 | 0.374 | 2 | 70.000 | 1.305 | 0.278 |
| Model 2 | Anxiety disorder | 0.098 | 0.098 | 1 | 34.000 | 0.343 | 0.562 |
| | Infant attachment | 1.336 | 1.336 | 1 | 49.611 | 4.663 | 0.036 |
| | Measurement time | 3.800 | 1.900 | 2 | 70.000 | 6.629 | 0.002 |
| | Cortisol baseline | 3.590 | 3.590 | 1 | 34.000 | 12.524 | 0.001 |
| | Anxiety disorder * Infant attachment | 0.087 | 0.087 | 1 | 79.958 | 0.304 | 0.583 |
| | Infant attachment * Measurement time | 1.242 | 0.621 | 2 | 70.000 | 2.167 | 0.122 |
| | Anxiety disorder * Infant attachment * Measurement time | 0.776 | 0.194 | 4 | 70.000 | 0.677 | 0.610 |
| Model 3 | Anxiety disorder | 0.096 | 0.096 | 1 | 34.000 | 0.343 | 0.562 |

| | | | | | | | |
|---|---|---|---|---|---|---|---|
| | Infant attachment | 0.940 | 0.940 | 1 | 34.000 | 3.339 | 0.076 |
| | Measurement time | 3.840 | 1.920 | 2 | 74.000 | 6.819 | 0.002 |
| | Cortisol baseline | 3.527 | 3.527 | 1 | 34.000 | 12.524 | 0.001 |
| | Anxiety disorder * Infant attachment | 0.388 | 0.388 | 1 | 34.000 | 1.376 | 0.249 |
| | Infant attachment * Measurement time | 0.810 | 0.405 | 2 | 74.000 | 1.439 | 0.244 |
| Model 4 | Infant attachment | 1.295 | 1.295 | 1 | 34.000 | 4.600 | 0.039 |
| | Measurement time | 3.840 | 1.920 | 2 | 74.000 | 6.819 | 0.002 |
| | Cortisol baseline | 3.527 | 3.527 | 1 | 34.000 | 12.524 | 0.001 |
| | Anxiety disorder * Infant attachment | 0.658 | 0.329 | 1 | 34.000 | 1.168 | 0.323 |

| | | | | | | | |
|---|---|---|---|---|---|---|---|
| | Infant attachment * Measurement time | 0.810 | 0.405 | 2 | 74.000 | 1.439 | 0.244 |
| Model 5 | Infant attachment | 1.666 | 1.666 | 1 | 36.000 | 5.916 | 0.020 |
| | Measurement time | 3.840 | 1.920 | 2 | 74.000 | 6.819 | 0.002 |
| | Cortisol baseline | 3.393 | 3.393 | 1 | 36.000 | 12.047 | 0.001 |
| | Infant attachment * Measurement time | 0.810 | 0.405 | 2 | 74.000 | 1.439 | 0.244 |

*Notes*. *Df* = Degrees of freedom, *F* = F-statistic, *p* = empirical α-error; Model 1: *REML* = 228.9; Model 2: *REML* = 228.9; Model 3: *REML* = 231.2; Model 4: *REML* = 231.2; Model 5: REML = 231.2
